# A rationally designed metal-binding helical peptoid for selective recognition processes†
†Electronic supplementary information (ESI) available. See DOI: 10.1039/c5sc04358a


**DOI:** 10.1039/c5sc04358a

**Published:** 2016-01-08

**Authors:** Maria Baskin, Galia Maayan

**Affiliations:** a Schulich Faculty of Chemistry , Technion-Israel Institute of Technology , Technion City , Hailfa 32000 , Israel . Email: gm92@tx.technion.ac.il

## Abstract

Metal-binding biopolymers play a significant role in processes, such as regulation, recognition and catalysis, due to their high affinity towards specific metal ions, which they bind selectively from the cellular pool. Many enzymes can bind two or more metal ions, each at a specific binding site, to enable efficient cooperative function. Imitating these recognition abilities might lead to the production of biomimetic materials such as unique chelators and catalysts. Herein, we report a rationally designed helical peptoid bearing two distinct metal binding ligands at positions *i* and *i* + 3 (**Helix HQT *i* + 3**), which enables the selective recognition of one or two metal ions depending on its environment. Using various spectroscopic techniques, we describe (1) the selective intramolecular binding of Cu^2+^ and its extraction from a mixture of neighboring metal ions in high concentrations, and (2) the selective intermolecular binding of two different metal ions, including the pair Cu^2+^ and Zn^2+^, one at each binding site, for the generation of hetero-bimetallic peptoid duplexes. Thorough analysis and comparison between the spectroscopic data and association constants of the metal complexes formed by **Helix HQT *i* + 3** and those formed by non-helical peptoids, or helical peptoids in which the two metal binding ligands are not pre-organized, revealed that the unique recognition processes performed by **Helix HQT *i* + 3** are controlled by both the sequence and the structure of the peptoid.

## Abstract 2

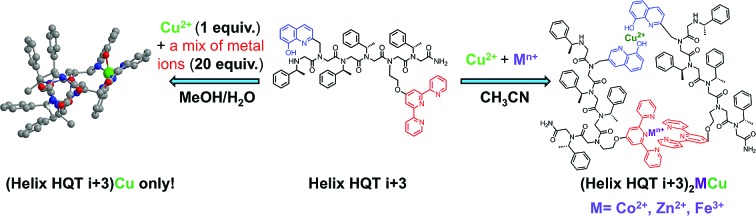
A helical peptoid bearing two distinct metal binding ligands at positions *i* and *i+3* (**Helix HQT i+3**) enables the selective recognition of one or two metal ions depending on its environment, thus mimicking the unique recognition abilities of natural biopolymers.

## Introduction

Metal ions are key elements in the structure and function of natural biopolymers, being employed in tasks spanning from signal transduction to electron transfer and catalysis. Biopolymers capable of binding metal ions exhibit high affinity and especially high selectivity towards specific metal ions that are required for their utility. Many metalloproteins found in nature have multiple coordination sites suitable for binding of at least two different metal ions, each at a distinct site, thus enabling cooperative and challenging catalytic tasks. Therefore, an important goal in the designing of metal-binding proteins, peptides and peptide mimics is to understand how to generate structures that selectively bind different metal ions in distinct sites.^1^ We note here that recognition processes for the metal ions Cu, Zn and Fe are especially interesting because these metals, being the three most abundant trace elements in biological and ecological systems, play central roles in the structure and function of proteins and all are essential components in many enzymes. Moreover, because there are only a few metal-binding side chains available for natural biopolymers, the high selectivity towards specific metal ion(s) is achieved by their 3-D structure (the folds), which controls intramolecular *vs.* intermolecular binding as well as steric and electronic effects. Mimicking such hetero bimetallic motifs by synthetic oligomers that can also fold might lead to unique cooperative catalysts in which each metal ion has a distinct role in the overall process towards high efficiency and selectivity.

In recent years, chemists started to explore possibilities to imitate these unique recognition properties, mostly by developing biomimetic foldamers^2^ capable of binding metal ions.^3^–^5^ Peptoids, *N*-substituted glycine oligomers, are peptidomimetics capable of forming stable helical structures that resemble the polyproline type helices if chiral bulky side groups are incorporated within their backbone.^6^ Peptoids can be easily synthesized on solid support using the “submonomer” method,^7^ which employs primary amines and thus does not require protection and de-protection steps and which enables the incorporation of innumerable functional groups at specified *N*-positions along their spine. Moreover, peptoids are biocompatible^8^ and both their sequences and secondary structures exhibit high stability.^9^ These features, specifically the great versatility of the peptoid backbone, which can be easily modified, thus have the potential to include various ligands for selective coordination of different metal ions and/or for the creation of different types of complexes, together with their ability to form secondary structures as an additional tool for controlling recognition, make them excellent candidates to carry out selective recognition processes.

Although peptoids research is generally well developed, its extension to metal coordination is still very limited. Currently, there are only a few examples of metallopeptoids, including, among others, a peptoid that mimics the zinc finger motif,5*a* peptoid chelators selective for Cr^6+^ or Cd^2+^,5g,l and peptoid catalysts bearing a phenanthroline ligand for the binding of Cu^+^.^10^ These examples demonstrate that metal-binding peptoids are not only excellent candidates for mimicking biological structural motifs, but they also exhibit a great potential for applications such as selective extraction of metal ions from various media and biomimetic cooperative catalysis. Despite these advances, the number of such examples is still very insufficient, and they are all currently limited to the binding of one metal ion per peptoid chelator. Moreover, the relationship between the peptoids sequence and their metal coordination properties has been scarcely explored.5*a* Herein, we describe for the first time, the rational design of a peptoid oligomer that can mimic the recognition ability of biopolymers by (i) selectively binding Cu^2+^ from a mixture of various neighboring metal ions in higher concentrations and (ii) binding two different metal ions selectively and simultaneously in two distinct coordination sites. We also demonstrate that these unique recognition capabilities are controlled by both the structure and sequence of the peptoid.

## Results and discussion

### Peptoid design for selective Cu^2+^, Cu^2+^/Zn^2+^ and Cu^2+^/Fe^3+^ recognition processes

**Scheme 1:**
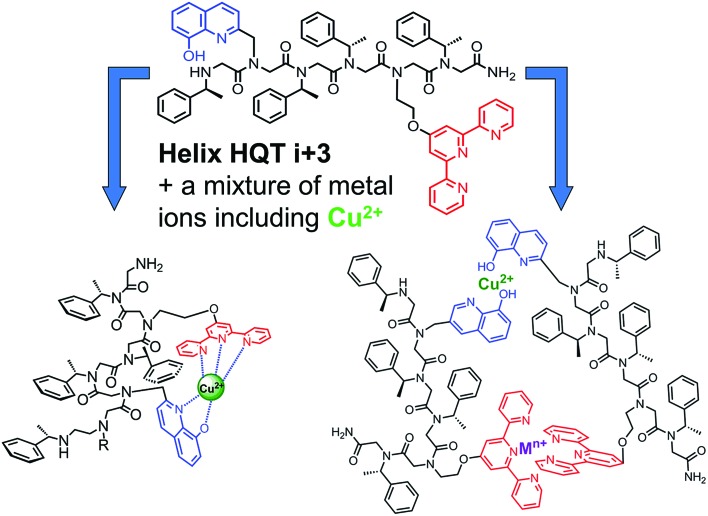
A rational design of a helical peptoid oligomer capable of intramolecular binding of one metal ion or intermolecular binding of two different metal ions in a selective manner.

It was previously shown that the pre-organization of two 8-hydroxyquinoline (HQ) ligands at positions *i* and *i* + 3 of a helical peptoid hexamer (**H_2_6**), such that they face the same side of the helix, led to the exclusive intramolecular coordination of Cu^2+^ with very high affinity (*K* > 10^14^ mol^–1^ in MeOH : H_2_O 4 : 1 (ref. 5*a*) and 1.43 ± 0.46 × 10^12^ mol^–1^ in MeOH : H_2_O 1 : 5 (ref. 11)). However, the intermolecular binding of two metal ions to form the peptoid duplex was not possible, probably because this product is less thermodynamically favored than the intramolecular complex, especially when the coordinative ligands are both identical and pre-organized. To control and study the recognition properties of metal-binding helical peptoids, we sought to start by replacing one HQ ligand in **H_2_6** with a different ligand targeting a peptoid that includes two distinct metal binding sites at positions *i* and *i* + 3*,* enabling metal coordination in both intramolecular and intermolecular modes. For this purpose, we chose 2,2′:6′,2′′-terpyridine (Terpy) as the second ligand, resulting in the design of peptoid **Helix HQT *i* + 3** (Scheme 1). We assumed that this peptoid would have high affinity and high selectivity for Cu^2+^*via* intramolecular binding to both HQ and Terpy because such coordination can lead to a square pyramid geometry, which can be stabilized by Cu^2+^ but not by neighboring metal ions, such as Zn^2+^, Fe^3+^, Co^2+^, Ni^2+^ and Mn^2+^, that can also bind these ligands.^12^ Moreover, as this peptoid includes two distinct binding sites, we anticipated that intermolecular coordination of two different metal ions would be also possible, probably under modified reaction conditions. Such binding will lead to the production of unique hetero-bimetallic peptoid duplexes in which Cu^2+^ is bound to two HQ ligands, stabilizing a tetragonal geometry, and a second metal ion, *e.g.* Zn^2+^, Fe^3+^ or Co^2+^, is bound to two Terpy ligands, stabilizing an octahedral geometry (see Scheme 1).12c,d

### Synthesis and characterization of the peptoid **Helix HQT *i* + 3** and its Cu^2+^ complex

**Fig. 1:**
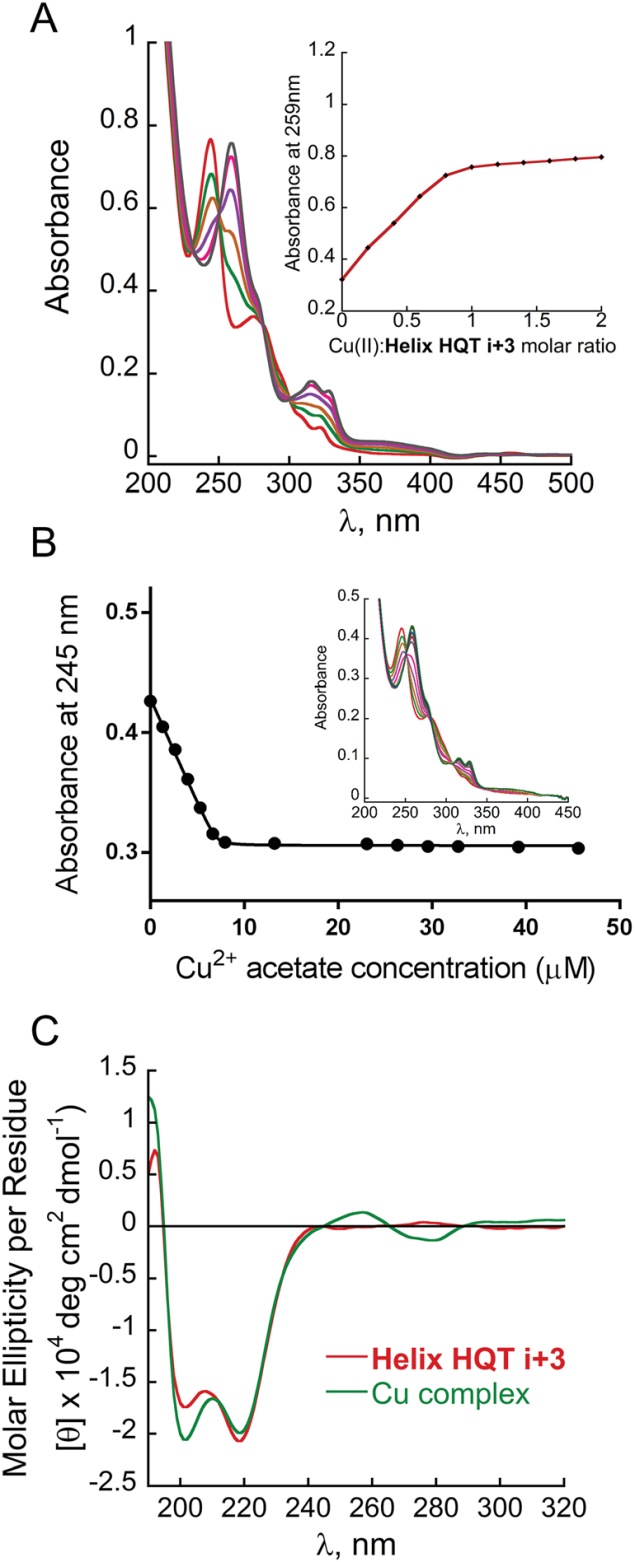
(A) UV-Vis spectra and peptoid-to-metal ratio plot for the titration of **Helix HQT *i* + 3** with Cu^2+^. The peptoid (17 μM) in MeOH : H_2_O 4 : 1 solution was titrated with 2 μL aliquots of a metal ion (5 mM in H_2_O) in multiple steps (red = free ligand, black = metal complex). (B) Non-linear regression fit and UV-Vis spectra for the titration of **Helix HQT *i* + 3** with Cu^2+^. The peptoid (7 μM) in MeOH : H_2_O 1 : 5 solution was titrated with 2 μL aliquots of a metal ion (2 mM in H_2_O) in multiple steps (red = free ligand, black = metal complex). (C) CD spectra of **Helix HQT *i* + 3** (100 μM in MeOH : H_2_O 4 : 1) before (red) and after (green) the addition of 1 equivalent Cu^2+^.

The peptoid **Helix HQT *i* + 3** was synthesized on Rink amide resin using a previously reported variation of the peptoid submonomer protocol,^13^ cleaved from the solid support and purified by HPLC (>99% purity). The molecular weight measured by electrospray ionization mass spectrometry (ESI MS) was consistent with the expected mass. Metal-free peptoid **Helix HQT *i* + 3** exhibits absorption bands near *λ* = 245 and 278 nm, in MeOH : H_2_O 4 : 1, arising from the ligands HQ and Terpy, respectively. Upon addition of copper acetate, these two bands diminished simultaneously and new absorption bands near *λ* = 259, 316 and 328 nm were produced (Fig. 1A). A peptoid-to-metal ratio plot constructed from these UV-Vis titrations was consistent with a 1 : 1 peptoid : Cu ratio, demonstrating the formation of the intramolecular complex (**Helix HQT *i* + 3**)Cu (Fig. 1A, inset). The peptoid-to-metal ratio was verified by ESI MS and the isotopic analysis showed no evidence for the formation of higher order complexes (*e.g.* 2 : 2 complexes, see ESI†).

**Fig. 2:**
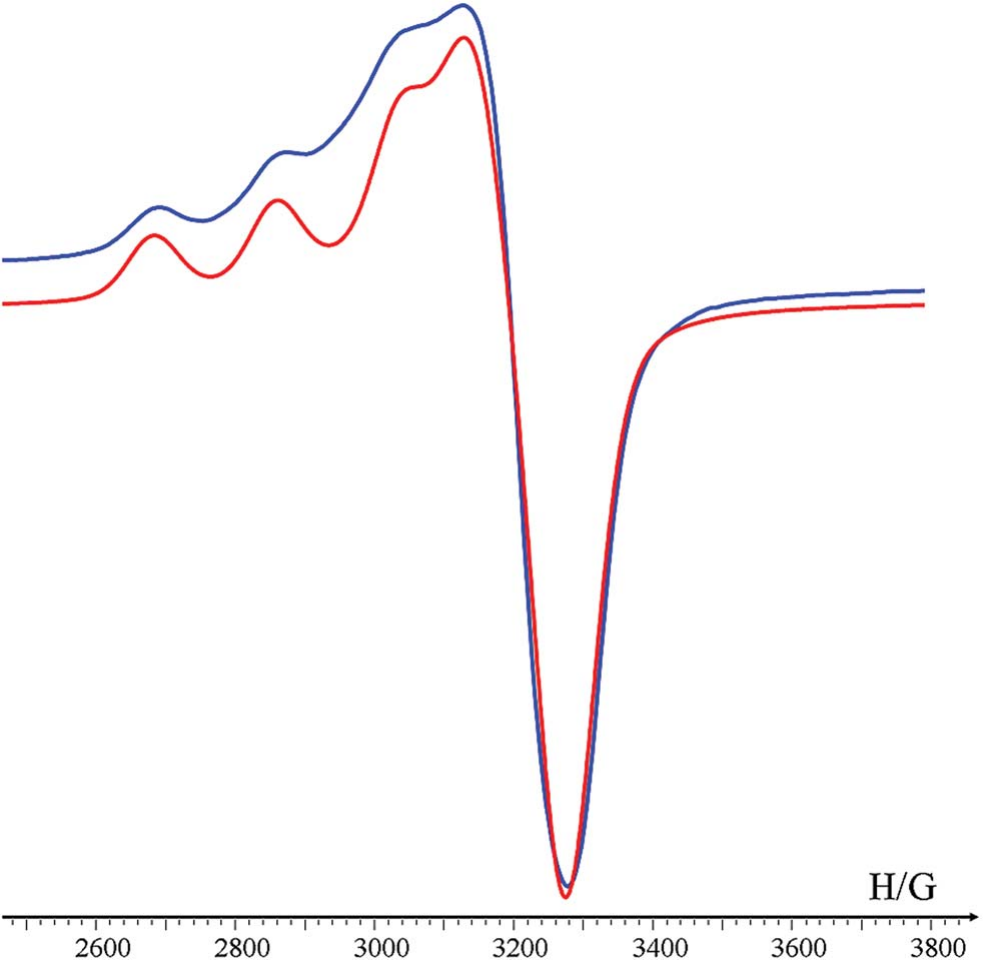
X-band EPR spectrum of (**Helix HQT *i* + 3**)Cu (blue line) and its corresponding simulated spectrum (red line). The measurements were performed in the solid state at rt., with TEMPO as a reference (*g* = 2.0058).

The association constant of (**Helix HQT *i* + 3**)Cu was calculated by a nonlinear regression curve fitting obtained from UV titration experiments at lower concentrations in MeOH : H_2_O 1 : 5 (Fig. 1B). The value for the formation of (**Helix HQT *i* + 3**)Cu is *K* = 1.03 ± 0.49 × 10^13^ M^–1^. This value reflects a strong binding affinity, which is higher by one order of magnitude than that of **H_2_6**Cu in the same titration conditions, thus supporting our design principles. The circular dichroism (CD) spectrum of **Helix HQT *i* + 3** in aqueous methanol showed double minima near *λ* = 202 and 220 nm, which is characteristic of a peptoid helix6c,d (Fig. 1C, red line). Adding 1 equivalent of Cu^2+^ to the CD cuvette produced exciton couplet CD peaks between 240 and 300 nm, the region corresponding to the 8-hydroxyquinoline π–π* transition, with a maximum at 257 nm and a minimum at 279 nm, crossing *ε* = 0 near 265 nm.5b,k EPR measurements of the solid (**Helix HQT *i* + 3**)Cu complex indicated the presence of Cu^2+^ (Fig. 2) and the Hamiltonian parameters obtained from the simulated spectra were *g*_∥_ = 2.23, *g*_⊥_ = 2.070 and *A*_∥_ = 175 G, which are consistent with a square pyramidal coordination geometry.^14^

### Selective recognition of Cu^2+^ by **Helix HQT *i* + 3**

**Fig. 3:**
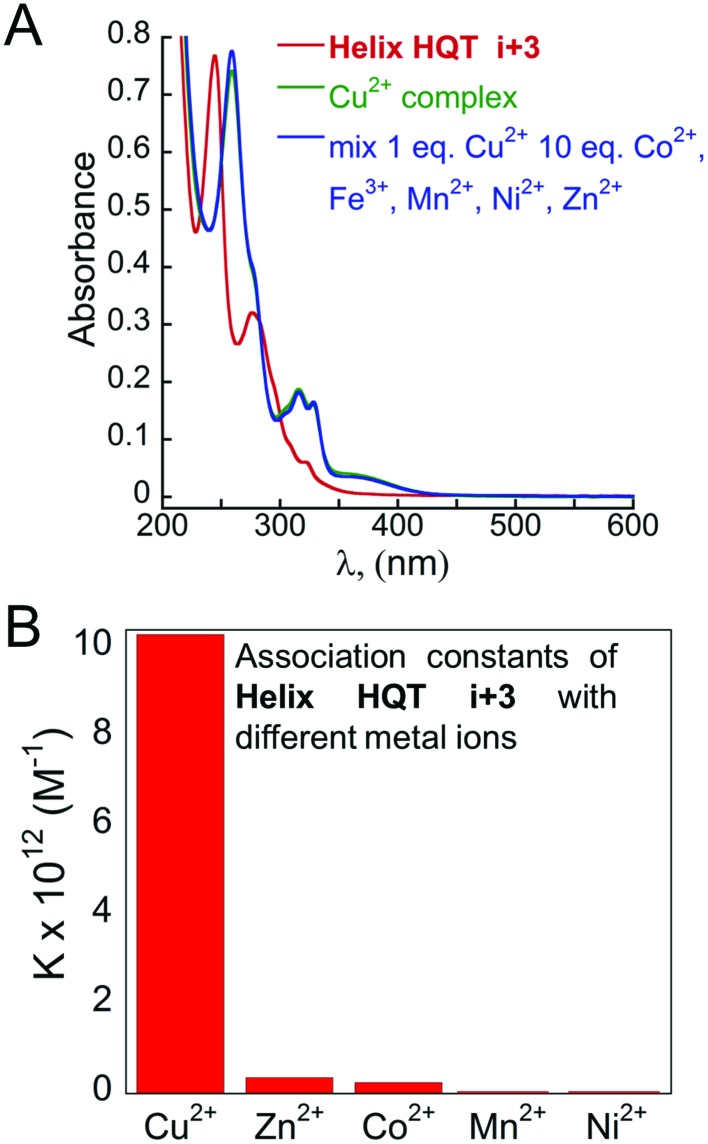
Selective binding of Cu^2+^ in aqueous methanol: (A) UV-Vis spectra of **Helix HQT *i* + 3** (red line), its Cu complex (green line) and the complex formed from a mixture solution of 1 : 10 Cu^2+^ : other metal ions (blue line). (B) Association constants for the formation of (**Helix HQT *i* + 3**)M complexes.

The initial selectivity assessment of **Helix HQT *i* + 3** towards Cu^2+^ was conducted by treating the peptoid with a 1 equivalent mixture solution containing the metal ions Cu^2+^, Co^2+^, Zn^2+^, Fe^3+^, Mn^2+^ and Ni^2+^ in MeOH : H_2_O 4 : 1. Interestingly, the UV spectrum of this solution was identical to the UV spectrum of (**Helix HQT *i* + 3**)Cu, suggesting that this peptoid exhibits high selectivity towards Cu^2+^ from the tested mixture. ESI MS analysis of this mixture solution displayed only the mass of the Cu complex, supporting the selective binding of Cu^2+^ from the mixture solution (Fig. S77†). For comparison, the formation of (**Helix HQT *i* + 3**)M (M = Co^2+^, Zn^2+^, Fe^3+^, Mn^2+^ or Ni^2+^) was characterized by UV-Vis titrations and by ESI MS under the same reaction conditions. The peptoid-to-metal ratio plots revealed 1 : 1 intramolecular binding with all the metal ions except with Fe^3+^, in which the binding was intermolecular, with distinct absorption bands for each metal complex; the ESI MS analysis reflected the mass of each complex (see ESI†). These results support the observation that the UV-Vis and ESI MS spectra corresponding to the reaction of **Helix HQT *i* + 3** with the mixture solution produced only the Cu^2+^ complex. To further evaluate the selectivity of **Helix HQT *i* + 3** towards Cu^2+^, we tested its binding in mixtures containing higher concentrations of the different metal ions relative to Cu^2+^, *i.e.* 1 equivalent Cu^2+^ and up to 20 equivalents of Co^2+^, Zn^2+^, Fe^3+^, Mn^2+^ and Ni^2+^ mixture, using UV-Vis and ESI-MS. The obtained UV-Vis spectra were identical to the spectrum of (**Helix HQT *i* + 3**)Cu in all the examined ratios (see Fig. 3A for the ratio 1 : 10) and the ESI MS spectra demonstrated exclusively the mass of (**Helix HQT *i* + 3**)Cu.

To quantify these results, we calculated the association constants of **Helix HQT *i* + 3** with each metal ion (Fig. 3B).^15^ The values for the formation of (**Helix HQT *i* + 3**)M (M = Zn^2+^, Co^2+^, Ni^2+^ and Mn^2+^) were *K* = 3.60 ± 0.46 × 10^11^, 2.53 ± 0.35 × 10^11^, 1.52 ± 0.09 × 10^10^ and 1.13 ± 0.06 × 10^10^ M^–1^, respectively. These results clearly demonstrate that Cu^2+^ coordination is at least one order of magnitude higher than that of the other metal ions, consistent with the high selectivity observed when binding from the mixture solutions. According to these data, selective binding of Cu^2+^ can occur from solutions containing about 27, 40, 670 and 885 times higher concentrations of Zn^2+^, Co^2+^, Ni^2+^ and Mn^2+^, respectively. To validate these findings, we obtained the UV spectra of solutions containing a mixture of 1 equivalent of Cu^2+^ and 22–27 equivalent of Zn^2+^, as well as solutions containing a mixture of 1 equivalent Cu^2+^ and 35–40 equivalent of Co^2+^ (see ESI†). The results indicated that selectivity for Cu^2+^ is retained in solutions having 25 equivalent of Zn^2+^ and 35 equivalent of Co^2+^, which in both cases is consistent with the calculated binding constants.

Finally, we were interested in investigating whether the selectivity for Cu^2+^ is thermodynamically or kinetically driven. Thus, we decided to examine the ability of Cu^2+^ to replace any other metal ion pre-bound to **Helix HQT *i* + 3**. For this purpose, the complexes (**Helix HQT *i* + 3**)M (M = Co^2+^, Zn^2+^, Mn^2+^ or Ni^2+^) in a UV cuvette were treated with 1 equivalent of Cu^2+^ in aqueous methanol and the UV spectra were obtained before and after the addition of Cu^2+^. The final UV-Vis spectra revealed that Cu^2+^ was able to replace the metal ions Zn^2+^ and Mn^2+^, forming the thermodynamically stable complex (**Helix HQT *i* + 3**)Cu^2+^, whereas the complexes (**Helix HQT *i* + 3**)Co^2+^ and (**Helix HQT *i* + 3**)Ni^2+^ remained intact (Fig. S17–20†). From this experiment, we can propose that the selectivity for Cu^2+^ is thermodynamically driven with regard to Zn^2+^ and Mn^2+^, and kinetically driven with regard to Co^2+^ and Ni^2+^.

### Extraction of Cu^2+^ from a mixture solution by helix **HQT *i* + 3**

One immediate application for this selective recognition of Cu^2+^ is its extraction from a mixed metal ions solution. Selective extraction of metal ions, and specifically of Cu^2+^, is important for processes such as metal ion(s) overload that is toxic to living cells,^16^ Cu^2+^ contamination in cell cultures, and chemical reactions that involve several metal reagents or catalysts, which require the removal of Cu^2+^ at any stage of the reaction. Reports about chelators that show high selectivity for Cu^2+^ (ref. 17) focus on its detection in cells with the aim of studying its mechanism of action, rather than focusing on its extraction from various media. Recently, several peptoid chelators were described, demonstrating the removal of Cr^6+^ from aqueous media5*g* and of Cd^2+^ from biological media,5*l* but not of Cu^2+^. These peptoids were identified using combinatorial peptoid libraries rather than *via* rational design, which is in the heart of this study.

**Fig. 4:**
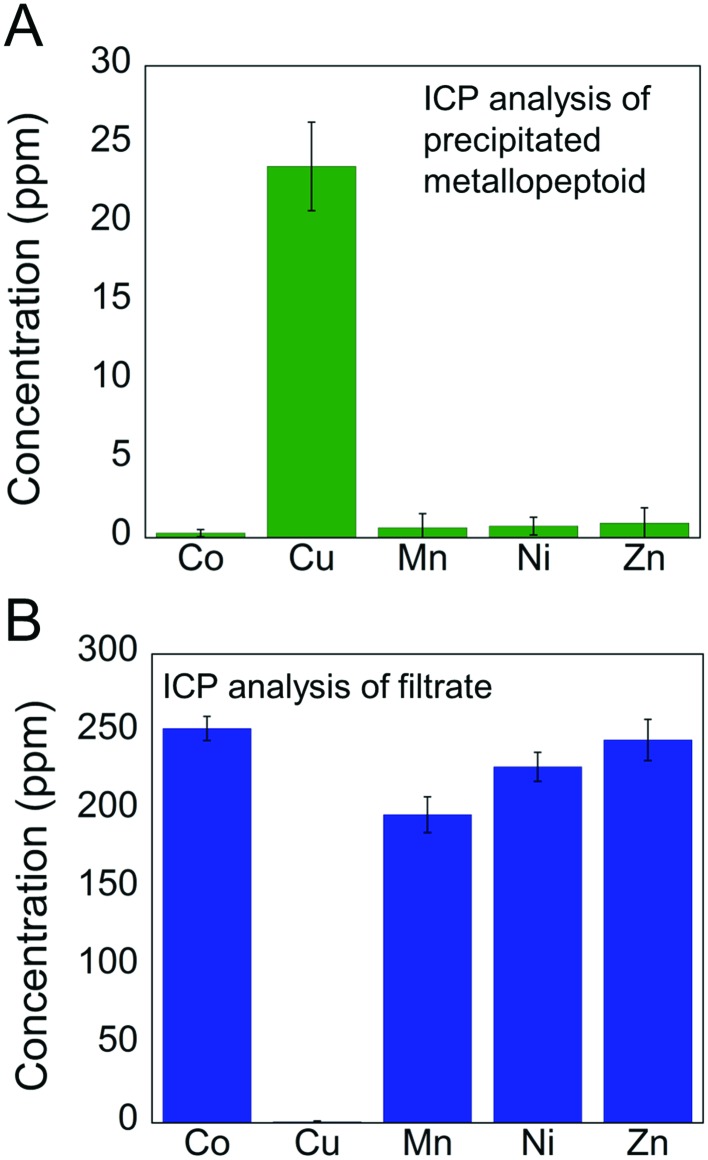
Selective extraction of Cu^2+^: (A) ICP analysis of the precipitate and (B) of the filtrate obtained from the reaction of **Helix HQT *i* + 3** with a mixture of Cu^2+^ (1 equivalent, 0.5 mM) and the metal ions Co^2+^, Mn^2+^, Ni^2+^ and Zn^2+^ (10 equivalent, 5 mM each). Standard errors are represented by error bars, with experiments number = 3.

The selective extraction of Cu^2+^ from a mixture solution containing 1 equivalent of Cu^2+^ and 10 equivalents of Co^2+^, Zn^2+^, Mn^2+^ and Ni^2+^ was estimated by inductively coupled plasma (ICP) measurements.^18^ Following the reaction of **Helix HQT *i* + 3** with the mixture solution in aqueous methanol, the solvent was removed, the solid residue was re-dissolved in water and the obtained precipitate was separated from the solution by centrifugation. ICP analysis of the precipitated metallopeptoid revealed the exclusive presence of Cu with negligible amounts of the other metals (Fig. 4A). ICP analysis of the filtrate showed high concentrations of Co, Zn, Mn and Ni together with an insignificant amount of Cu (Fig. 4B). The ICP experiments demonstrate the biomimicry of **Helix HQT *i* + 3** being able not only to select a specific metal ion from a mixture containing high concentrations of neighboring metal ions, but also to remove it from this mixture solution, leaving the other metal ions intact, even when it is present in much smaller concentrations than the other metal ions.

### The role of peptoid helicity and ligands pre-organization in the selective recognition of Cu^2+^

**Fig. 5:**
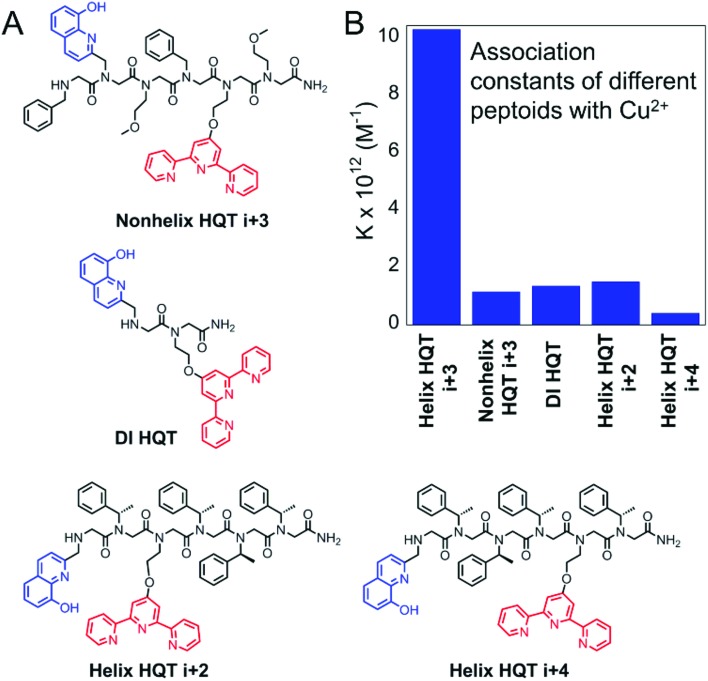
Sequences of the control peptoids (A) and their association constants for the formation of their corresponding Cu complexes (B).

Furthermore, we wished to explore whether the high affinity and selectivity of **Helix HQT *i* + 3** towards Cu^2+^ arises from its secondary structure and/or from the pre-organization of HQ and Terpy at positions *i* and *i* + 3. For this purpose, four control peptoids were synthesized and purified: the unstructured hexamer **Nonhelix HQT *i* + 3**, containing HQ and Terpy at positions *i* and *i* + 3 in addition to the non-structure directing groups benzyl and methoxyethyl in the other positions along the backbone; the dimer **DI HQT**, having only the two ligands HQ and Terpy; and the two helical hexamers **Helix HQT *i* + 2** and **Helix HQT *i* + 4** in which HQ and Terpy are not pre-organized to face the same side of helix (Fig. 5A). The association constants of the control peptoids with Cu^2+^ were calculated under the same conditions as described above and the values obtained for the formation of (**Nonhelix HQT *i* + 3**)Cu, (**DI HQT**)Cu, (**Helix HQT *i* + 2**)Cu and (**Helix HQT *i* + 4**)Cu were *K* = 1.16 ± 0.53 × 10^12^, 1.36 ± 0.66 × 10^12^, 1.50 ± 0.37 × 10^12^ and 4.12 ± 0.84 × 10^11^ M^–1^, respectively (Fig. 5B). All these values were about one order of magnitude smaller compared with the value calculated for (**Helix HQT *i* + 3**)Cu, suggesting that both the helicity and the ligands' pre-organization play a role in the high selectivity of **Helix HQT *i* + 3** towards the binding of Cu^2+^.

EPR measurements, conducted in the solid state at rt., indicated the presence of Cu^2+^ in all four complexes. The values of the hyperfine splitting for (**Nonhelix HQT *i* + 3**)Cu, (**DI HQT**)Cu, (**Helix HQT *i* + 2**)Cu and (**Helix HQT *i* + 4**)Cu were 162 G, 166 G, 150 G and 158 G, respectively (Table 1). All these values are lower than the value for (**Helix HQT *i* + 3**)Cu (175 G), implying that some (or all) of the control peptoid complexes exhibit tetragonal geometries.^1^ To probe this point, we conducted an EPR measurement for **H_2_6**, which has two HQ ligands and was shown to bind Cu^2+^ in a pseudotetrahedral geometry.5*b* The measurement, which was done in the same conditions as with the other (peptoid)Cu complexes, provided a value of 150 G (Table 1), which is identical to that of (**Helix HQT *i* + 2**)Cu and close to the hyperfine splitting measured for (**Helix HQT *i* + 4**)Cu. It was previously suggested that the trans-planar configuration of Cu(hydroxyquinolate)_2_ is not possible in the intramolecular (**H_2_6**)Cu complex because of the steric interactions arising from its helical structure that prohibit a square planar geometry.5*b* We therefore suggest that in the case of the sterically hindered helices (**Helix HQT *i* + 2**) and (**Helix HQT *i* + 4**), in which HQ and Terpy are *not* pre-organized, their Cu^2+^ complexes adopt tetragonal coordination geometries (*i.e.* by binding to only four out of the five N- and O-coordination atoms) because their hyperfine splitting values resemble the value of the pseudotetrahedral (**H_2_6**)Cu complex, and therefore they are less selective towards Cu^2+^.

**Table 1:** EPR parameters of the intramolecular (peptoid)Cu complexes^*a*^

Complex/EPR data	*A*_∥_[G]	*g*_⊥_	*g*_∥_
(**Helix HQT *i* + 3**)Cu	175	2.070	2.23
(**Nonhelix HQT *i* + 3**)Cu	162	2.070	2.23
(**DI HQT**)Cu	166	2.070	2.21
(**Helix HQT *i* + 2**)Cu	150	2.070	2.25
(**Helix HQT *i* + 4**)Cu	158	2.070	2.25
(**H_2_6**)Cu	150	2.071	2.25

^*a*^All measurements were performed in the solid state at rt., with TEMPO as a reference (*g* = 2.0058).

To evaluate the selectivity of the four peptoids towards Cu^2+^ from a mixture solution containing the metal ions Cu^2+^, Co^2+^, Zn^2+^, Fe^3+^, Mn^2+^ and Ni^2+^, 1 equivalent of that mixture in MeOH : H_2_O 4 : 1 was added to each one of these four peptoids and the UV-Vis spectra were obtained. The obtained four UV-Vis spectra were different from the spectra of their copper complexes, suggesting that there is no selective recognition of Cu^2+^ by these four peptoids. These results were further proved by ESI MS analysis of these solutions, which demonstrated the formation of other metal complexes in addition to (**Helix HQT *i* + 3**)Cu; for example, the ESI MS spectrum of **Nonhelix HQT *i* + 3** showed the mass of a Co^2+^ complex in addition to the Cu^2+^ complex, and the spectrum of **DI HQT** showed the mass of a Zn^2+^ complex in addition to the Cu^2+^ complex. Moreover, calculating the association constants of these four control peptoids with each metal ion from the mixture^15^ showed that the values for the formation of the complexes with Cu^2+^, Co^2+^ and Zn^2+^ are all in the same order of magnitude, supporting the low selectivity for Cu^2+^ (Table S4†). Overall, our observations support the biomimetic character of our system, demonstrating that control over both the helical structure and the pre-organization of HQ and Terpy is crucial for recognition.

### Selective recognition of two different metal ions by helix **HQT *i* + 3**

**Fig. 6:**
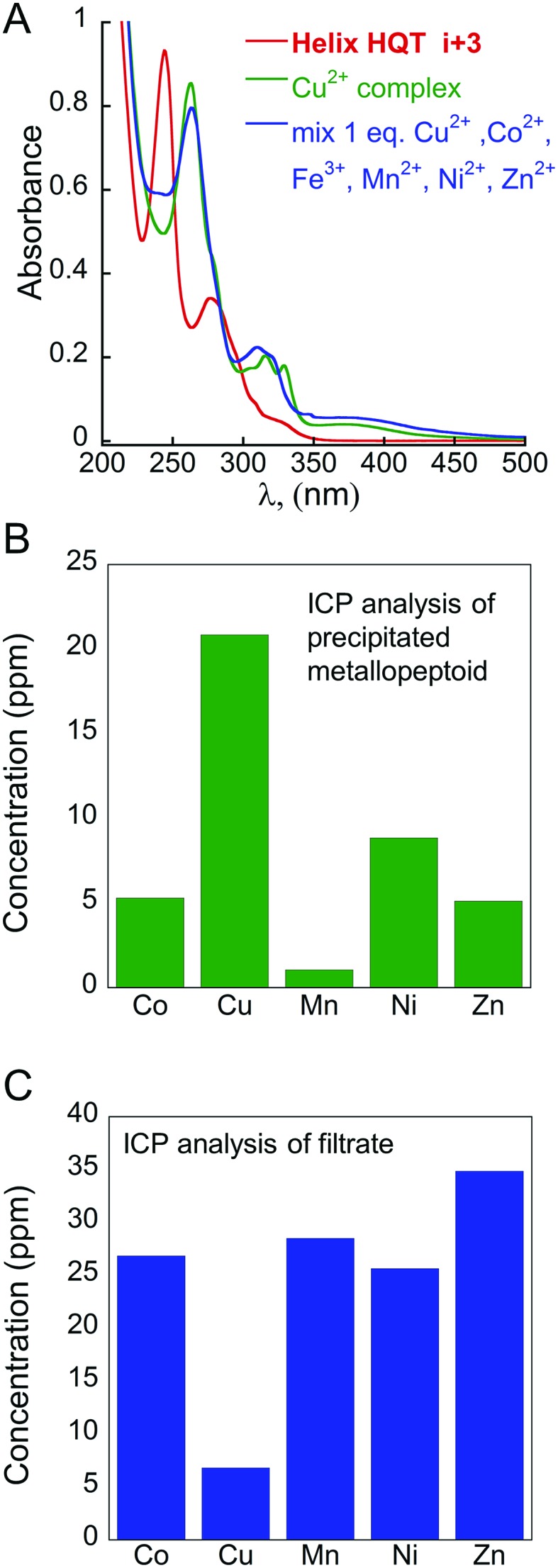
Non-selective binding of Cu^2+^ in acetonitrile: (A) UV-Vis spectra of **Helix HQT *i* + 3** (red line), its Cu complex (green line) and the complex formed from a mixture solution of 1 : 1 Cu^2+^ : other metal ions (blue line). (B) ICP analysis of the precipitate and (C) filtrate obtained from the reaction of **Helix HQT *i* + 3** with a mixture of Cu^2+^ (1 equivalent, 1 mM) and the metal ions Co^2+^, Mn^2+^, Ni^2+^ and Zn^2+^ (1 equivalent, 1 mM each).

According to the abovementioned results, only intramolecular binding of all the tested metal ions (excluding Fe^3+^) by **Helix HQT *i* + 3** can be achieved in aqueous methanol. One possible explanation could be that both methanol and water are able to coordinate as an additional ligand to the metal ions that do not stabilize the square pyramid geometry (*e.g.* cobalt ions), forming other stable penta- or hexa-coordinated complexes (*e.g.* octahedral geometry). Thus, the selective intermolecular binding of two different metal ions in distinct binding sites is impossible in these conditions. To enable such intermolecular binding, we sought to conduct the binding experiments in a different solvent that is less coordinative than water and/or methanol such as acetonitrile.^19^ Indeed, adding 1 equivalent mixture solution containing the metal ions Cu^2+^, Co^2+^, Zn^2+^, Fe^3+^, Mn^2+^ and Ni^2+^ in acetonitrile to **Helix HQT *i* + 3** produced a UV-Vis spectrum that was different from the spectrum of (**Helix HQT *i* + 3**)Cu (Fig. 6A), suggesting that the binding in acetonitrile is not selective towards Cu^2+^. This was further supported by ICP experiments showing low selectivity for Cu^2+^ in acetonitrile (Fig. 6B and C). In addition, to facilitate intermolecular binding, we thought to explore it using two different approaches: (i) the step approach, in which 1 equivalent of a metal ion other than Cu^2+^, *e.g.* Zn^2+^ or Fe^3+^, will be added to 2 equivalents of the peptoid, aiming for selective binding to two Terpy ligands, followed by the addition of Cu^2+^ to be bound to the two HQ ligands (kinetic control); and (ii) the mixture approach, in which the peptoid will be treated with a mixture solution containing 0.5 equivalent of Cu^2+^ and 0.5 equivalent of the other metal ion under some heating and/or longer reaction time (thermodynamic control), targeting simultaneous binding of the two ions: Cu^2+^ to two HQ ligands and the other ion to two Terpy ligands.

**Fig. 7:**
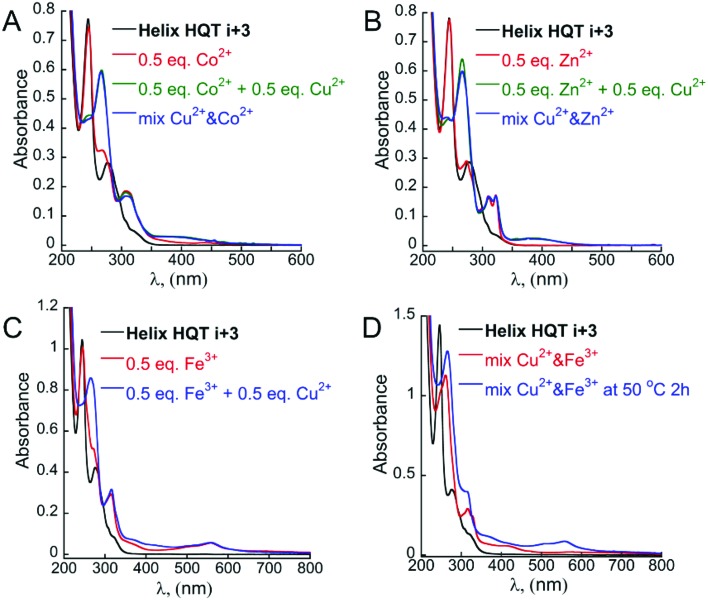
UV-Vis spectra of **Helix HQT *i* + 3** (black line), its 2 : 1 peptoid : M Co (A), Zn (B) or Fe (C) complexes (red line) and its 2 : 2 peptoid : M complexes formed *via* the step approach (A and B green lines and C, blue line) or the mixture approach (A and B blue lines; D, red and blue lines).

Starting with the step approach, 1 equivalent of metal-free peptoid **Helix HQT *i* + 3** in acetonitrile was treated with 0.5 equivalent of Co^2+^, Zn^2+^, Fe^3+^, Ni^2+^ or Mn^2+^ in a UV cuvette, and the changes in the absorption bands near *λ* = 245 and 278 nm of HQ and Terpy ligands, respectively, were recorded. Upon addition of Co^2+^, Zn^2+^ or Fe^3+^, the band near 278 nm disappeared and new absorption bands near *λ* = 307 nm, *λ* = 312 and 323 nm, and *λ* = 316 and 559 nm, respectively, were obtained (Fig. 7A–C, black and red lines). It can be noted that no change in the absorption band near *λ* = 245 nm was recorded, reflecting the exclusive binding of each of these three metal ions to Terpy and the formation of (Terpy)_2_M complexes (see ESI†).^20^ In contrast, upon addition of either Ni^2+^ or Mn^2+^, the absorbance bands of both Terpy and HQ disappeared simultaneously and new bands near *λ* = 270, 312 and 325 nm, and *λ* = 263, 312 and 323 nm, respectively, were obtained, reflecting the intramolecular binding of these ions and the formation of complexes of the type (**Helix HQT *i* + 3**)M. Subsequent addition of 0.5 equivalents of Cu^2+^ to each of the (Terpy)_2_M complexes (M = Co^2+^, Zn^2+^ or Fe^3+^) resulted in the disappearance of the band at 245 nm and the appearance of a new absorption band at *λ* = 266 nm, indicating the formation of (HQ)_2_Cu complex (Fig. 7A and B, green lines and 7C, blue line).

Overall, this approach enabled the formation of the heteronuclear bimetallic duplexes (**Helix HQT *i* + 3**)_2_MCu (M = Co^2+^, Zn^2+^ or Fe^3+^) as demonstrated by the UV-Vis spectra and supported by MS analysis (ESI†). The CD spectra of (**Helix HQT *i* + 3**)_2_MCu (M = Co^2+^, Zn^2+^ or Fe^3+^) in acetonitrile was similar to that of (**Helix HQT *i* + 3**)Cu that was measured in aqueous methanol, showing a double minima characteristic of a peptoid helix and exciton couplet CD peaks (Fig. S8†). EPR analysis of (**Helix HQT *i* + 3**)_2_ZnCu in the solid state at rt. clearly indicated the presence of Cu in this complex. The Hamiltonian parameters obtained from the simulated spectra were *g*_∥_ = 2.24, *g*_⊥_ = 2.065 and *A*_∥_ = 155 G. It can be noted that this hyperfine splitting resembles the one obtained for the tetragonal complex (**H_2_6**)Cu (150 G, Table 1), supporting our observations from the UV-Vis spectra that copper is bound to the two HQ ligands of the hetero bimetallic complex (**Helix HQT *i* + 3**)_2_ZnCu.

**Fig. 8:**
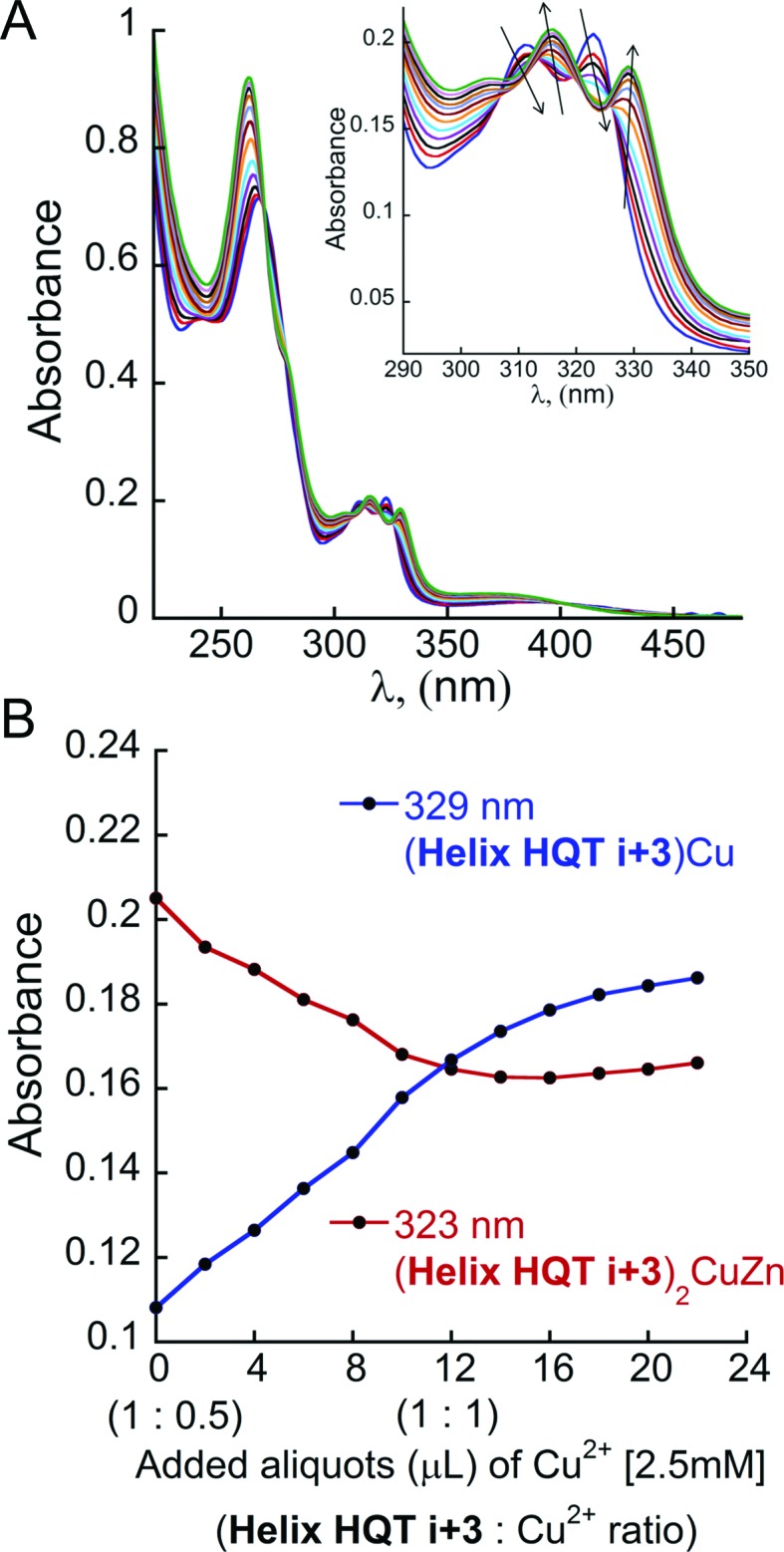
(A) UV-Vis titration of (**Helix HQT *i* + 3**)_2_ZnCu (blue line, 8 μM) with Cu^2+^ (2 μL aliquots of 2.5 mM) in 3 mL acetonitrile. Inset: the UV-Vis spectrum in the region between 290 and 350 nm. (B) Representation of the decrease in the absorbance of (**Helix HQT *i* + 3**)_2_ZnCu complex at *λ* = 323 nm (red line) and the simultaneous increase in the absorbance of (**Helix HQT *i* + 3**)Cu complex at *λ* = 329 nm (blue line) during the titration.

Interestingly, titrating the acetonitrile solution of (**Helix HQT *i* + 3**)_2_ZnCu with additional aliquots of Cu^2+^ solution resulted in a gradual shift in the absorbance bands until the full disappearance of the bands at *λ* = 266, 312 and 323 nm, corresponding to the complex (**Helix HQT *i* + 3**)ZnCu, and the appearance of bands near *λ* = 262, 316 and 329 nm (Fig. 8), which indicated the formation of the complex (**Helix HQT *i* + 3**)Cu. These results demonstrate the biomimetic ability of **Helix HQT *i* + 3** to modify its binding mode by adjusting it to changes in its environment (*i.e.* excess of one metal ion over another one). Similar titrations of (**Helix HQT *i* + 3**)_2_CoCu and (**Helix HQT *i* + 3**)_2_FeCu did not lead to any significant changes in their UV-Vis spectra, suggesting that these complexes are more stable as duplexes, probably due to the highly stable octahedral geometry that both ions form with 2 equivalents of Terpy.

Continuing with the mixture approach, 1 equivalent of metal-free peptoid **Helix HQT *i* + 3** in acetonitrile was treated with a mixture containing 0.5 equivalents (from each ion) of (i) Co^2+^ and Cu^2+^, (ii) Zn^2+^ and Cu^2+^ or (iii) Fe^3+^ and Cu^2+^ in a UV cuvette. We then followed the changes in the absorption bands near *λ* = 245 and 278 nm of HQ and Terpy ligands, respectively. In the case of Co^2+^ and Cu^2+^, we were pleased to observe that both of these bands disappeared simultaneously, while two new bands near *λ* = 266 and 307 nm appeared, indicating the generation of the complexes (HQ)_2_Cu and (Terpy)_2_Co and the overall formation of the metallopeptoid duplex (**Helix HQT *i* + 3**)_2_CoCu (Fig. 7A, blue line). It can be noted that this spectrum was identical to the spectrum obtained when Co^2+^ and Cu^2+^ were added separately to **Helix HQT *i* + 3** following the step approach. The existence of (**Helix HQT *i* + 3**)_2_CoCu was further confirmed by ESI-MS (ESI†). The same observations were obtained with the mixture solution of Zn^2+^ and Cu^2+^ (Fig. 7B, blue line), indicating that also in this case a metallopeptoid duplex binding the two different metal ions in distinct sites was formed, and the existence of (**Helix HQT *i* + 3**)_2_ZnCu was further confirmed by ESI-MS (ESI†). Using the mixture solution of Fe^3+^ and Cu^2+^, however, resulted in the appearance of absorbance bands near *λ* = 262, 316 and 328 nm, whereas the absorbance band near *λ* = 559 was missing (Fig. 7D, red line). In fact, this UV-Vis spectrum was identical to the one obtained upon the addition of only Cu^2+^ ion, indicating that no peptoid duplex was formed. These results suggest that (**Helix HQT *i* + 3**)Cu is both the kinetic and the thermodynamic product in these conditions. We therefore sought to thermodynamically control this transformation by stirring the reaction mixture (peptoid and metal ions) for extended periods of time, but unfortunately, no change in the UV-Vis spectrum was recorded even after 52 h. Only upon stirring the reaction mixture at 50 °C for 2 hours was a change in the solution color from colorless to light pink (indicative of a Terpy–Fe complex) observed, and the UV spectrum showed the disappearance of the bands near *λ* = 259 and 328 nm and the appearance of the bands near *λ* = 266 and 559 nm (Fig. 7D, blue line). These findings suggested that the peptoid duplex (**Helix HQT *i* + 3**)_2_FeCu can be formed under thermodynamic conditions, as was further supported by MS.

### The role of peptoid helicity and ligands' pre-organization in the selective recognition of two different metal ions

Finally, we set up to determine whether the pre-organization of HQ and Terpy within the peptoid **Helix HQT *i* + 3** also affects its unique binding with Co^2+^ or Zn^2+^, which enables the formation of heteronuclear metallopeptoid duplexes at rt. For this purpose, we have tested the ability of the four control peptoids (Fig. 5A) to bind two metal ions, Cu^2+^ and either Co^2+^ or Zn^2+^, using UV-Vis spectroscopy. Starting with the step approach, we have noticed that upon addition of 0.5 equivalent of either Co^2+^ or Zn^2+^ to each of the control peptoids, both Terpy and HQ absorption bands were changed, indicating that the metal ions bind simultaneously to both HQ and Terpy towards the formation of intramolecular complexes (see ESI†). These results demonstrated that unlike **Helix HQT *i* + 3**, the control peptoids are not able to selectively bind Co^2+^ or Zn^2+^ exclusively in the Terpy site; therefore, the step approach cannot be applied for generating (peptoid)_2_CuM complexes. Therefore, we conducted UV-Vis experiments using mixtures of either Cu^2+^ and Zn^2+^ or Co^2+^ and Cu^2+^. Interestingly, in both cases, the addition of a solution containing 0.5 equivalent of each of Cu^2+^ and Zn^2+^ or Cu^2+^ and Co^2+^ to 1 equivalent of each peptoid resulted in a simultaneous but only partial decrease of the absorbance bands corresponding to both Terpy and HQ. Only upon treating these solutions with an additional portion of 0.5 equivalent of each of Cu^2+^ and Zn^2+^ or Cu^2+^ and Co^2+^, a full decrease in the absorbance bands corresponding to both Terpy and HQ was obtained (see ESI†). These results suggest that a mixture of products was generated with each of the control peptoids, most probably two different intramolecular (peptoid)M complexes. Thus, both the helicity and the pre-organization of Terpy and HQ are important factors also in the creation of the heteronuclear peptoid duplexes (peptoid)_2_CuM.

## Conclusions

In this study, we have demonstrated the rational design of a biomimetic oligomer peptoid, **Helix HQT *i* + 3**, which can perform the recognition processes as follows: (1) selective binding of Cu^2+^ ion and its extraction from a solution containing the ions Co^2+^, Zn^2+^, Fe^3+^, Mn^2+^ and Ni^2+^ in high concentrations, resulting in the intramolecular complex (**Helix HQT *i* + 3**)Cu; (2) selective binding of two different metal ions, each at a specific metal-binding site, from a mixture containing both ions, to generate the intermolecular bimetallic duplexes (**Helix HQT *i* + 3**)CuM (M = Co^2+^, Zn^2+^, and Fe^3+^); and (3) selective transition from an intermolecular to an intramolecular binding as the metal-binding peptoid adjusts its coordination properties to changes in its environment (*i.e.* excess of one metal ion over another one). The high selectivity in all these cases reflects the unique ability of this peptoid to mimic the recognition properties of metal-binding biopolymers. These properties, together with our current efforts to increase the water solubility of this peptoid and to design similar peptoids for selective binding of Zn^2+^, might enable various applications such as chelate therapy and selective catalysis.^21^

## Experimental

### Materials

Rink amide resin was purchased from Novabiochem; trifluoroacetic acid (TFA), zinc acetate dehydrate and nickel acetate tetrahydrate were purchased from Alfa Aesar; 8-hydroxy-2-quinolinecarbonitrile, (*S*)-(–)-1-phenylethylamine (*N*spe), 4′-chloro-2,2′:6′,2′′-terpyridine and manganese acetate tetrahydrate were purchased from Acros; bromoacetic acid, cobalt acetate tetrahydrate and copper acetate monohydrate were purchased from MERCK; *N*,*N*′-diisopropylcarbodiimide (DIC), piperidine, 2-methoxyethylamine, benzylamine, acetonitrile (ACN) and water and HPLC grade solvents were purchased from Sigma-Aldrich; iron perchlorate hydrate was purchased from Strem Chemicals; dimethylformamide (DMF) and methanol (MeOH) solvents were purchased from Bio-Lab Ltd. These reagents and solvents were used without additional purification. 2,2′:6′,2′′-Terpyridine amine (*N*terpy) and 8-hydroxy-2-quinolinemethylamine (*N*hq) were synthesized according to previously published procedures.^13^

### Synthesis and purification of the peptoid oligomers

Peptoid oligomers were synthesized manually at room temperature on Rink amide resin using a variation of a previously reported peptoid sub-monomer protocol.^13^ Peptoid synthesis was carried out with alternating bromoacylation and amine displacement steps until peptoid oligomers of the desired sequence were obtained. In our case, after incorporation of 8-hydroxy-2-quinolinemethylamine, 0.17 mL of a 1.2 M solution of bromoacetic acid, 0.04 mL of neat *N*,*N*′-diisopropylcarbodiimide (DIC) and 0.29 mL of DMF were added to the resin and mixed at room temperature for 20 minutes.^13^ When the desired sequence was achieved, the peptoid products were cleaved from the resin by treatment with 95% trifluoroacetic acid (TFA) in water (50 mL g^–1^ resin) for 30 minutes. After filtration, the cleavage mixture was concentrated by rotary evaporation under reduced pressure. Cleaved samples were then re-suspended in 50% acetonitrile in water and lyophilized to powders. Peptoids were purified by preparative High Performance Liquid Chromatography (HPLC) using a C18 column. Products were detected by UV absorbance at 230 nm during a linear gradient conducted from 5% to 95% solvent B (0.1% TFA in HPLC grade acetonitrile) over solvent A (0.1% TFA in HPLC grade water) in 50 minutes with a flow rate of 5 mL min^–1^. Purified peptoid oligomers were analyzed by reversed-phase HPLC (C18 column) with a linear gradient of 5–95% ACN in water (0.1% TFA) over 10 min at a flow rate of 700 μL min^–1^ and 214 nm of UV absorbance. Mass spectrometry of peptoid oligomers was performed on Waters LCT Premier mass spectrometer under electrospray ionization (ESI), direct probe ACN : H_2_O (70 : 30), flow rate 0.3 mL min^–1^ or on Advion expression CMS mass spectrometer under electrospray ionization (ESI), direct probe ACN : H_2_O (95 : 5), flow rate 0.2 mL min^–1^.

### UV-Vis spectroscopy

Titration experiments of the peptoid **Helix HQT *i* + 3** with the metal ions (Co^2+^, Cu^2+^, Ni^2+^, Mn^2+^, Zn^2+^, and Fe^3+^) were followed by UV-Vis in two different solvents. In a typical experiment, 10–15 μL of a peptoid solution (5 mM in MeOH or ACN) was diluted in 3 mL 4 : 1 MeOH : H_2_O or acetonitrile solution (to get 17–26 μM concentration) and then sequentially titrated with 2 μL aliquots of a metal ion (2.5 or 5 mM in H_2_O), in multiple steps, until the binding was completed. In the selectivity experiments, solutions containing mixtures of metal ions (1 equivalent of Cu^2+^ and 1–20 equivalents of Co^2+^, Zn^2+^, Mn^2+^, Ni^2+^ and Fe^3+^, which is of 10 μL of 5 mM and 2–40 μL of 25 mM, respectively) in 3 mL of 4 : 1 MeOH : H_2_O or acetonitrile were first measured as a blank. Then, peptoid **Helix HQT *i* + 3** was added (10 μL, 5 mM) and the spectrum was measured again. UV measurements were performed using an Agilent Cary 60 UV-Vis spectrophotometer, a double beam, Czerny–Turner monochromator.

### Synthesis of metal complexes for MS analysis

Samples for MS analysis were prepared shortly before measurements. In a typical experiment, a solution of peptoid oligomers (100–200 μL of 0.05 mM) in MeOH or ACN was treated with metal solution (5 mM in H_2_O or ACN) and the mixture was stirred for 30 minutes prior to MS analysis. Mass spectrometry analysis of the metal complexes was performed on a Waters LCT Premier mass spectrometer under electrospray ionization (ESI), direct probe ACN : H_2_O (70 : 30), flow rate 0.3 mL min^–1^.

### Circular dichroism spectroscopy

Approximately, 500 μL solutions (5 mM in methanol or ACN) of lyophilized peptoids powders were prepared immediately before CD measurements. CD scans were performed at 25 °C at a concentration of 100 μM in a solution of methanol/water 4 : 1 or ACN. The spectra were obtained by averaging 4 scans per sample in a fused quartz cell (path length = 0.1 cm). Scans were performed over the 320 to 190 nm region using 50 nm min^–1^ scan rate. CD measurements were performed using a circular dichroism spectrometer Model Jasco 810 Spectropolarimeter and Applied Photophysics Chirascan.

### Synthesis of Cu(ii) complexes for EPR analysis

Copper complexes for EPR were prepared in methanol (0.4 mL) by addition of 1.2 equivalents of copper acetate to **Helix HQT *i* + 3** (6.4 mg, 0.0053 mmol), **Helix HQT *i* + 4** (6.1 mg, 0.005 mmol), **Helix HQT *i* + 2** (4.2 mg, 0.0035 mmol), **Nonhelix HQT *i* + 3** (5.8 mg, 0.0053 mmol) and **DI HQT** (4 mg, 0.007 mmol) and stirring the solution for 1 hour. A green solid was precipitated after the addition of NH_4_PF_6_ (0.08 mL of 1 M aqueous solution). The precipitates were isolated by centrifugation, washed twice with water and lyophilized overnight. (**Helix *i* + 3**)Cu was obtained in 76% yield (5.1 mg), (**Helix HQT *i* + 4**)Cu 79% yield (5.0 mg), (**Helix HQT *i* + 2**)Cu 81% yield (3.6 mg), (**Nonhelix HQT *i* + 3**)Cu 80% (4.9 mg), **DI HQT**-Cu 70% yield (3.1 mg). (**Helix HQT *i* + 3**)_2_ZnCu complex was synthesized by addition of 0.5 equivalents of Zn acetate in ACN (5 mM) to the solution of **Helix HQT *i* + 3** in ACN (5 mg, 21 mM). The solution was stirred for 10 min and 0.5 equivalents of Cu in ACN (5 mM) was added and the mixture was stirred for 30 min. Then, 500 μL of water was added, the solvent was lyophilized overnight, the complex was washed twice with water and dried by lyophilization. Mass of the complex = 4.02 mg, yield: 72%. EPR spectra were obtained on a Bruker EMX-10/12 X-band (*ν* = 9.4 GHz) digital EPR spectrometer. All spectra of peptoid copper complexes were obtained at room temperature from solid state with (2,2,6,6-tetramethyl-1-piperidinyl)oxidanyl (TEMPO, *g* = 2.0059) in an inner tube for determination of the *g*-factor. Spectra processing and simulation were performed with a Bruker WIN-EPR and SimFonia Software.

### Inductively coupled plasma (ICP) experiments

To a solution of 1 equivalents of Cu^2+^ (0.5 mM in MeOH : H_2_O 4 : 1) and 10 equivalents of Co^2+^, Mn^2+^, Ni^2+^, Fe^3+^ and Zn^2+^ (5 mM each in MeOH : H_2_O 4 : 1), 6 mg of peptoid **Helix HQT *i* + 3** were added and the mixture was allowed to shake for 30 min. The solvent was then evaporated and the water solution was lyophilized overnight. To the lyophilized powder, 1 mL of H_2_O was added; the mixture was shaken for 5 min and centrifuged 15 min to separate the solution and the precipitate. After the centrifugation, the water was removed and this process was repeated another 9 times. To the precipitated metallopeptoid, 0.2 mL of 69% nitric acid HNO_3_ was added, the mixture was mixed thoroughly for 30 minutes, followed by the addition of water to get 10 mL final volume. This experiment was done 3 times. When ACN was used as a solvent, 1 equivalent each of Cu^2+^, Co^2+^, Mn^2+^, Ni^2+^, Fe^3+^ and Zn^2+^ was added (1 mM each in ACN : H_2_O 17 : 3) to 5.6 mg of peptoid **Helix HQT *i* + 3**. All solutions were filtered by 0.2 μm filters prior to ICP analysis by a Thermo Scientific iCAP 6000 ICP-OES analyzer. Wavelengths used for the detection of the different metal ions were as follows: 324.7 nm for Cu, 228.6 nm for Co, 257.6 nm for Mn, 231.6 nm for Ni, 213.8 nm for Zn.

### Binding constants calculations

The association constants for metal binding were measured using UV-Vis spectroscopy by titration of 2 μL aliquots of a metal ion solution (2 mM in H_2_O) into a 3 mL solution of the peptoid (typically 6–8 μM) in MeOH : H_2_O 1 : 5. The binding was followed by obtaining the UV-Vis spectrum from 200 to 500 nm as a function of the total added metal ions.

The metal binding to the reported peptoids can be described by the equilibrium as follows:
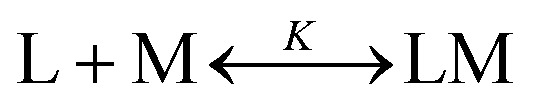


Defining [L] = [L_0_] – [LM]
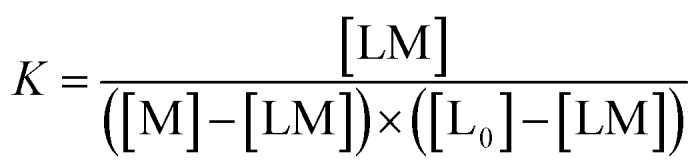


The real solution to [LM] is1
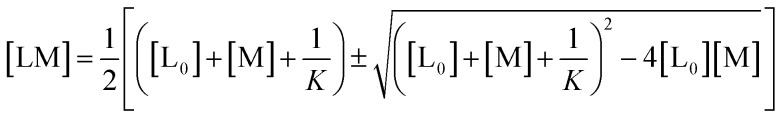



Now,2
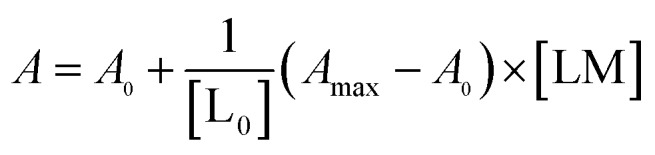
where *A*_0_ and *A*_max_ are the minimum and maximum absorbance measured for the free ligand, respectively, and L_0_ is the initial concentration of the free ligand.

Substitution of [LM] in eqn (2) with the expression in eqn (1), gives3
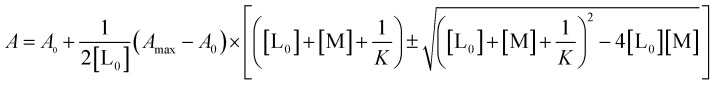



The results were fitted by a nonlinear regression (curve fit)5*b* using GraphPad Prism® software.

## Supplementary Material

Supplementary information
