# Supplementary material for: A rationally designed metal-binding helical peptoid for selective recognition processes
Source: Chem Sci. 2016 Jan 8;7(4):2809–20. doi: 10.1039/c5sc04358a (PMC5477017; doi:10.1039/c5sc04358a)
Supplement: Supplementary file 1 [file SC-007-C5SC04358A-s001.pdf]

Electronic Supplementary Material (ESI) for Chemical Science.
This journal is © The Royal Society of Chemistry 2016

A Rationally Designed Metal-Binding Helical Peptoid for Selective Recognition Processes

Maria Baskin and Galia Maayan

Schulich Faculty of Chemistry, Technion- Israel Institute of Technology, Haifa, Israel, 32000

Supporting Information

Table of Contents

1. Instrumentation……………………………………………...……………………….... 2
2. Synthetic scheme of *N*hq and *N*terpy…………………………………………….……..……2
3. HPLC data…………………………………………..……………………………..….…3-5
4. CD data………………………………………………………………………………….5-6
5. UV-vis data……………………………………………...…………………..…7-30

   13.1 UV-Vis of **Helix HQT i+3** and its metal complexes in MeOH:H_2O…..……..…7-13

   13.2 UV-Vis of control peptoids with mixture of metal ions in MeOH:H_2O……..…13-15

   13.3 UV-Vis of **Helix HQT i+3** and its metal complexes in ACN…..………………15-22

   13.4 UV-Vis of control peptoids and their metal complexes in ACN………..……22-30

6. Association constants…………………………………………………………...30-33
7. EPR data…………………………………………………………..…..……..……34
8. ESI-MS data………………………………………………………………..……35-59

Instrumentation:

Peptoid oligomers were analyzed by reversed-phase HPLC (analytical C18(2) column, Phenomenex, Luna 5μm, 100 Å, 2.0x50 mm) on a Jasco UV-2075 PLUS detector. A linear gradient of 5–95% ACN in water (0.1% TFA) over 10 min was used at a flow rate of 700 μL/min. Preparative HPLC was performed using a AXIA Packed C18(2) column (Phenomenex, Luna 15μm, 100 Å, 21.20x100mm). Peaks were eluted with a linear gradient of 5–95% ACN in water (0.1% TFA) over 50 min at a flow rate of 5 mL/min. Mass spectrometry of peptoid oligomers and their metal complexes was performed on a Advion expression CMS mass spectrometer under electrospray ionization (ESI), direct probe ACN:H_2O (95:5), flow rate 0.2 ml/min and on a Waters LCT Premier mass spectrometer under electrospray ionization (ESI), direct probe ACN:H_2O (70:30), flow rate 0.3 ml/min. UV measurements were performed using an Agilent Cary 60 UV-Vis spectrophotometer, a double beam, Czerny-Turner monochromator. CD measurements were performed using a circular dichroism spectrometer Model Jasco 810 Spectropolarimeter and AppliedPhotophysics Chirascan. ICP measurements were performed using Thermo Scientific iCAP 6000 ICP-OES analyzer. EPR spectra were recorded on a Bruker EMX-10/12 X-band (ν=9.4 GHz) digital EPR Spectrometer. Spectra processing and simulation were performed with Bruker WIN-EPR and SimFonia Software. Data processing was done with the softwares Excel and KaleidaGraph.

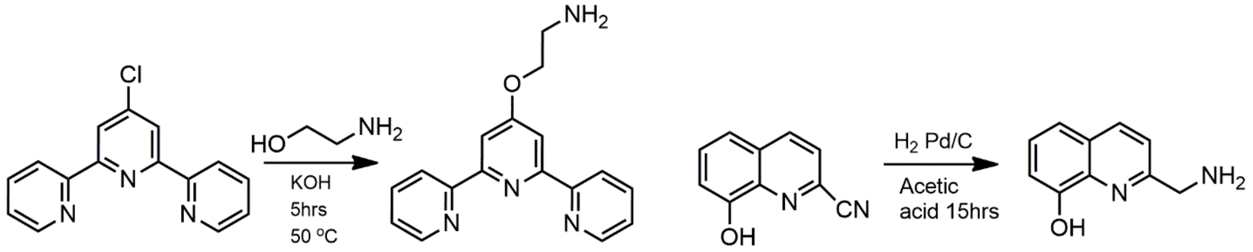

Scheme 1: Synthetic routs to the primary amines *N*terpy and *N*hq.

HPLC

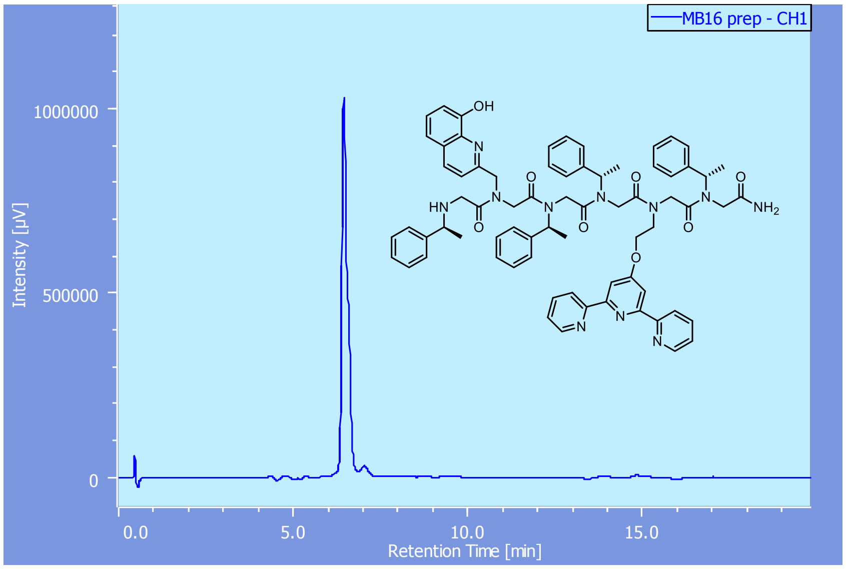

Figure S1. HPLC traces of purified peptoid oligomer **Helix HQT i+3** at 214nm.

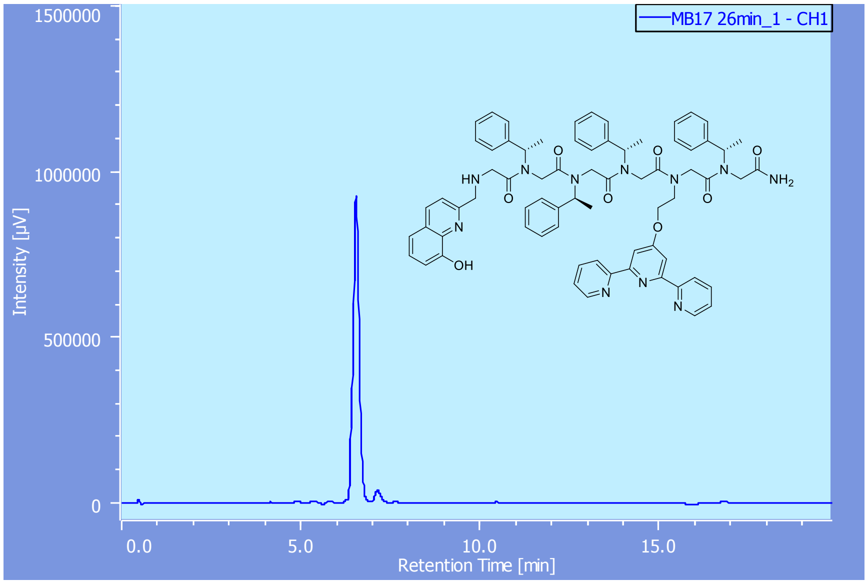

Figure S2. HPLC traces of purified peptoid oligomer **Helix HQT i+4** at 214nm.

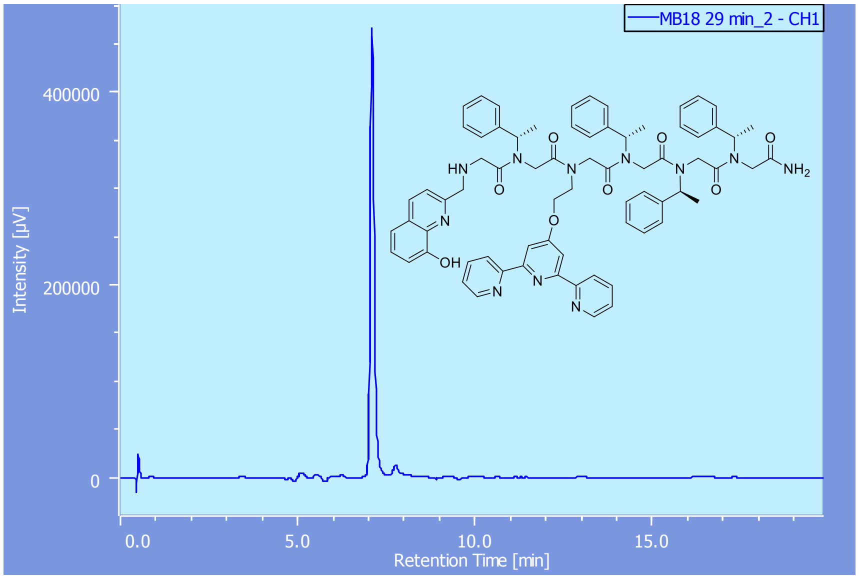

Figure S3. HPLC traces of purified peptoid oligomer **Helix HQT i+2** at 214nm.

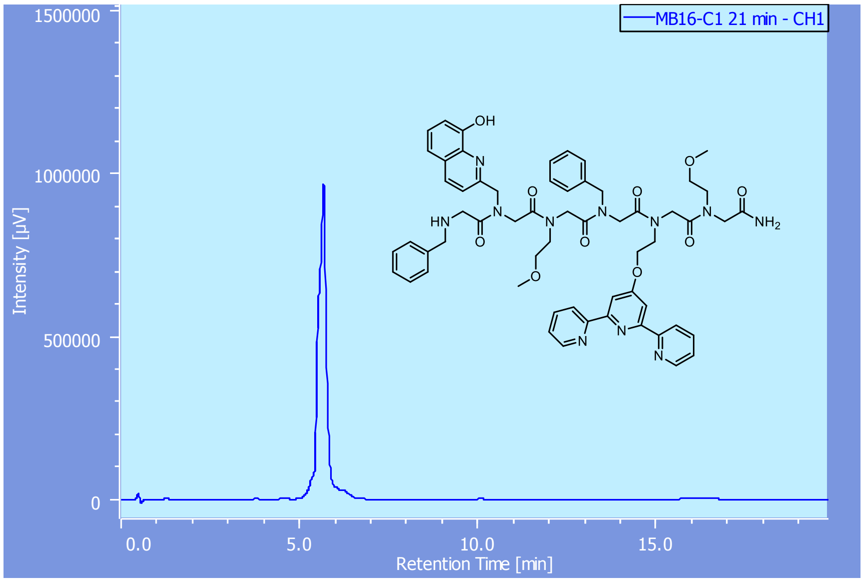

Figure S4. HPLC traces of purified peptoid oligomer **Nonhelix HQT i+3** at 214nm.

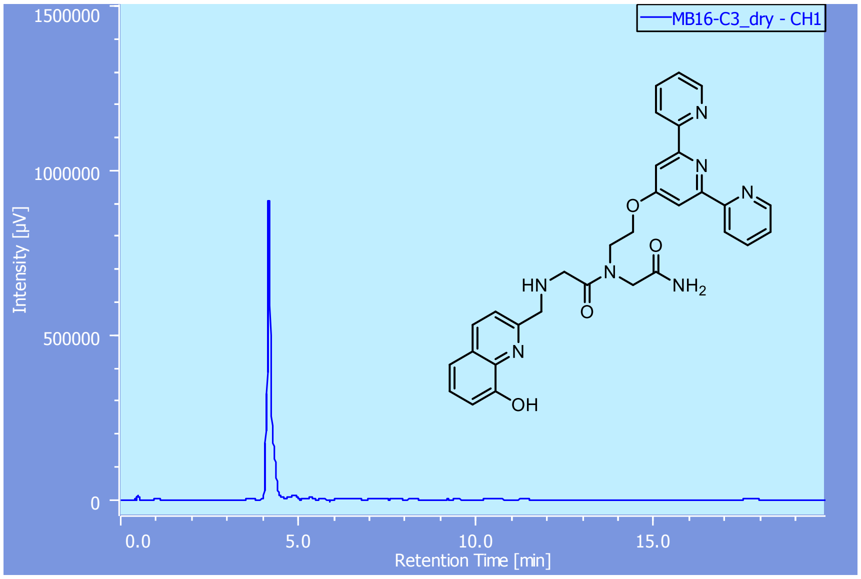

Figure S5. HPLC traces of purified peptoid oligomer **DI HQT** at 214nm.

CD data

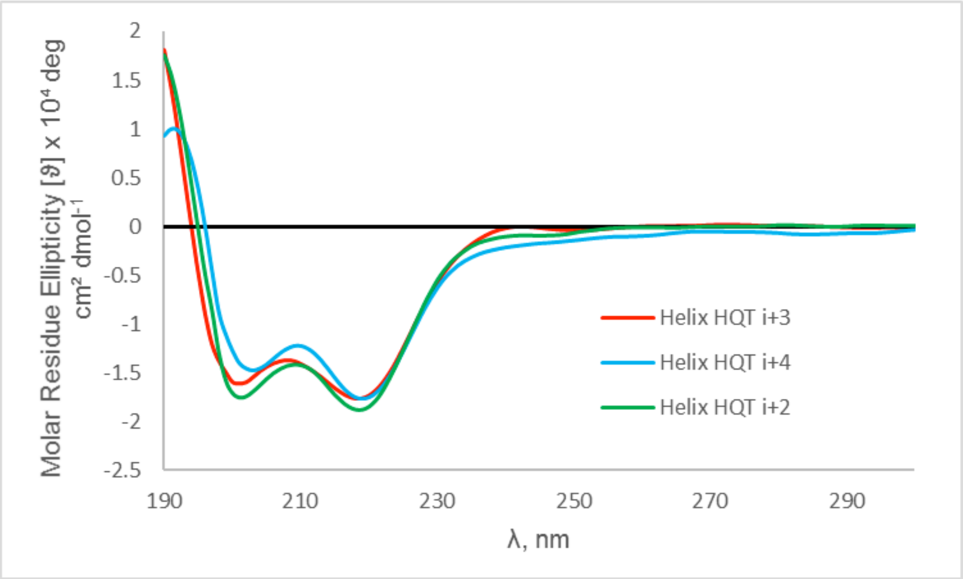

Figure S6. CD spectra of peptoid oligomers **Helix HQT i+3**, **Helix HQT i+4 and Helix HQT i+2**. The spectra were recorded at room temperature, at concentrations of 100 µM in MeOH:H_2O 4:1.

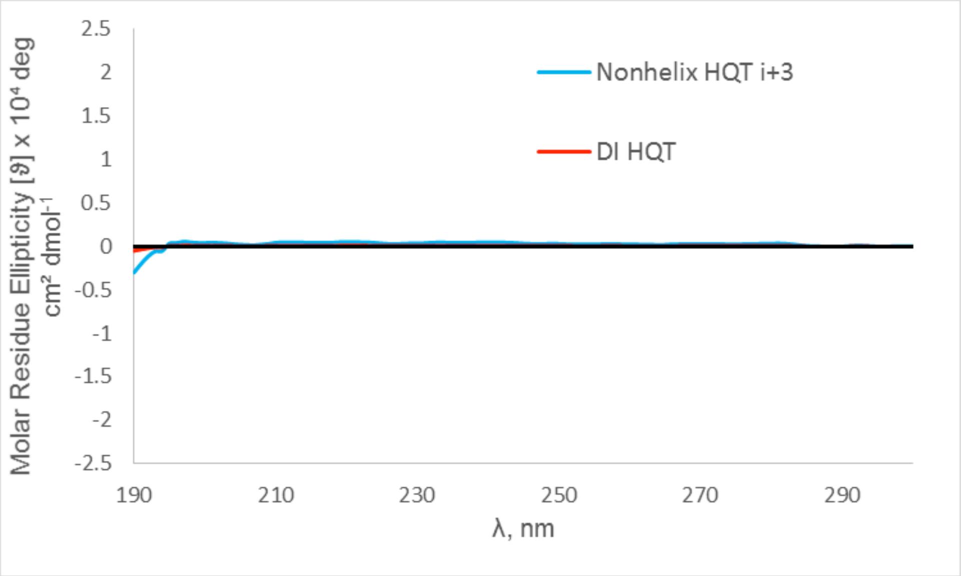

Figure S7. CD spectra of peptoid oligomers **Nonhelix HQT i+3** and **DI HQT**. The spectra were recorded at room temperature, at concentrations of 100 μM in MeOH:H_2O 4:1.

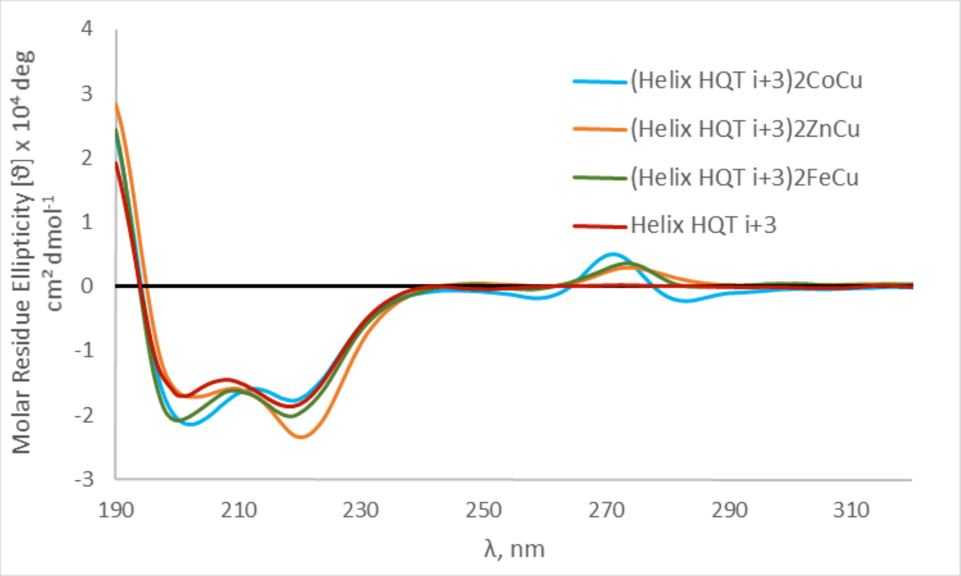

Figure S8. CD spectra of hetero bimetallic complexes (**Helix HQT i+3**)$_2$CoCu, (**Helix HQT i+3**)$_2$ZnCu and (**Helix HQT i+3**)$_2$FeCu. The spectra were recorded at room temperature, at concentrations of 100 μM in ACN.

UV-Vis data

UV-Vis of **Helix HQT i+3** and its metal complexes in MeOH/H_2O

Table S1. UV-Vis absorbance data of peptoid oligomer **Helix HQT i+3** and it metal complexes in MeOH/H_2O 4:1.

(**Helix HQT i+3**)M complex	λmax (nm)	Figure #
Co^{2+}	262, 309	S9
Zn^{2+}	265, 310, 322	S10
Mn^{2+}	266, 311, 322	S11
Ni^{2+}	268, 312, 325	S12
Fe^{3+}	249, 316, 556	S13

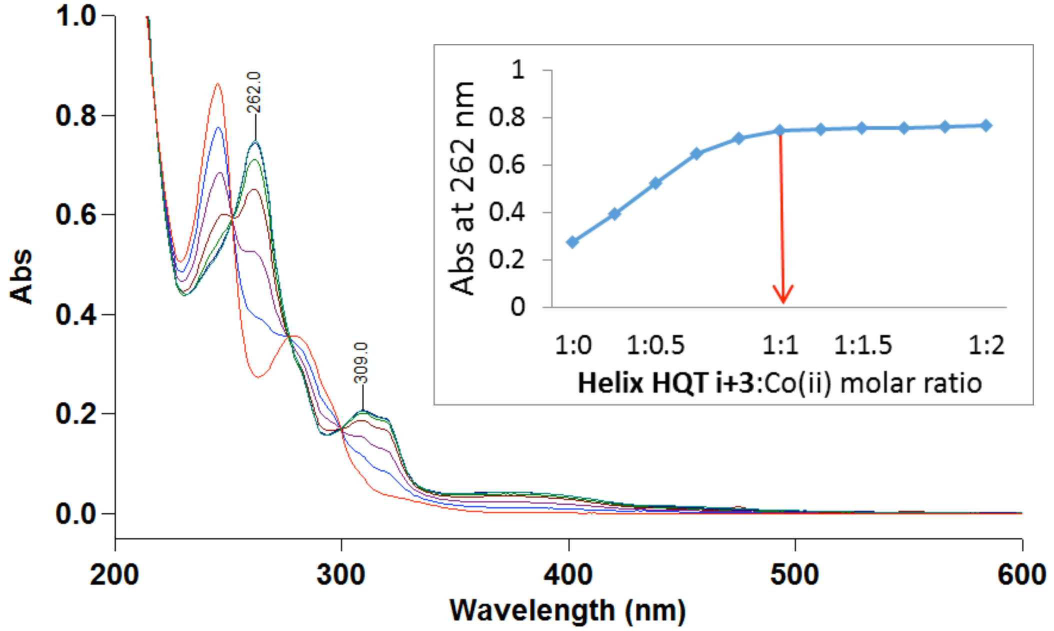

Figure S9. UV-Vis titration and a peptoid-to-metal ratio plot of **Helix HQT i+3** (17 µM) with Co^{2+} acetate (5mM) in 3 ml MeOH:H_2O 4:1.

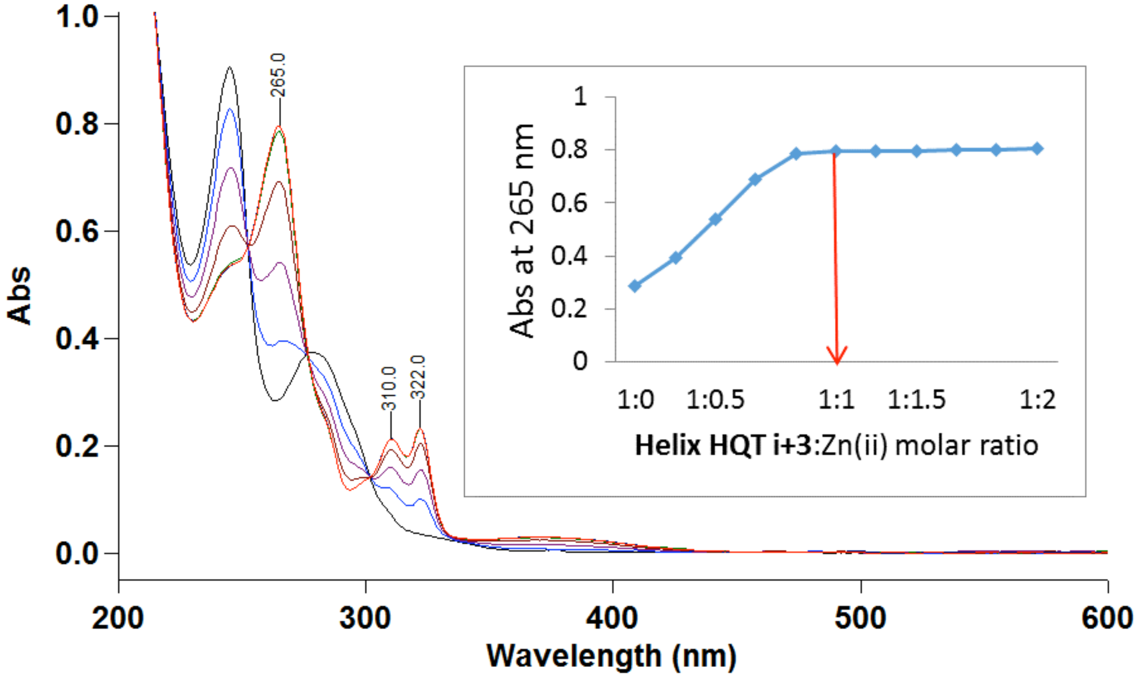

Figure S10. UV-Vis titration and a peptoid-to-metal ratio plot of **Helix HQT i+3** (17 µM) with Zn^{2+} acetate (5mM) in 3ml MeOH:H_2O 4:1.

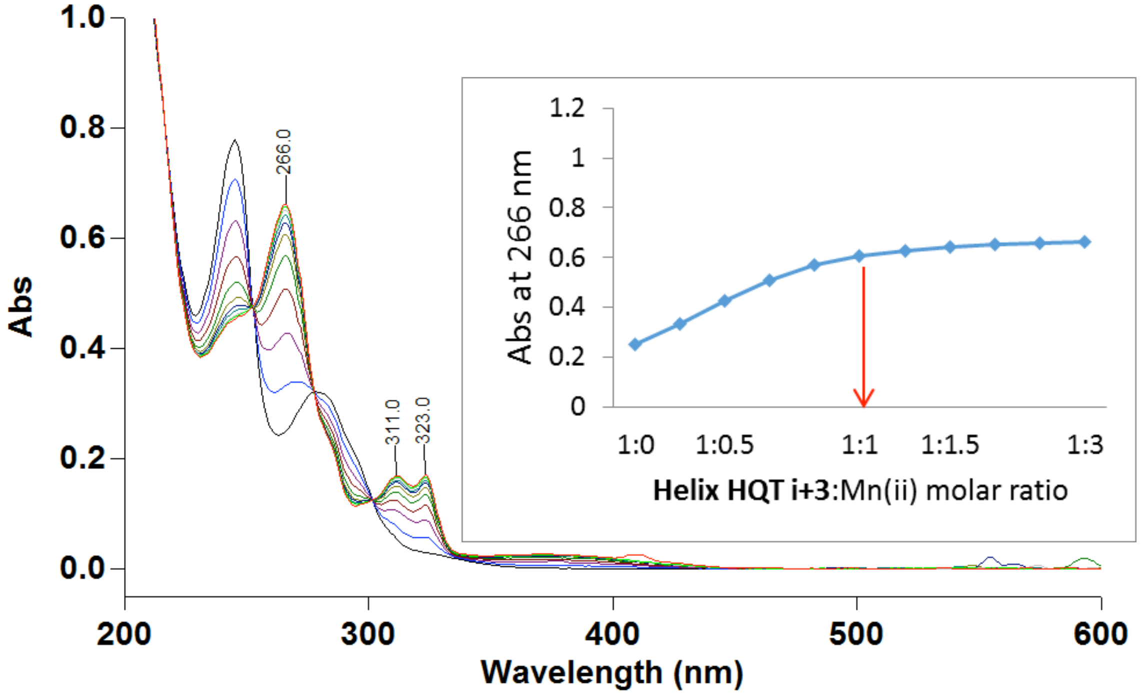

Figure S11. UV-Vis titration and a peptoid-to-metal ratio plot of **Helix HQT i+3** (17 µM) with Mn^{2+} acetate (5mM) in 3ml MeOH:H_2O 4:1.

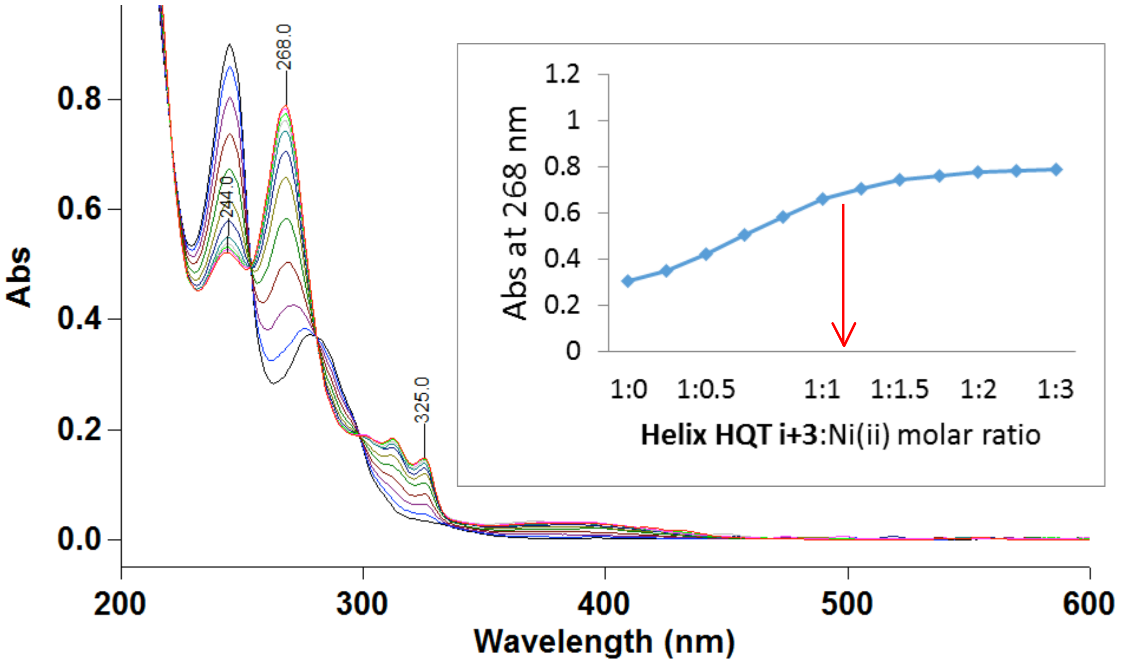

Figure S12. UV-Vis titration and a peptoid-to-metal ratio plot of **Helix HQT i+3** (17 µM) with Ni^{2+} acetate (5mM) in 3ml MeOH:H_2O 4:1.

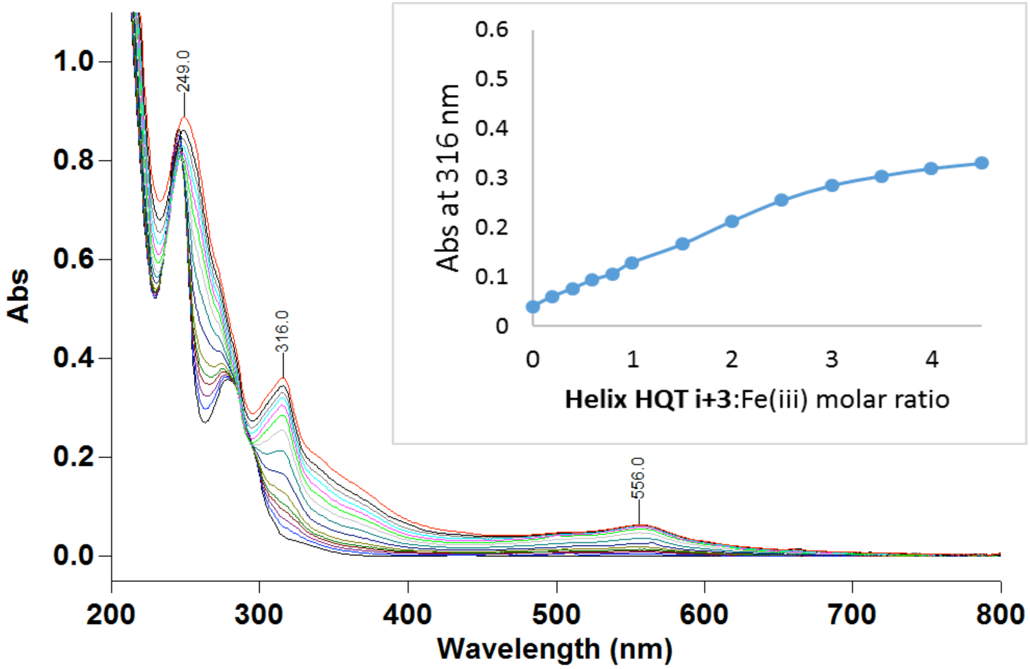

Figure S13. UV-Vis titration and a peptoid-to-metal ratio plot of **Helix HQT i+3** (17 µM) with Ni^{2+} acetate (5mM) in 3ml MeOH:H_2O 4:1. The peptoid-to-metal ratio plot in this case does not provide information about the binding.

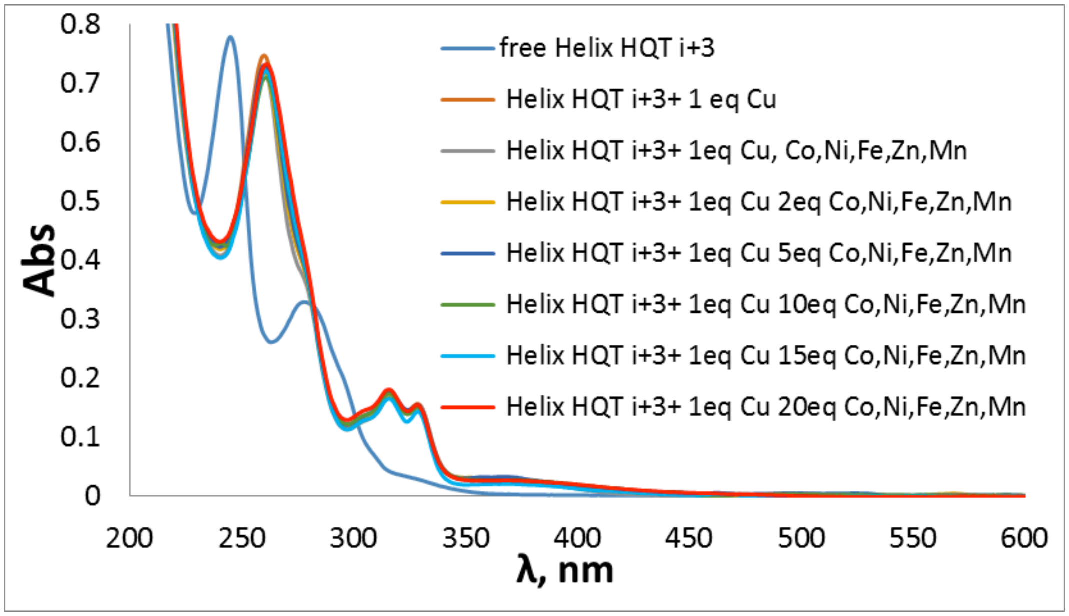

Figure S14. UV of **Helix HQT i+3** (17 µM) with 1 equiv. Cu^{2+} and 1-20 equiv. of Co^{2+}, Ni^{2+}, Fe^{3+},Zn^{2+}, Mn^{2+} in MeOH:H_2O 4:1. Absorption bands off all spectra were 259, 316, 328 nm.

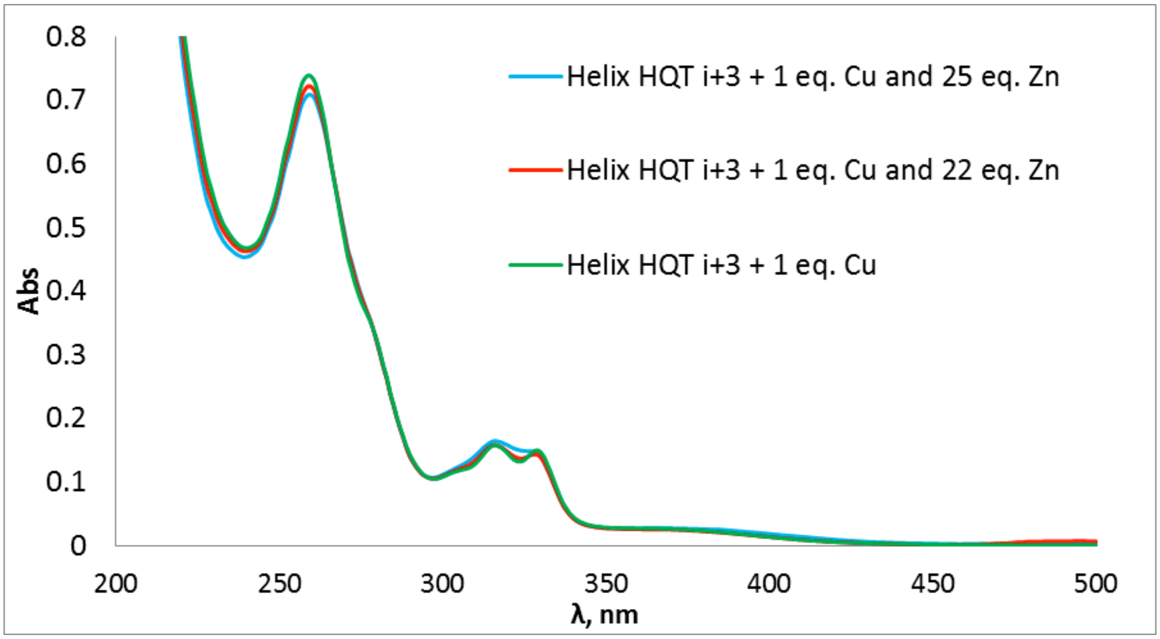

Figure S15. UV-VIs of **Helix HQT i+3** (17 µM) in 5:1 H_2O:MeOH solution containing Cu^{2+} only or mix of 1 equiv. Cu^{2+} and 25 or 22 equiv. Zn^{2+}.

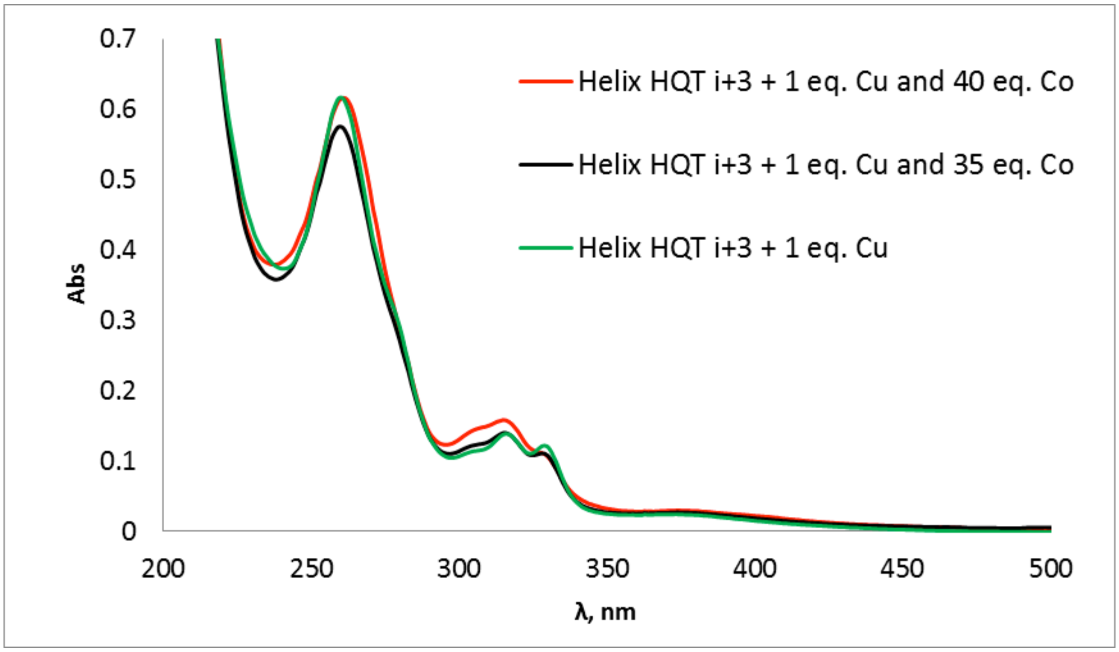

Figure S16. UV-Vis of **Helix HQT i+3** (17 µM) in 5:1 H_2O:MeOH solution containing 1 equiv. Cu^{2+} only or mix of 1 equiv. Cu^{2+} and 35 or 40 equiv. of Co^{2+}.

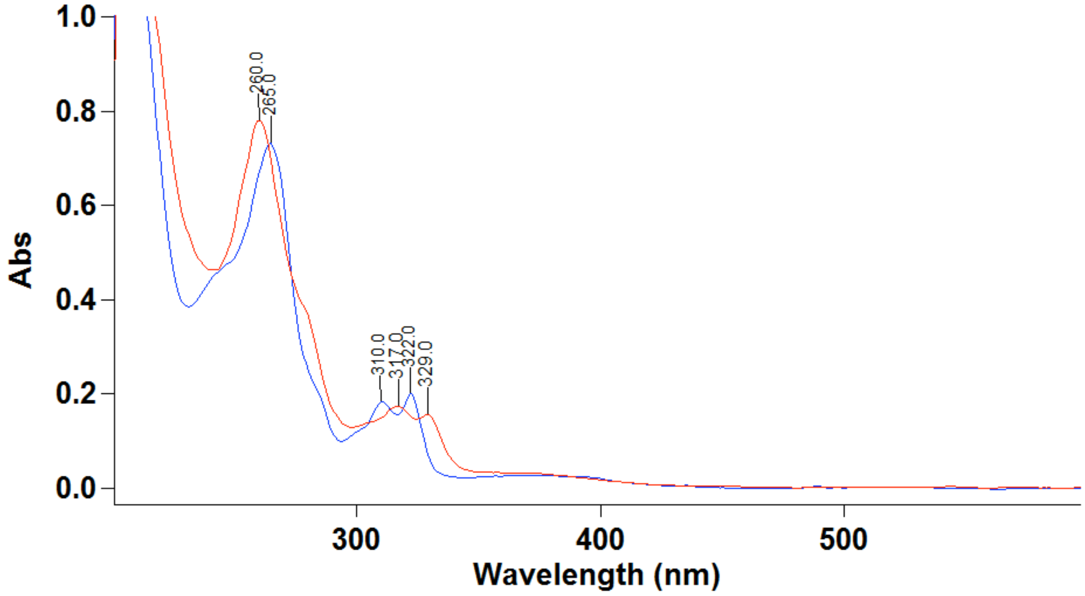

Figure S17. UV-Vis of (**Helix HQT i+3**)Zn (blue, 17 µM) followed by addition of 1 equiv. of Cu^{+2} (red) in MeOH/H_2O 4:1.

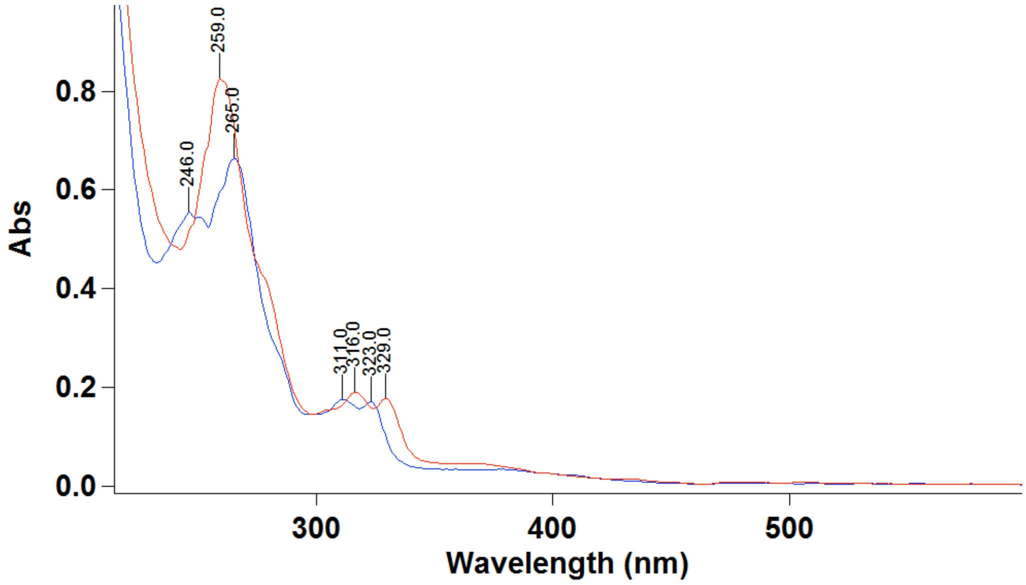

Figure S18. UV-Vis of (**Helix HQT i+3**)Mn (blue, 17 µM) followed by addition of 1 equiv. of Cu^{+2} (red) in MeOH/H_2O 4:1.

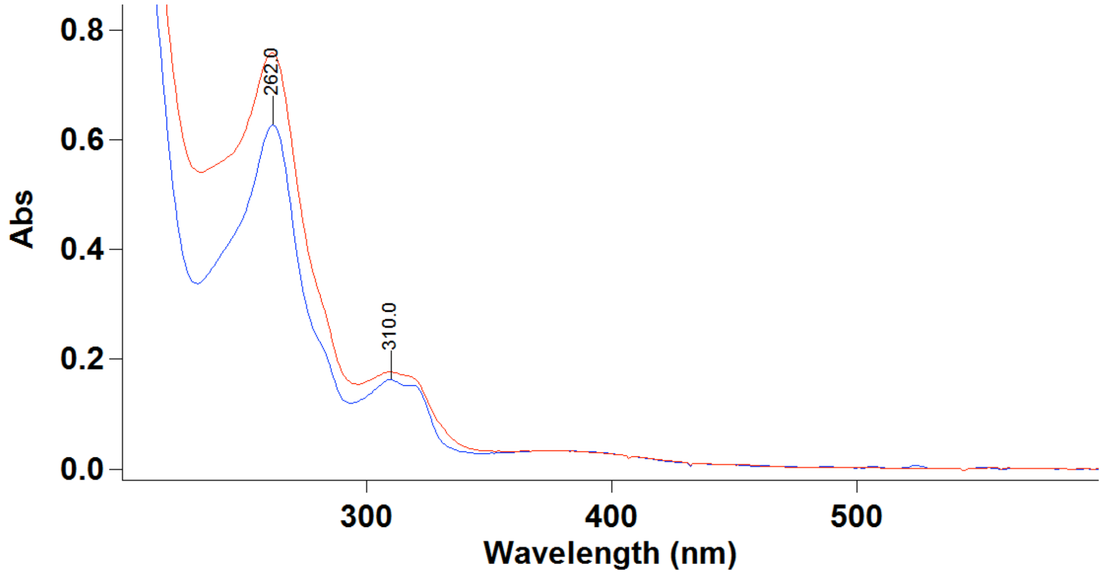

Figure S19. UV-Vis of (**Helix HQT i+3**)Co (blue, 17 µM) followed by addition of 1 equiv. of Cu^{+2} (red) in MeOH/H_2O 4:1.

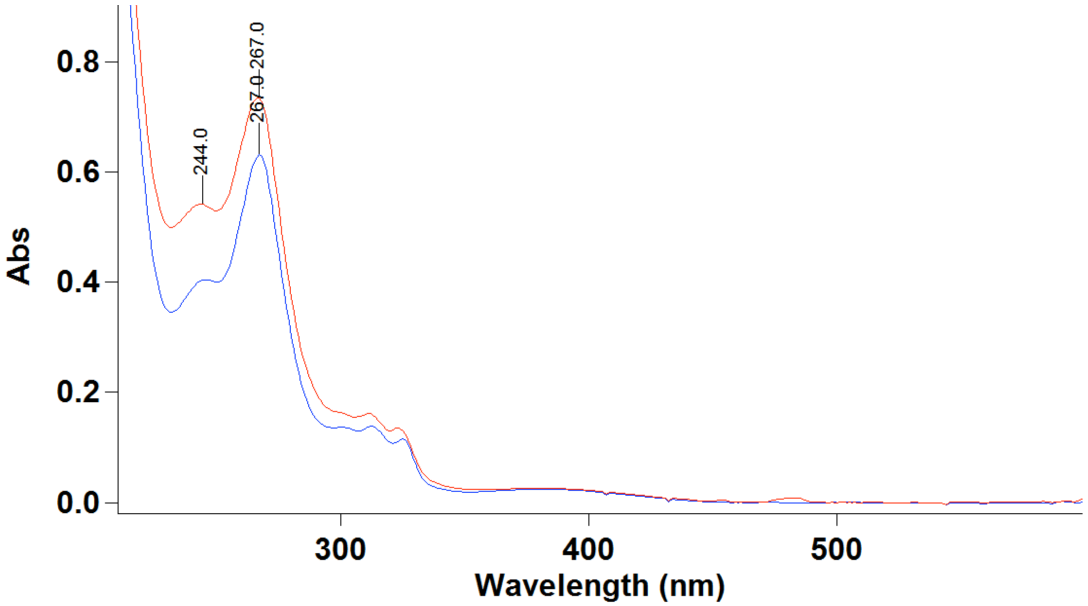

Figure S20. UV-Vis of (**Helix HQT i+3**)Ni (blue, 17 μM) followed by addition of 1 equiv. of Cu^{+2} (red) in MeOH/H_2O 4:1.

UV-Vis of control peptoids with mixture of metal ions in MeOH:H_2O

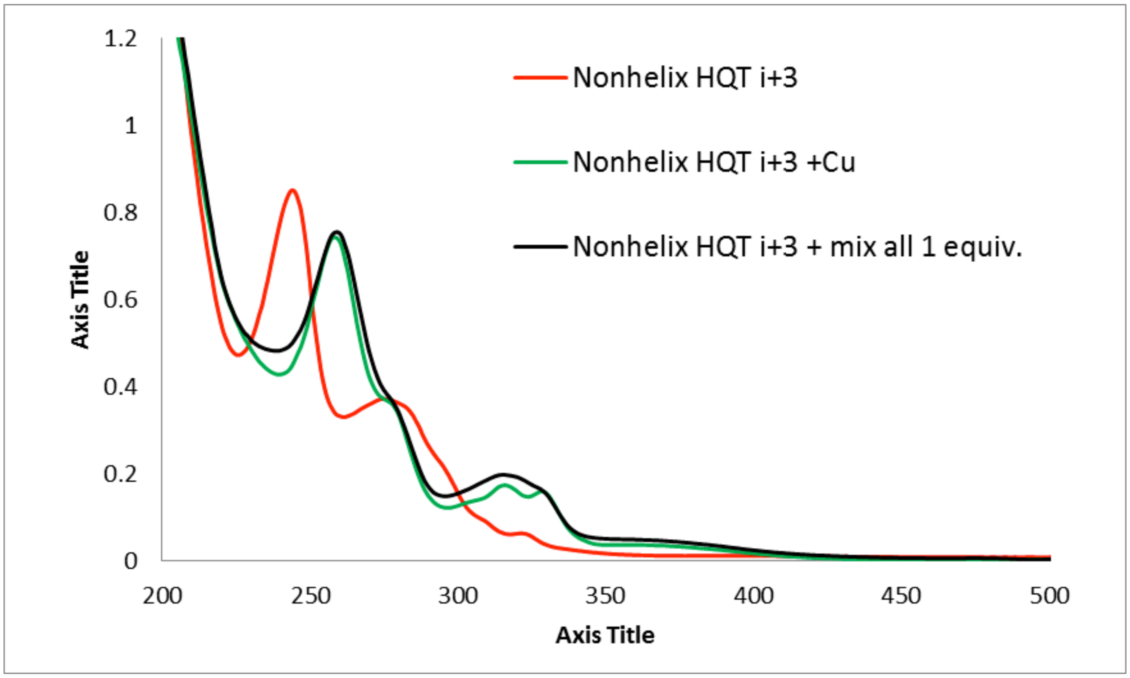

Figure S21. UV-Vis of **Nonhelix HQT i+3** (17 μM) with 1 equiv. Cu^{2+} and 1 equiv. of Cu^{2+} ,Co^{2+}, Ni^{2+}, Fe^{3+}, Zn^{2+}, Mn^{2+} in 3ml MeOH:H_2O 4:1.

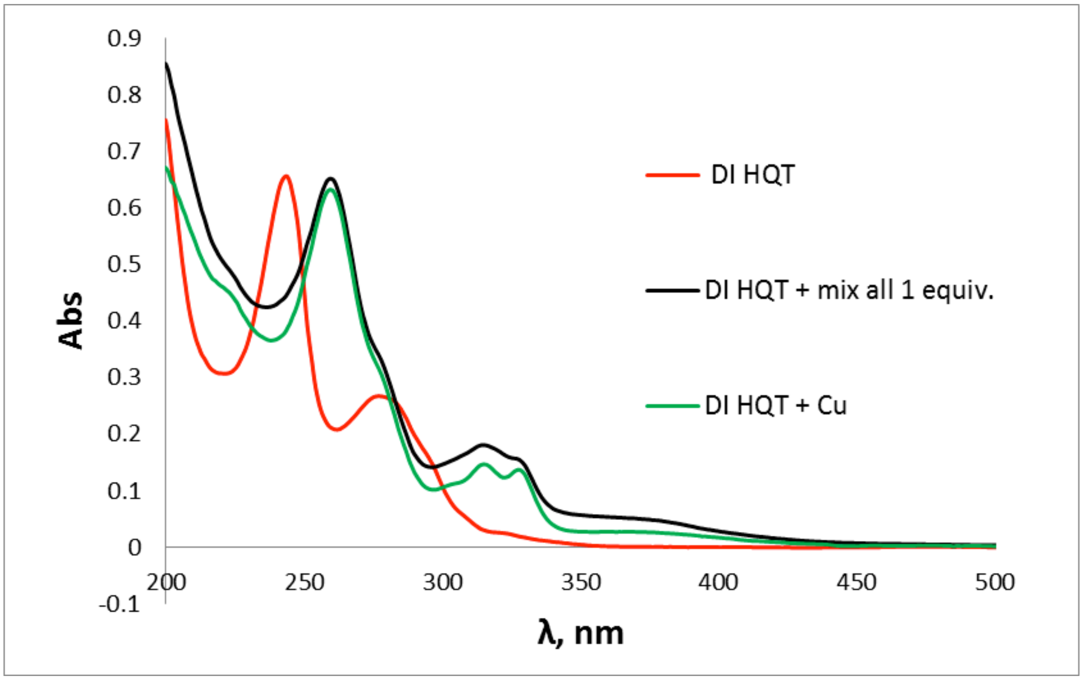

Figure S22. UV-Vis of **DI HQT** (17 µM) with 1 equiv. Cu^{2+} and 1 equiv. of Cu^{2+} ,Co^{2+}, Ni^{2+}, Fe^{3+}, Zn^{2+}, Mn^{2+} in 3ml MeOH:H_2O 4:1.

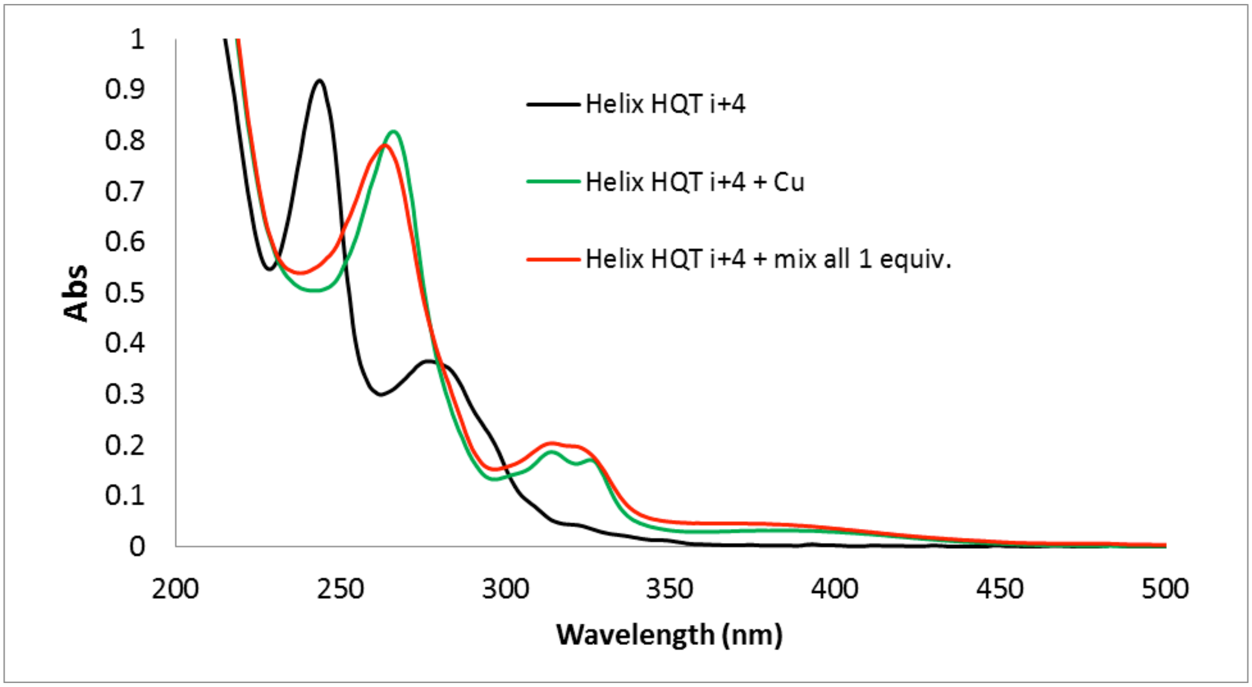

Figure S23. UV-Vis of **Helix HQT i+4** (17 µM) with 1 equiv. Cu^{2+} and 1 equiv. of Cu^{2+} ,Co^{2+}, Ni^{2+}, Fe^{3+}, Zn^{2+}, Mn^{2+} in 3ml MeOH:H_2O 4:1.

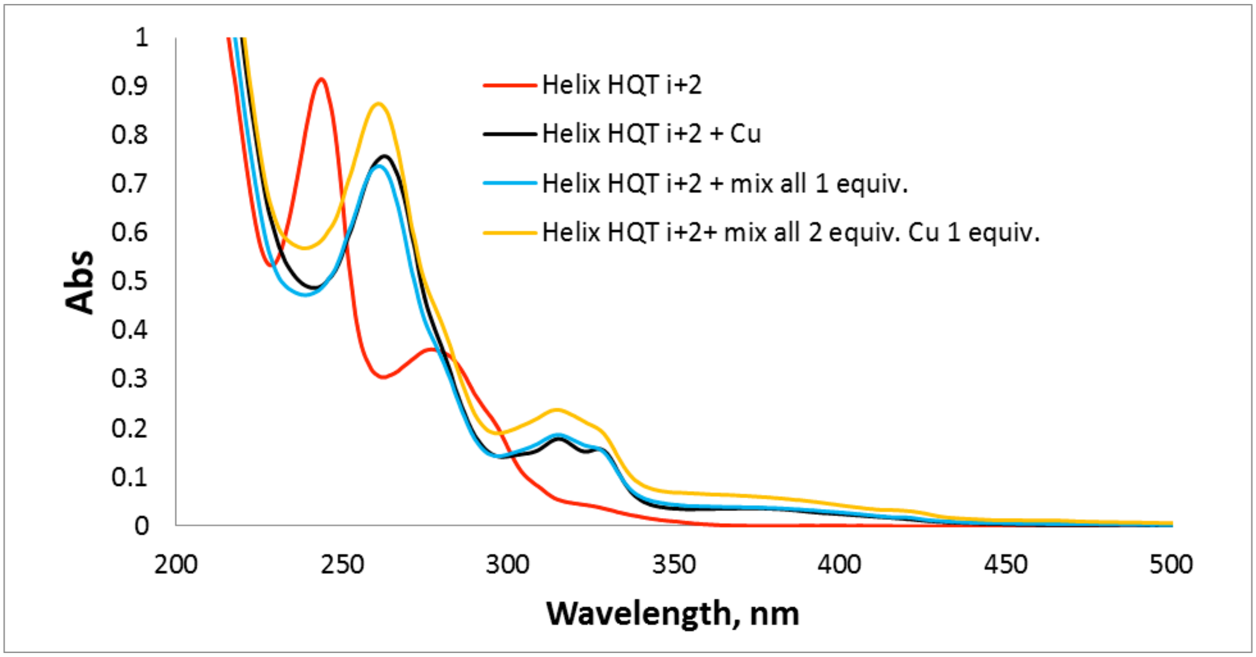

Figure S24. UV-Vis of **Helix HQT i+2** (17 µM) with 1 equiv. Cu^{2+} and 1 and 2 equiv. of Cu^{2+} ,Co^{2+}, Ni^{2+}, Fe^{3+}, Zn^{2+}, Mn^{2+} in 3ml MeOH:H_2O 4:1.

UV-Vis of **Helix HQT i+3** and its metal complexes in ACN

Table S2. Absorbance data of peptoid oligomer **Helix HQT i+3** and it metal complexes in ACN.

(**Helix HQT i**+3)M complex	λmax (nm)	Figure #
Cu^{2+}	263, 316, 329	S27
Co^{2+}	264, 308	S28
Zn^{2+}	268, 311, 323	S29
Fe^{3+}	253, 315, 560	S30
Ni^{2+}	270, 312, 325	S31
Mn^{2+}	263, 312, 323	S32

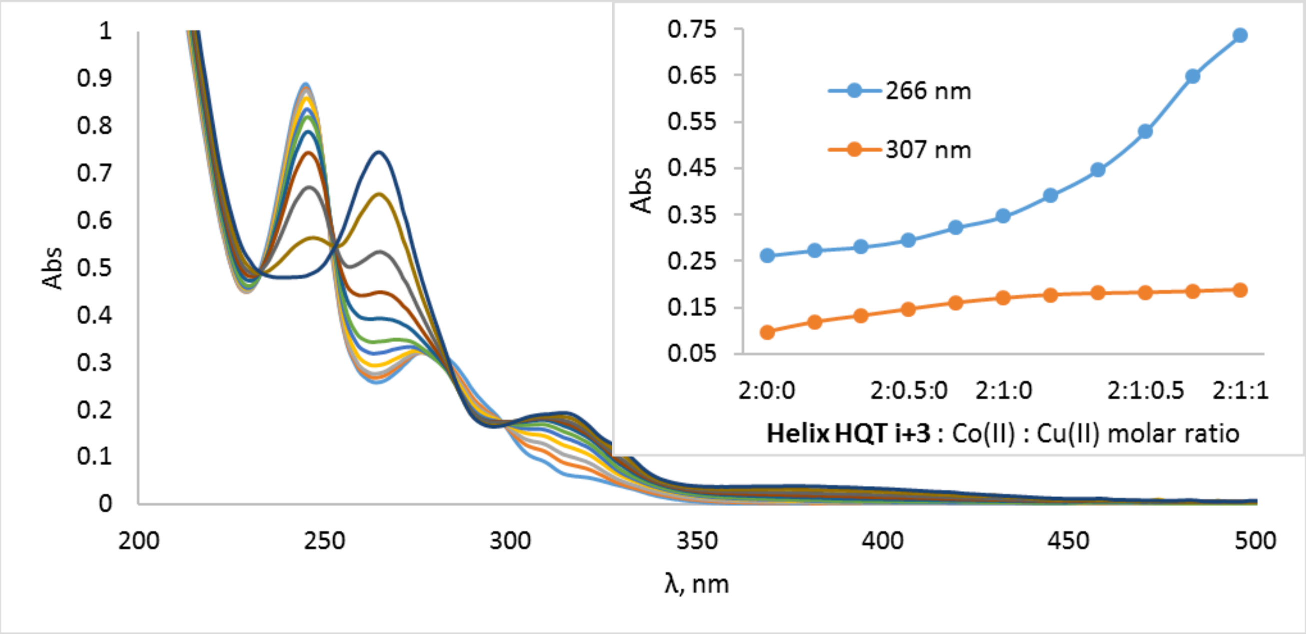

Figure S25. UV-Vis Titration and a peptoid-to-metal ratio plot of **Helix HQT i+3** (17 µM) with 0.5 equiv. of Co^{2+} (2.5 mM) followed by titration of 0.5 equiv. of Cu^{2+} (2.5 mM) in 3mL ACN.

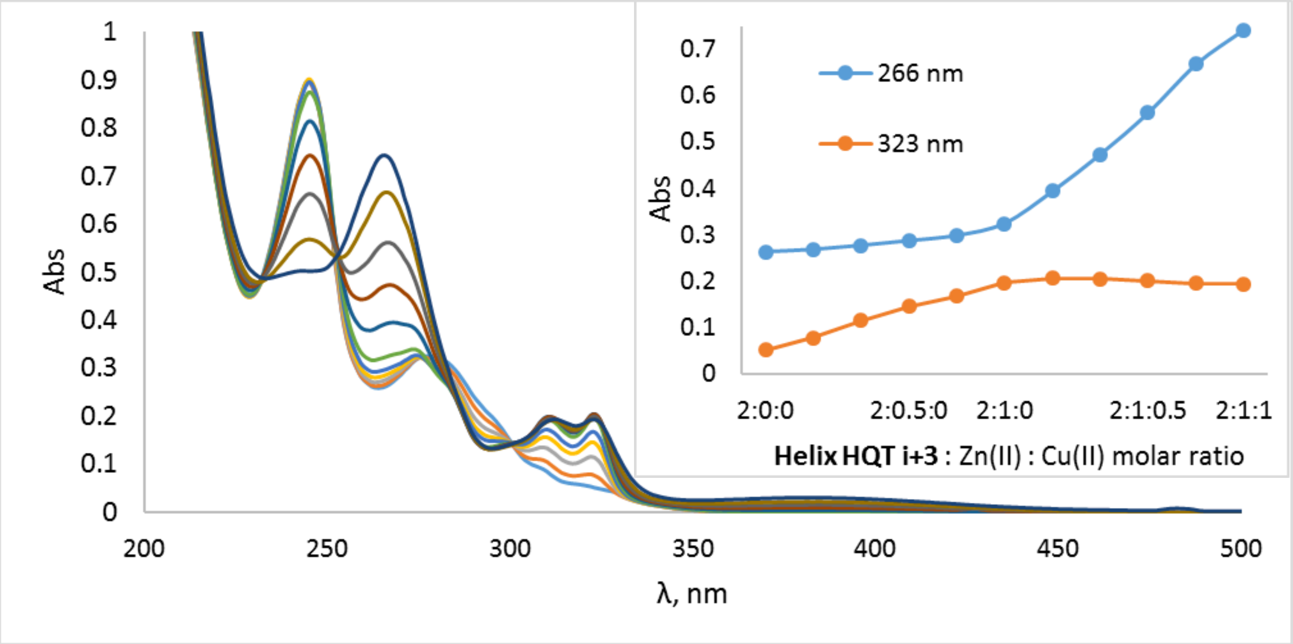

Figure S26. UV-Vis Titration and a peptoid-to-metal ratio plot of **Helix HQT i+3** (17 µM) with 0.5 equiv. of Zn^{2+} (2.5 mM) followed by titration of 0.5 equiv. of Cu^{2+} (2.5mM) in 3 ml ACN.

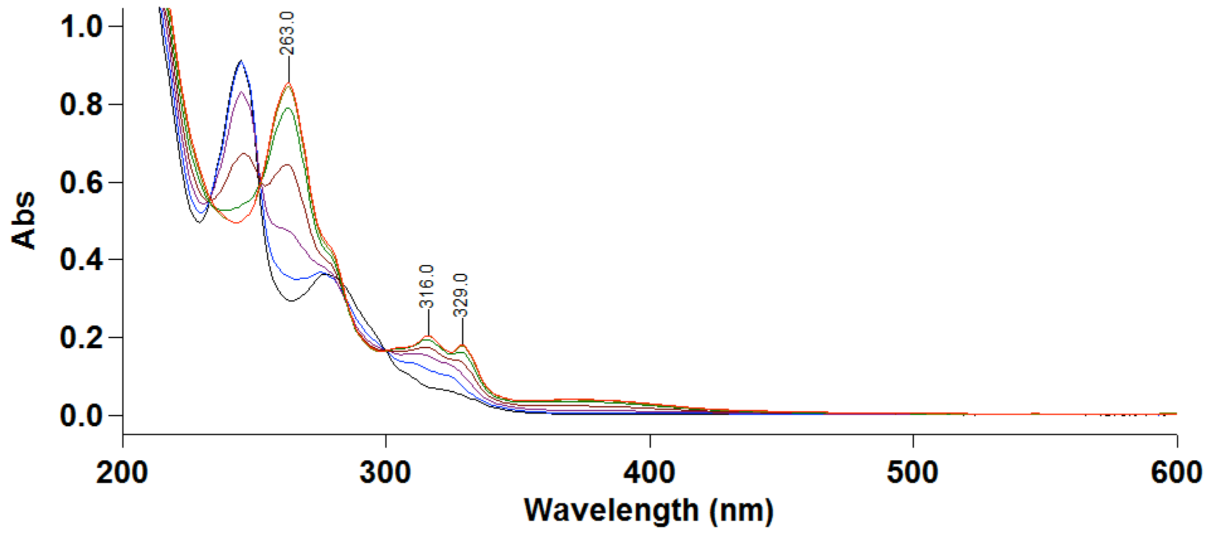

Figure S27. UV-Vis titration of **Helix HQT i+3** (17 µM) with Cu^{2+} acetate (5mM) in 3ml ACN.

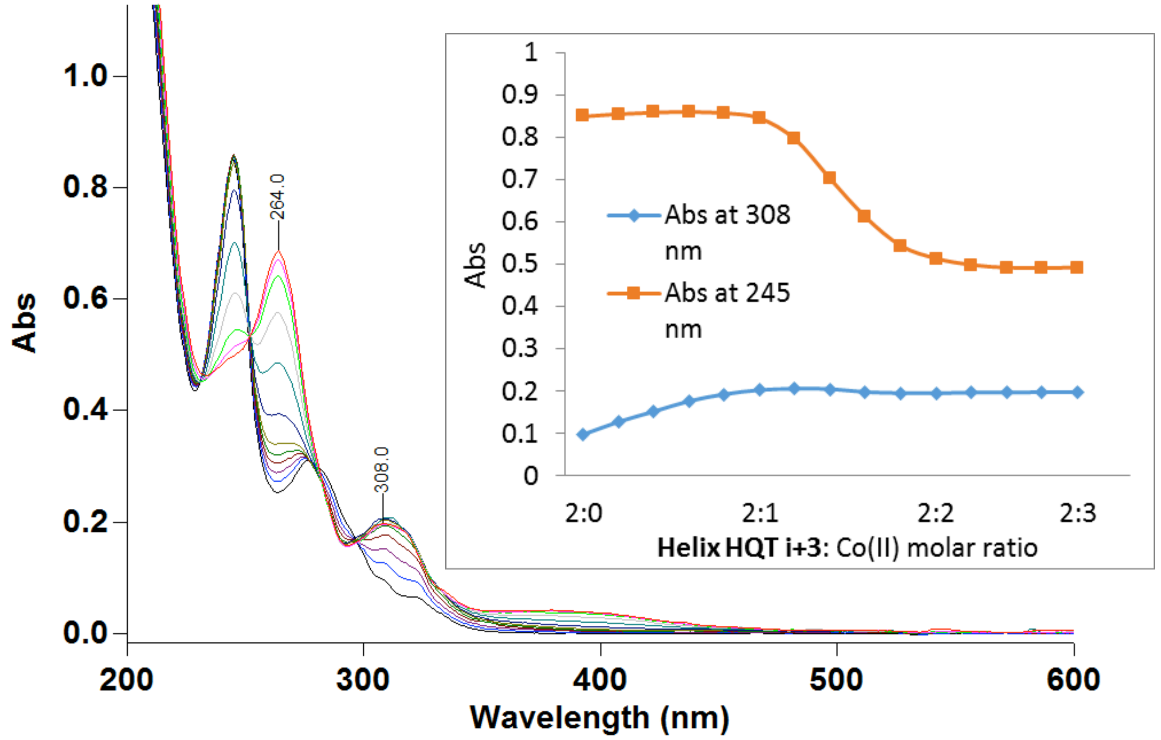

Figure S28. UV-Vis titration and a peptoid-to-metal ratio plot of **Helix HQT i+3** (17 µM) with Co^{2+} acetate (2.5mM) in 3ml ACN.

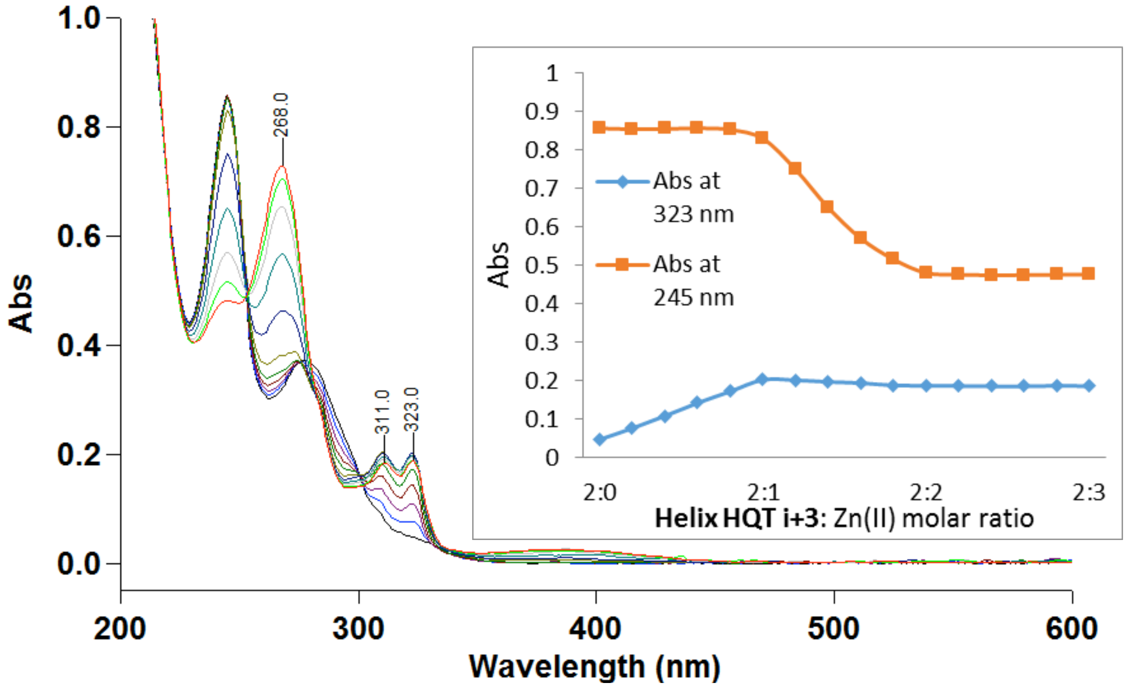

Figure S29. UV-Vis titration and a peptoid-to-metal ratio plot of **Helix HQT i+3** (17 µM) with Zn^{+2} acetate (2.5mM) in 3ml ACN.

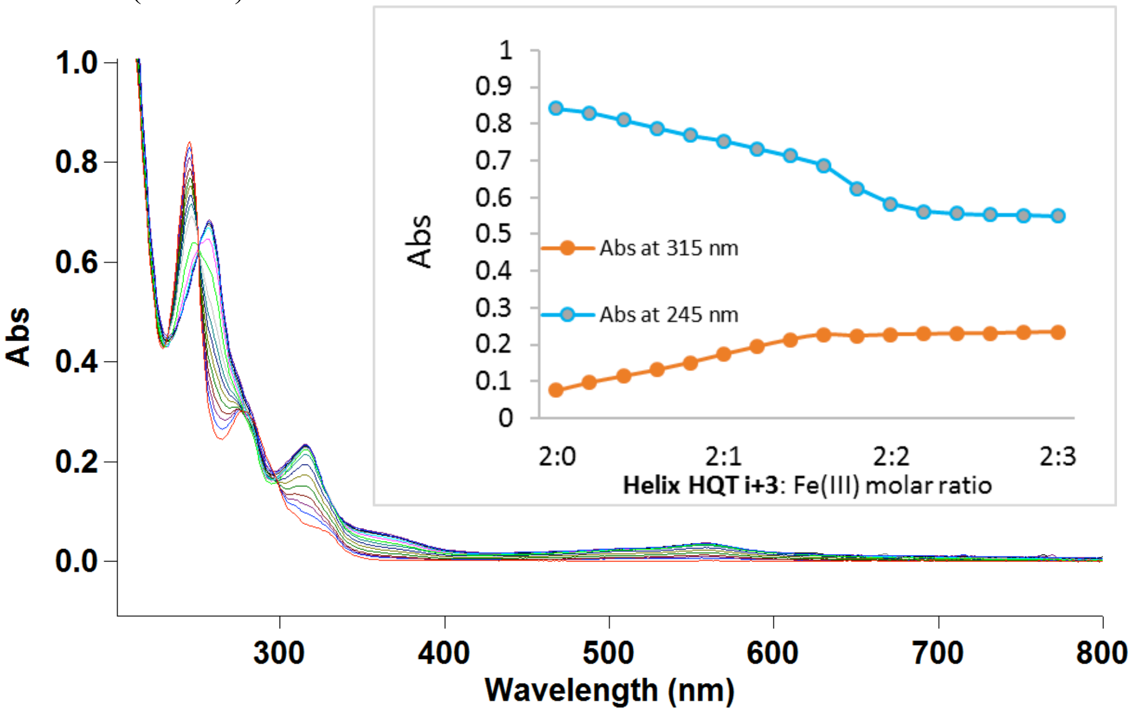

Figure S30. UV-Vis titration and a peptoid-to-metal ratio plot of **Helix HQT i+3** (17 µM) with Fe^{3+} perchlorate (2.5mM) in 3ml ACN.

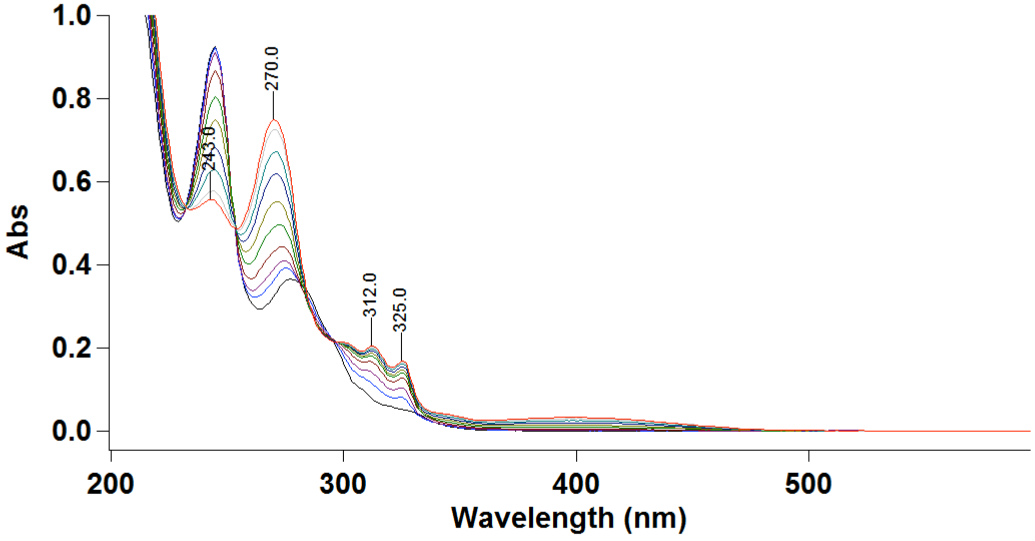

Figure S31. UV-Vis titration of **Helix HQT i+3** (17 µM) with Ni^{2+} acetate (2.5mM) in 3ml ACN.

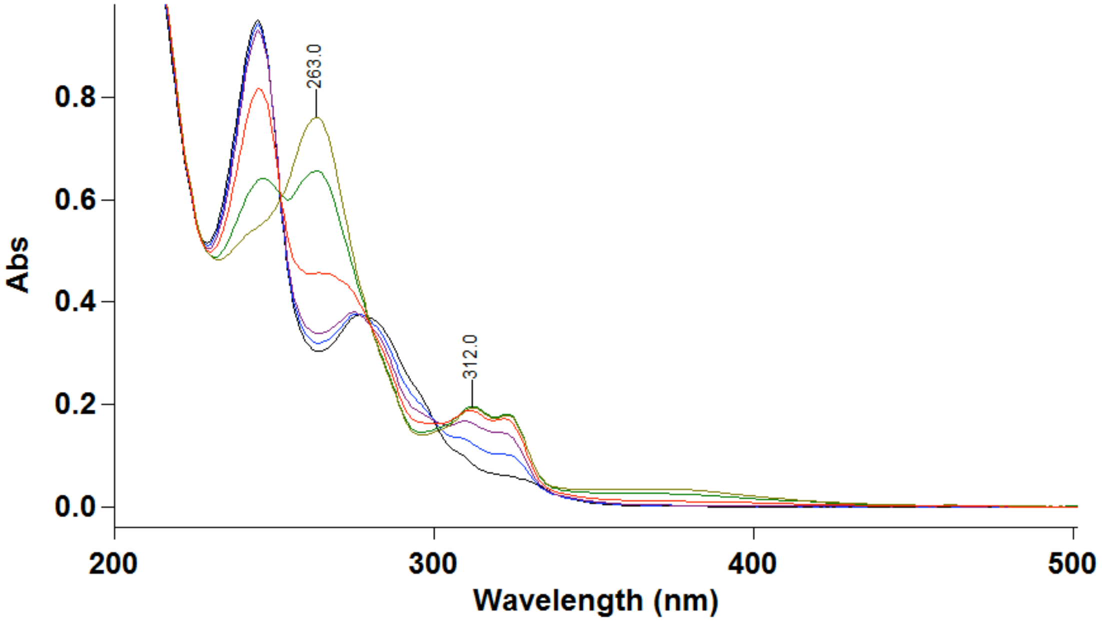

Figure S32. UV-Vis titration of **Helix HQT i+3** (17 µM) with Mn^{2+} acetate (5mM) in 3ml ACN.

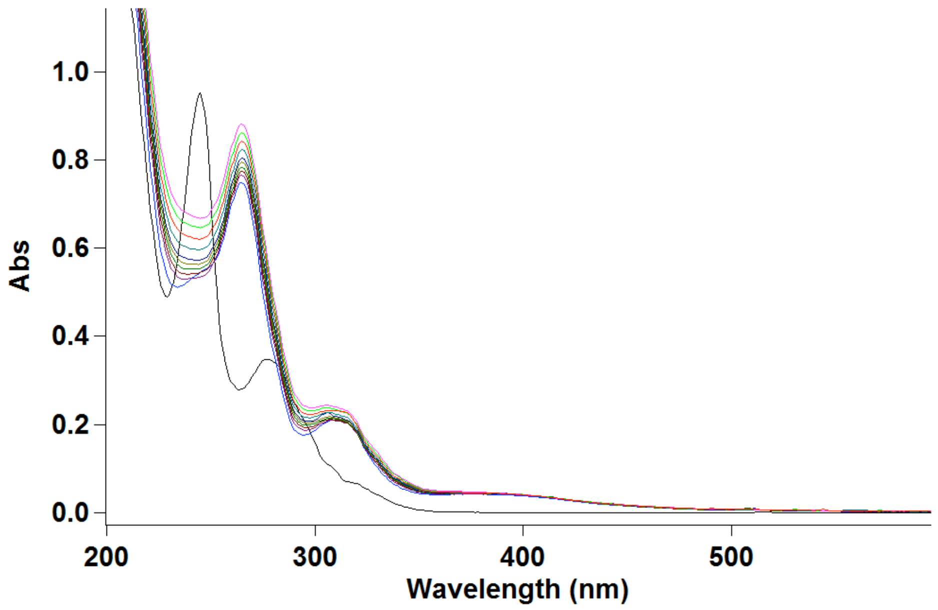

Figure S33. UV-Vis of free peptoid **Helix HQT i+3** (black, 17 μM), formation of (**Helix HQT i+3**)$_2$CoCu (blue) followed by titration with Cu^{+2} (2.5mM) in 3ml ACN.

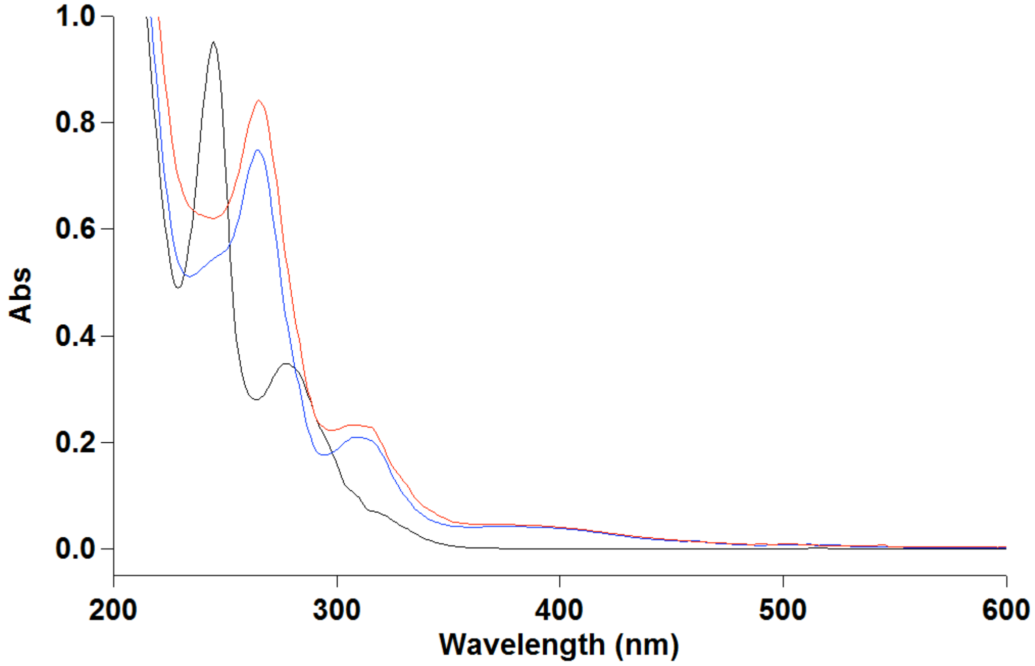

Figure S34. UV-Vis of **Helix HQT i+3** (black, 17 μM), (**Helix HQT i+3**)$_2$CoCu (blue) followed by addition of 2 equiv. of Cu^{+2} (red) in ACN.

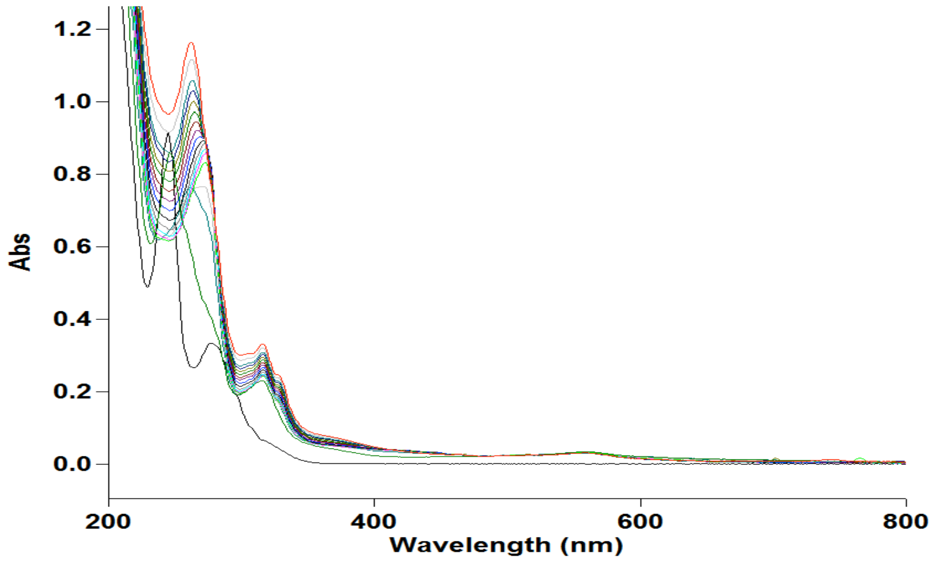

Figure S35. UV-Vis of free peptoid **Helix HQT i+3** (black, 17 μM), formation of (**Helix HQT i+3**)$_2$FeCu (blue) followed by titration with Cu^{+2} (2.5mM) in 3ml ACN.

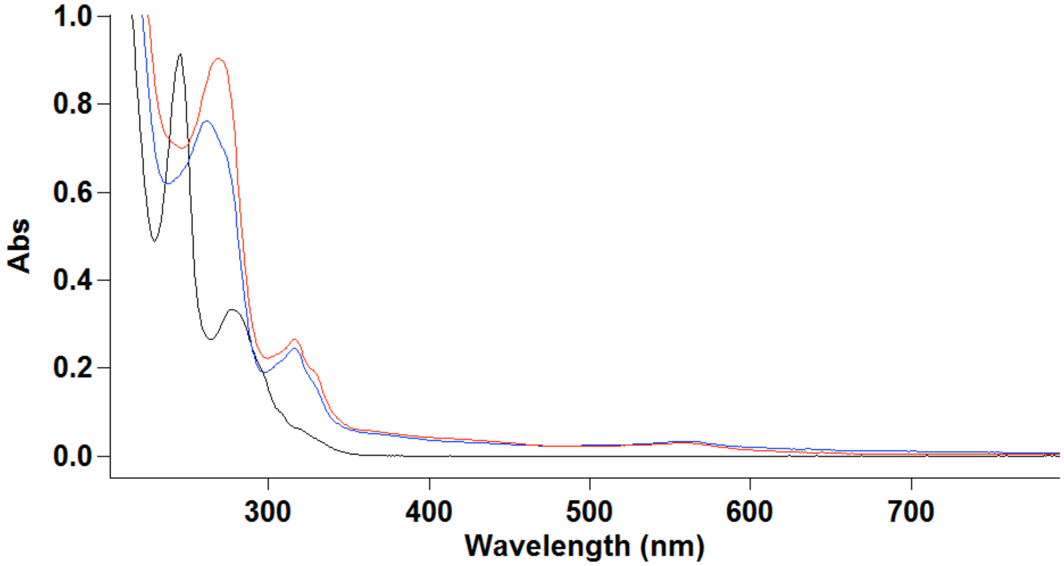

Figure S36. UV-Vis of **Helix HQT i+3** (black, 17 μM), (**Helix HQT i+3**)$_2$FeCu (blue line) followed by addition of 3 equiv. of Cu^{+2} (red) in 3ml ACN.

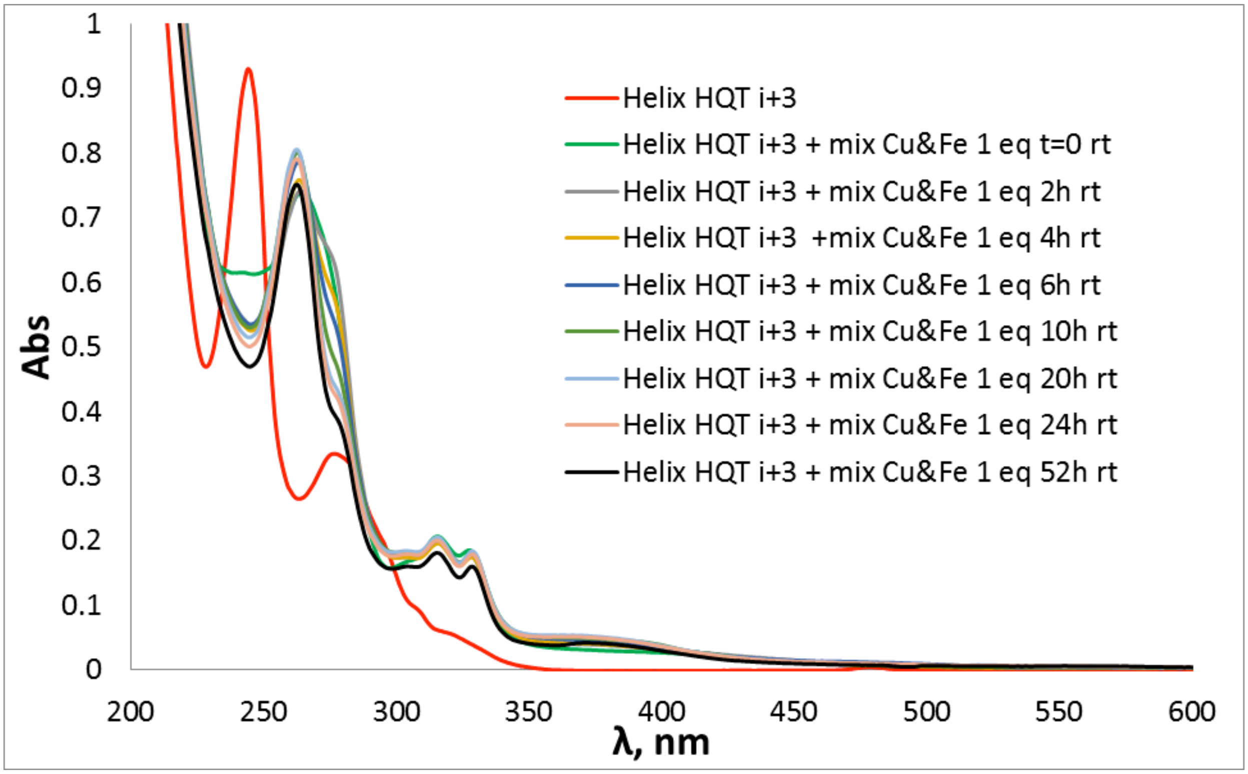

Figure S37. UV-Vis of **Helix HQT i+3** (17 μM) with mixture of 1 equiv. Cu^{2+} and Fe^{3+} in ACN at room temperature at different time points.

<u>UV-Vis of control peptoids in ACN</u>

Table S3. UV-Vis absorbance data of control peptoid oligomers and their metal complexes in ACN.

peptoid	Cu	Co	Zn	mix Cu&Co	mix Cu&Zn
**Non helix HQT i+3**	263, 316, 329 (Fig S38)	264, 30 (Fig S39)	268, 311, 323 (Fig S40)	262, 309 (Fig S41)	264, 314 (Fig S42)
**DI HQT**	263, 315, 328 (Fig S43)	264, 307 (Fig S44)	367, 311, 322 (Fig S45)	263, 315 (Fig S46)	264, 313 (Fig S47)
**Helix HQT i+2**	264, 314 (Fig S48)	266, 310 (Fig S49)	268, 310, 321 (Fig S50)	265, 311 (Fig S51)	267, 310 (Fig S52)
**Helix HQT i+4**	265, 316 (Fig S53)	266, 314 (Fig S54)	267, 309, 321 (Fig S55)	265, 310 (Fig S56)	267, 310, 321 (Fig S57)

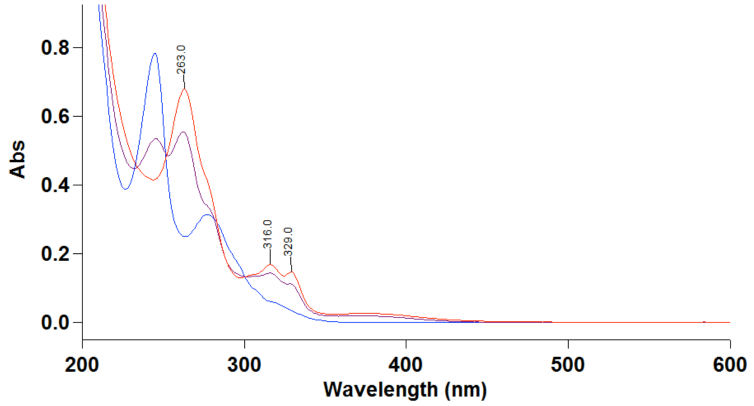

Figure S38. UV-Vis of **Nonhelix HQT i+3** (blue, 17 µM) in ACN with 0.5 (purple) and 1 equiv. of Cu^{2+} (red).

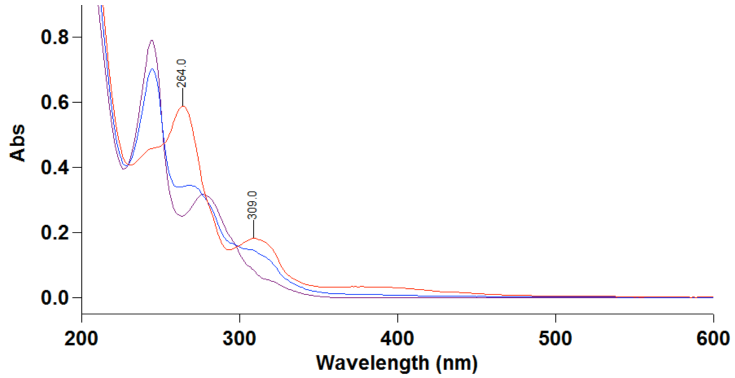

Figure S39. UV-Vis of **Nonhelix HQT i+3** (purple, 17 µM) in ACN with 0.5 (blue) and 1.5 equiv. of Co^{2+} (red).

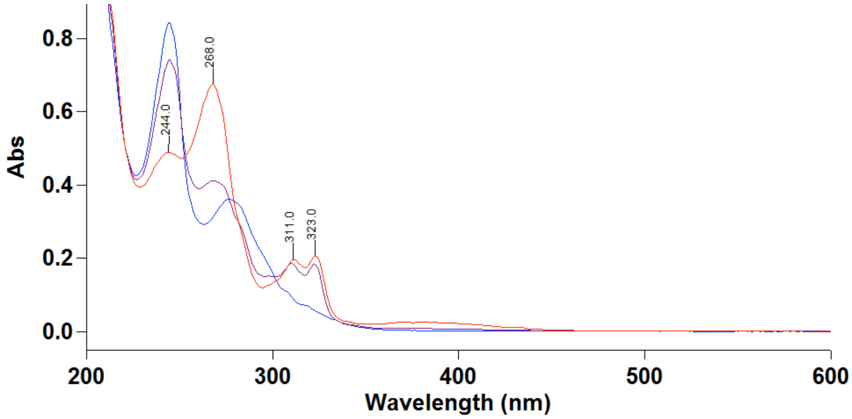

Figure S40. UV-Vis of **Nonhelix HQT i+3** (blue, 17 µM) in ACN with 0.5 (purple) and 1 equiv. of Zn^{2+} (red).

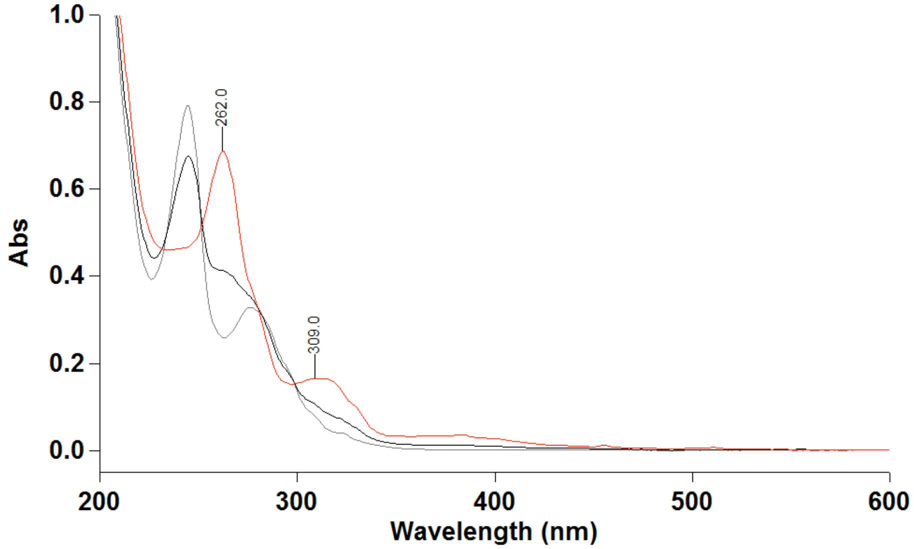

Figure S41. UV-Vis of free **NonHelix HQT i+3** (gray, 17 μM) with 0.5 equiv. mix of Cu^{2+} and Co^{2+} (black) and 1equiv. mix of Cu^{2+} and Co^{2+} (red) in ACN.

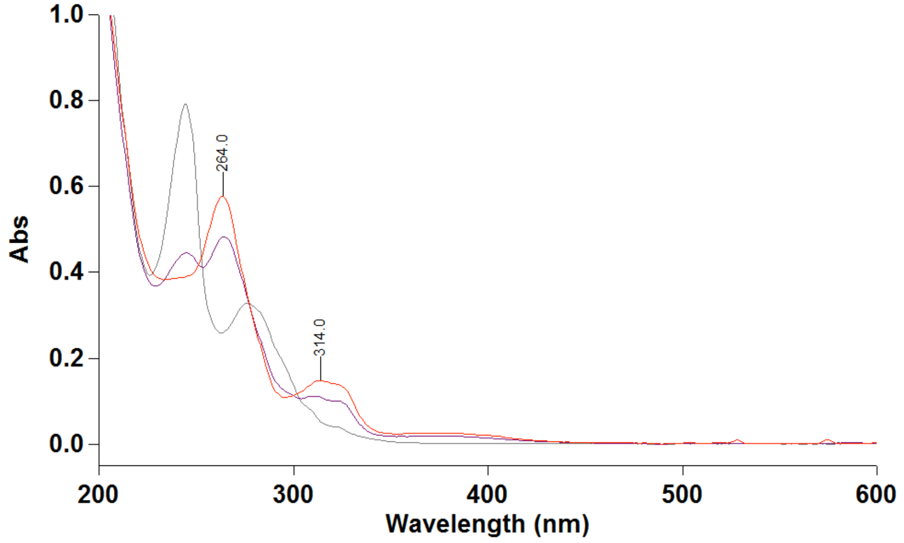

Figure S42. UV-Vis of free **NonHelix HQT i+3** (gray, 17 μM) with 0.5 equiv. mix of Cu^{2+} and Zn^{2+} (purple) and 1 equiv. mix of Cu^{2+} and Zn^{2+} (red) in ACN.

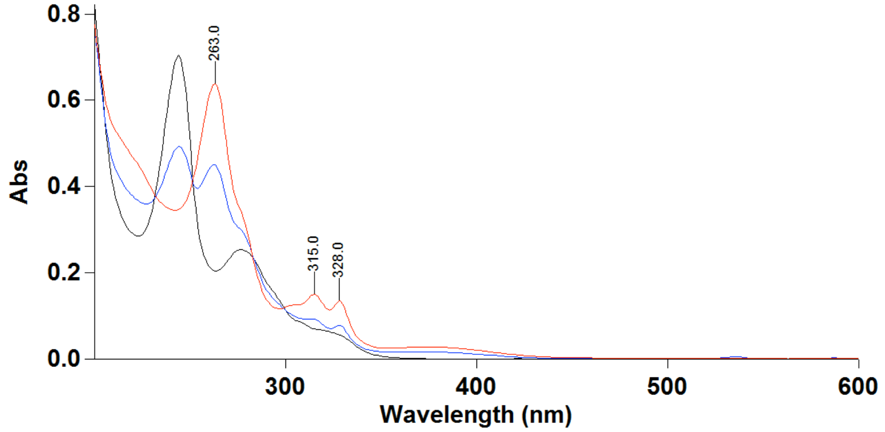

Figure S43. UV-Vis of **DI HQT** (black, 17 μM) in ACN with 0.5 (blue) and 1equiv. of Cu^{2+} (red).

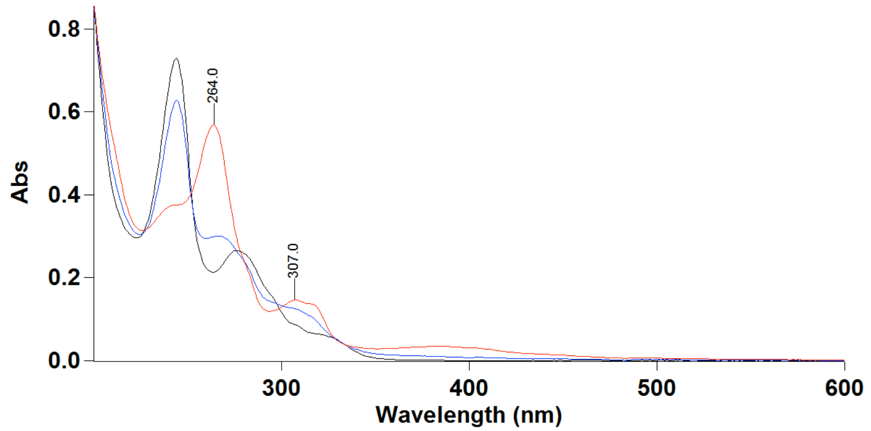

Figure S44. UV-Vis of **DI HQT** (black, 17 μM) in ACN with 0.5 (blue) and 1.5 equiv. of Co^{2+} (red).

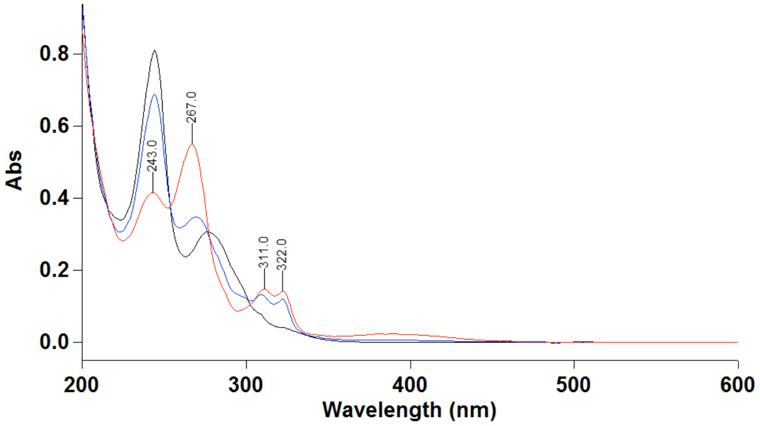

Figure S45. UV-Vis of **DI HQT** (black, 17 μM) in ACN with 0.5 (blue) and 1.5 equiv. of Zn^{2+} (red).

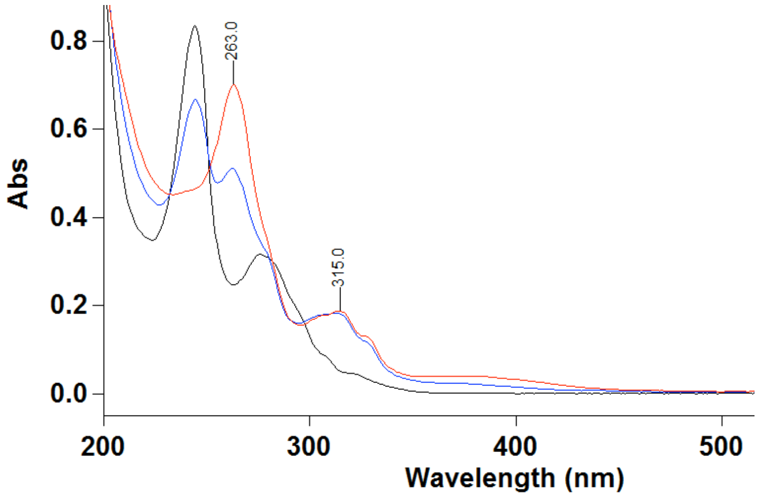

Figure S46. UV-Vis of free **DI HQT** (black, 17 µM) with 0.5 equiv. mix of Cu^{+2} and Co^{+2} (blue) and 1 mix of Cu^{+2} and Co^{+2} (red) in ACN.

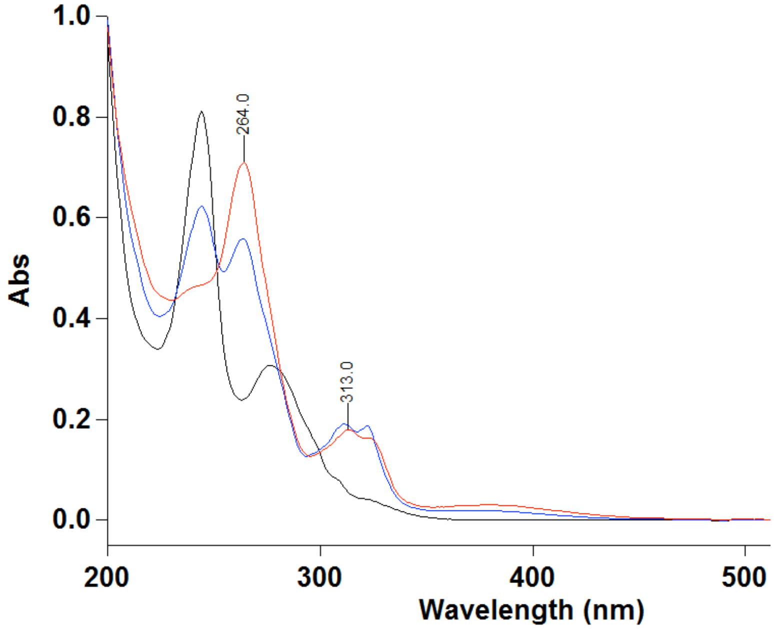

Figure S47. UV-Vis of free **DI HQT** (black, 17 µM) with 0.5 equiv. mix of Cu^{2+} and Zn^{2+} (blue) and 1 equiv. mix of Cu^{2+} and Zn^{2+} (red) in ACN.

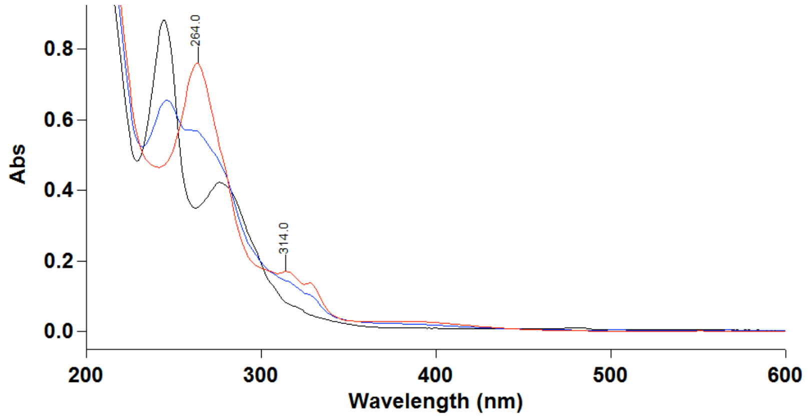

Figure S48. UV-Vis of **Helix HQT i+2** (black, 17 µM) in ACN with 0.5 (blue) and 1 equiv. of Cu^{2+} (red).

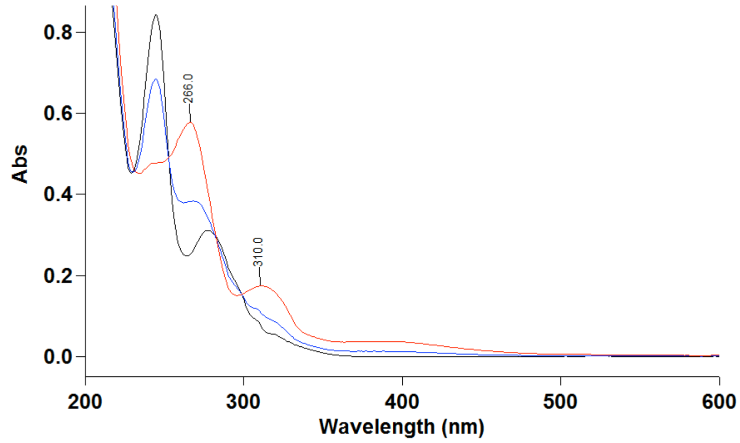

Figure S49. UV-Vis of **Helix HQT i+2** (black, 17 µM) in ACN with 0.5 (blue) and 1.5 equiv. of Co^{2+} (red).

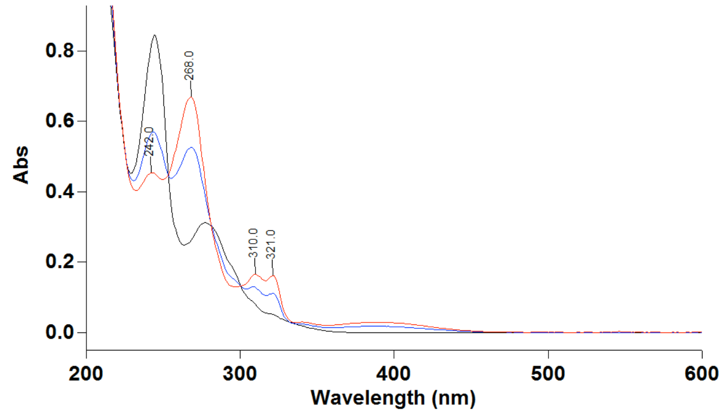

Figure S50. UV-Vis of **Helix HQT i+2** (black, 17 µM) in ACN with 0.5 (blue) and 1 equiv. of Zn^{2+} (red).

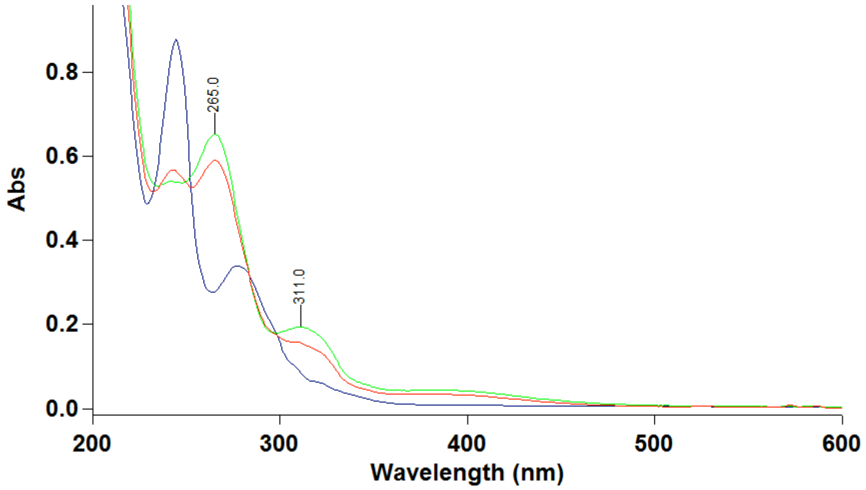

Figure S51. UV-Vis of free **Helix HQT i+2** (blue, 17 µM) with 0.5 equiv. mix of Cu^{2+} and Co^{2+} (red) and 1equiv. mix of Cu^{2+} and Co^{2+} (green) in ACN.

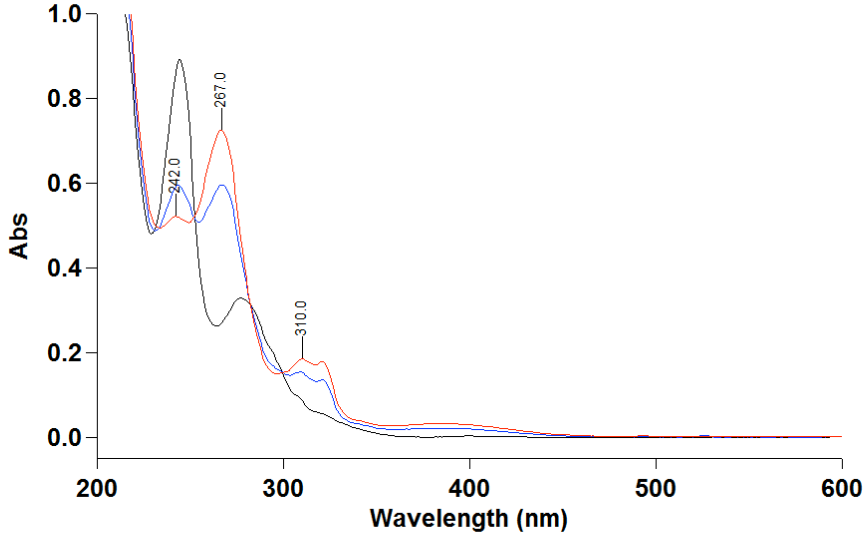

Figure S52. UV-Vis of free **Helix HQT i+2** (black, 17 µM) with 0.5 equiv. mix of Cu^{2+} and Zn^{2+} (blue) and 1 equiv. mix of Cu^{2+} and Zn^{2+} (red) in ACN.

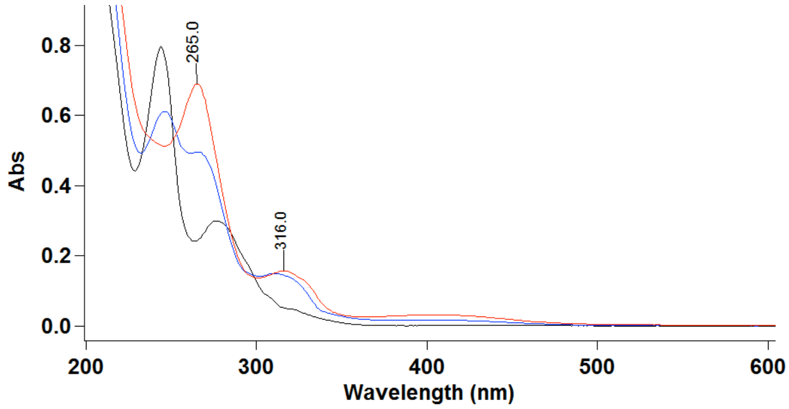

Figure S53. UV-Vis of **Helix HQT i+4** (black, 17 µM) in ACN with 0.5 (blue) and 1 equiv. of Cu^{2+} (red).

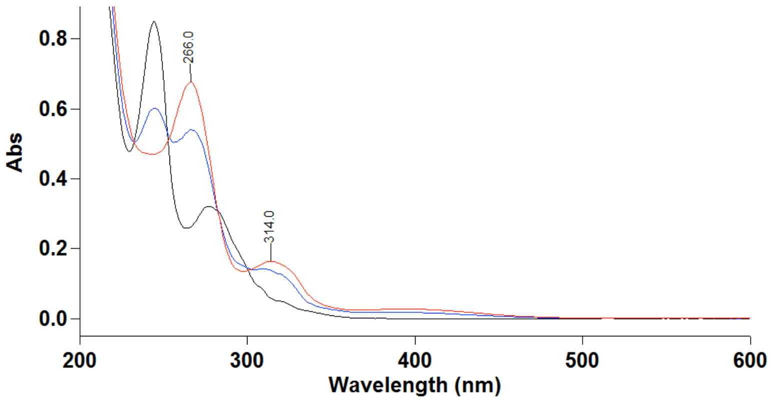

Figure S54. UV-Vis of **Helix HQT i+4** (black, 17 µM) in ACN with 0.5 (blue) and 1 equiv. of Co^{2+} (red).

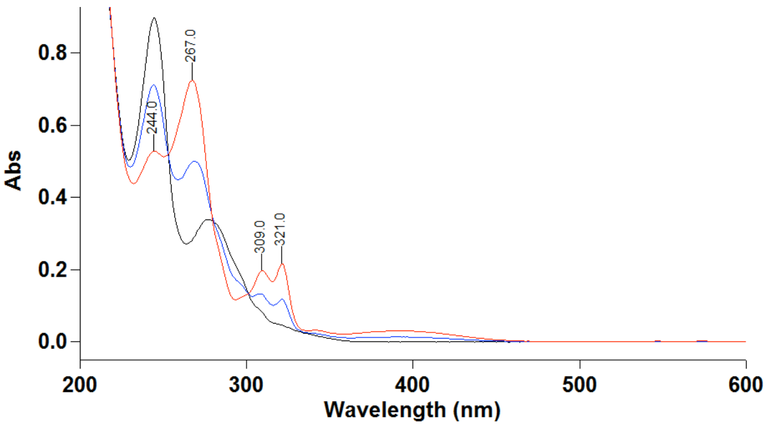

Figure S55. UV-Vis of **Helix HQT i+4** (black, 17 µM) in ACN with 0.5 (blue) and 1 equiv. of Zn^{2+} (red).

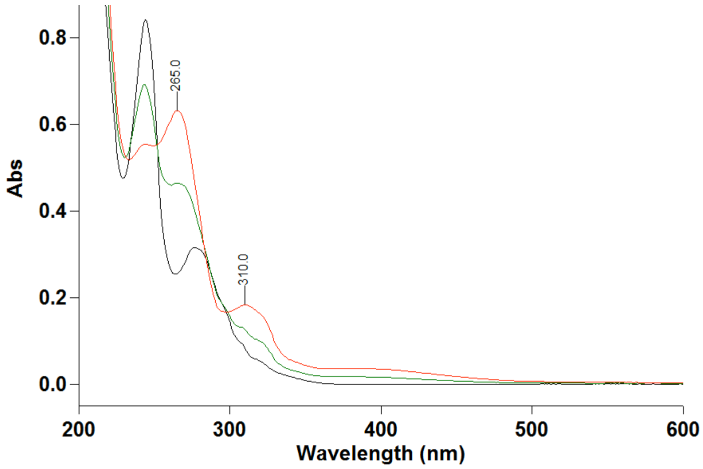

Figure S56. UV-Vis of free **Helix HQT i+4** (black, 17 μM) with 0.5 equiv. mix of Cu^{2+} and Co^{2+} (green) and 1 equiv. mix of Cu^{2+} and Co^{2+} (red) in ACN.

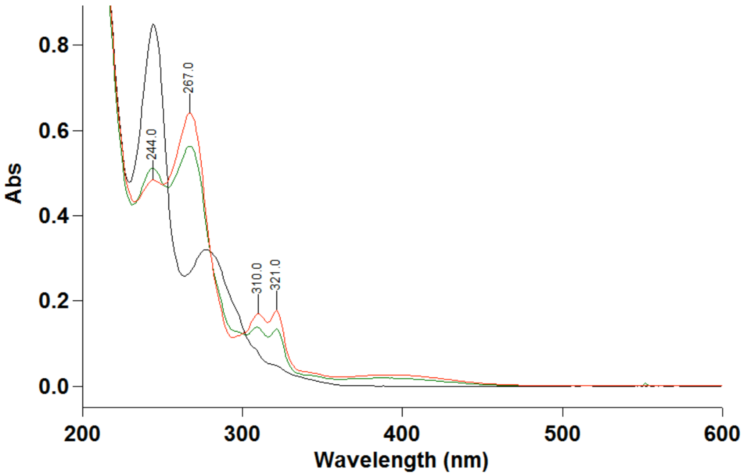

Figure S57. UV-Vis of free **Helix HQT i+4** (black, 17 μM) with 0.5 equiv. mix of Cu^{2+} and Zn^{2+} (green) and 1 equiv. mix of Cu^{2+} and Zn^{2+} (red) in 3ml ACN.

Association constants

Table S4. The association constants for metal binding were obtained by fitting the UV titrations results to the equation below using a nonlinear regression (curve fit) GraphPad Prism® software. Where A_0 and A_{max} are the minimum and maximum absorbance measured for the free ligand respectively, L_0 is the initial concentration of the free ligand (typically 7 μM) and M is the concentration of metal ions that was added (0-290 μM).

$$A = A_0 + \frac{1}{2[L_0]}(A_{max} - A_0) \times \left[\left([L_0] + [M] + \frac{1}{K} \right) \pm \sqrt{\left([L_0] + [M] + \frac{1}{K} \right)^2 - 4[L_0][M]} \right]$$

(Peptoid)M	A_0, A_{max}	Binding constant K [M^{-1}]	R square
(**Helix HQT i+3**)Cu	0.426, 0.303	$(1.03 \pm 0.49)x10^{13}$	0.9986
(**Helix HQT i+3**)Zn	0.378, 0.187	$(3.60 \pm 0.46)x10^{11}$	0.9938
(**Helix HQT i+3**)Co	0.350, 0.176	$(2.53 \pm 0.35)x10^{11}$	0.9916
(**Helix HQT i+3**)Ni	0.373, 0.149	$(1.52 \pm 0.09)x10^{10}$	0.9971
(**Helix HQT i+3**)Mn	0.362, 0.162	$(1.13 \pm 0.06)x10^{10}$	0.9981
(**DI HQT**)Cu	0.292, 0.136	$(1.36 \pm 0.66)x10^{12}$	0.9773
(**DI HQT**)Zn	0.330, 0.184	$(1.28 \pm 0.49)x10^{12}$	0.9806
(**DI HQT**)Co	0.324, 0.226	$(5.15 \pm 0.76)x10^{11}$	0.9984
(**DI HQT**)Ni	0.333, 0.174	$(1.33 \pm 0.18)x10^{9}$	0.9916
(**DI HQT**)Mn	0.316, 0.214	$(1.04 \pm 0.28)x10^{9}$	0.9742
(**Nonhelix HQT i+3**)Cu	0.398, 0.297	$(1.16 \pm 0.53)x10^{12}$	0.9874
(**Nonhelix HQT i+3**)Zn	0.312, 0.193	$(1.07 \pm 0.68)x10^{12}$	0.9467
(**Nonhelix HQT i+3**)Co	0.305, 0.175	$(1.69 \pm 0.56)x10^{12}$	0.9895
(**Nonhelix HQT i+3**)Ni	0.316, 0.154	$(6.03 \pm 0.69)x10^{10}$	0.9892
(**Nonhelix HQT i+3**)Mn	0.316, 0.203	$(2.65 \pm 0.28)x10^{10}$	0.9952
(**Helix HQT i+4**)Cu	0.321, 0.248	$(4.12 \pm 0.84)x10^{11}$	0.9939
(**Helix HQT i+4**)Zn	0.375, 0.261	$(1.04 \pm 0.17)x10^{11}$	0.9878

(**Helix HQT i+4**)Co	0.298, 0.224	$(1.40\pm0.39)x10^{11}$	0.9803
(**Helix HQT i+4**)Ni	0.316, 0.199	$(2.67 \pm 0.75)x10^{10}$	0.9551
(**Helix HQT i+4**)Mn	0.293, 0.171	$(8.13 \pm 1.77)x10^{10}$	0.9629
(**Helix HQT i+2**)Cu	0.373, 0.221	$(1.50\pm0.37)x10^{12}$	0.9933
(**Helix HQT i+2**)Zn	0.379, 0.278	$(1.00\pm0.37)x10^{12}$	0.9823
(**Helix HQT i**+2)Co	0.366, 0.228	$(6.01\pm2.23)x10^{11}$	0.9815
(**Helix HQT i**+2)Ni	0.363, 0.247	$(1.15 \pm 0.39)x10^{10}$	0.9681
(**Helix HQT i**+2)Mn	0.381, 0.221	$(5.37 \pm 0.64)x10^{10}$	0.9913
(**$H_2$6**)Cu	0.319, 0.119	$(1.43\pm0.46)x10^{12}$	0.9896

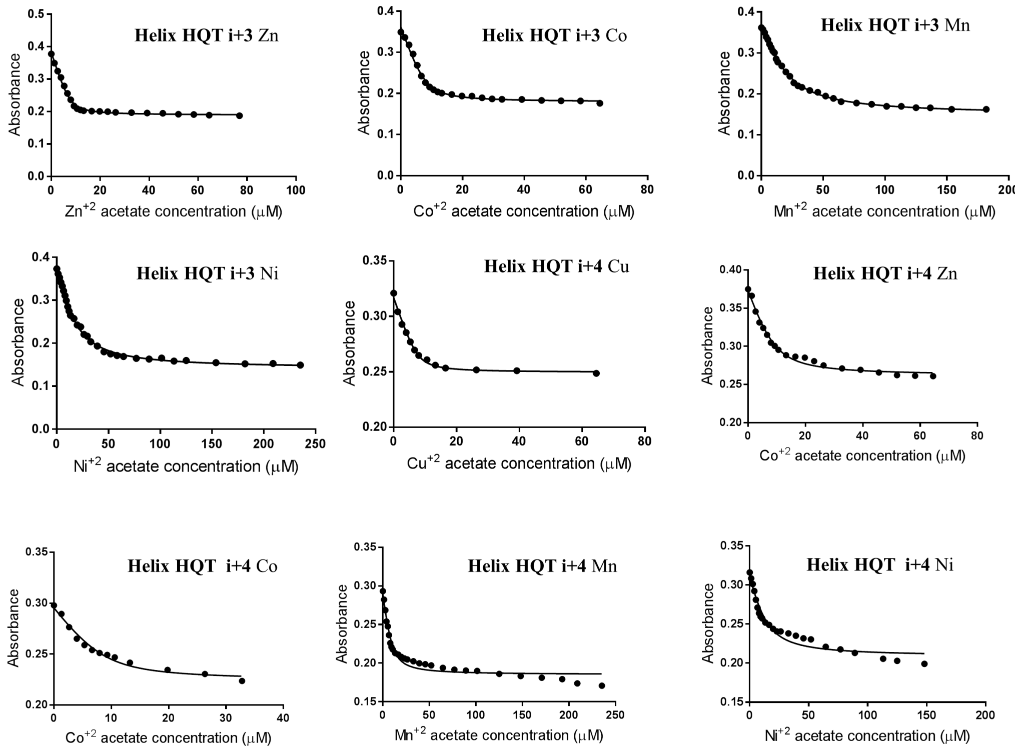

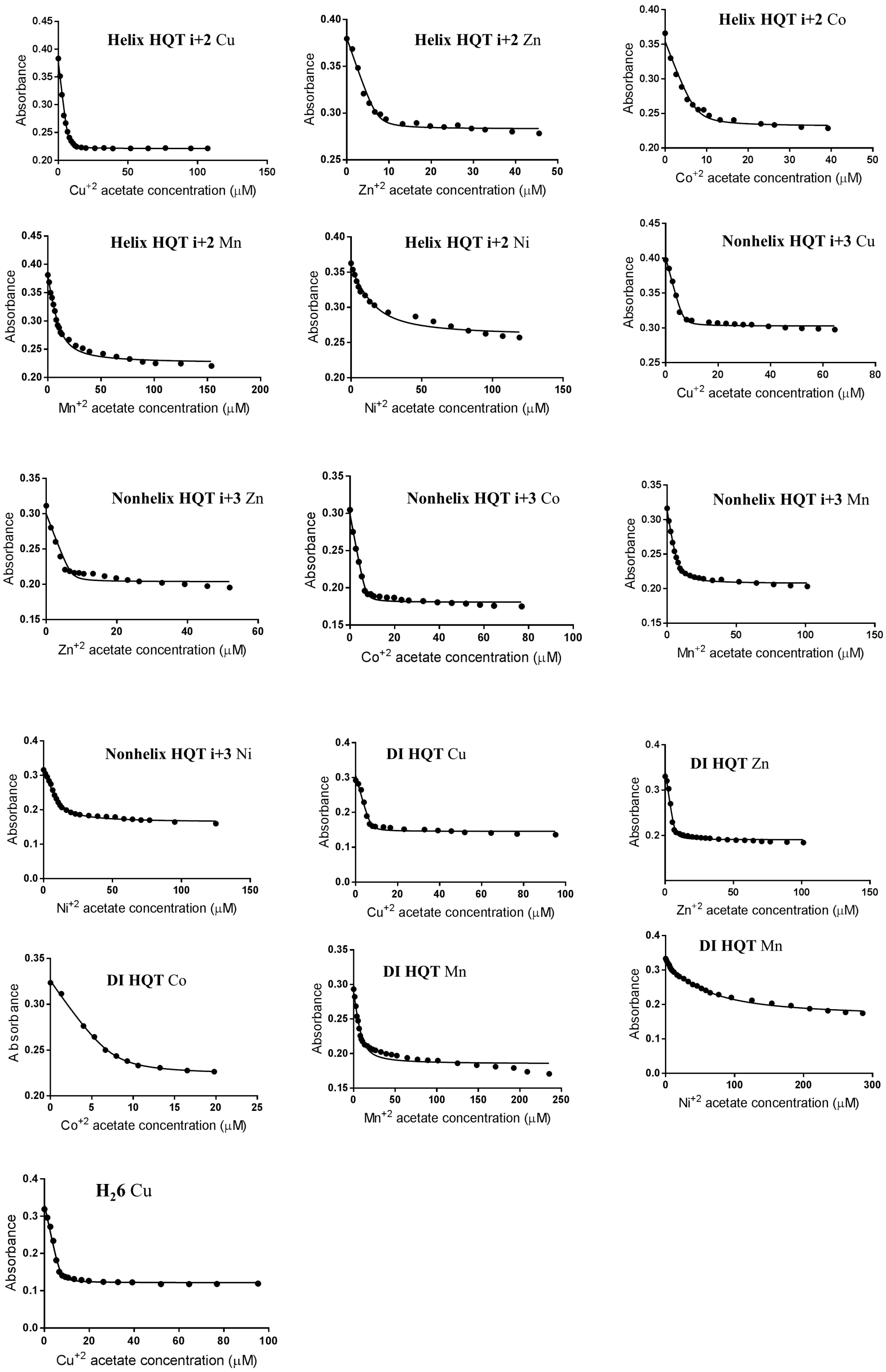

Figure S58. Association curve (dots) and fit (line) for the formation of peptoid metal complexes. The fitting was done using a nonlinear regression (curve fit) GraphPad Prism® software.

EPR

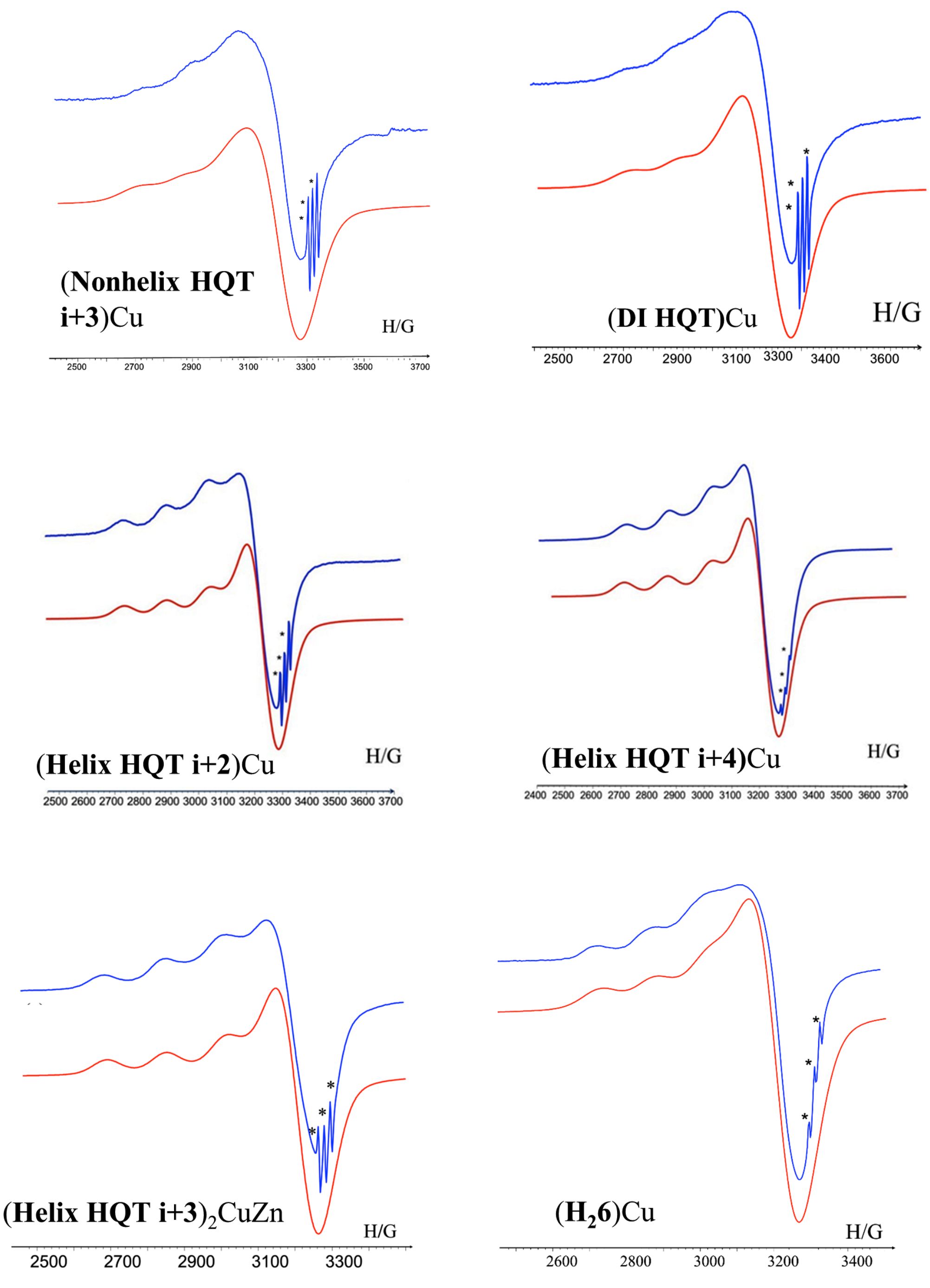

Figure S59. Room temperature X-band EPR spectra of copper(II) complexes (blue lines) and corresponding simulated spectra (red lines). Reference- TEMPO marked by*, g = 2.0058.

ESI-MS

Table S5. Peptoid oligomer sequences and ESI-MS analysis. *N*hq = 8-hydroxy-2-quinolinemethylamine, *Nspe* = (S)-(-)-1-Phenylethylamine, *Nterpy* = 2,2':6',2"-Terpyridineamine. *Npm* = N-benzylamine and *Nme* = N-methoxyamine.

Peptoid	Molecular weight Calc.: Found (gr/mol)	Figure #
Nspe-Nterpy-Nspe-Nspe-Nhq-Nspe (**Helix HQT i+3**)	1208.4 : 1208.0	S60
Nme-Nterpy-Npm-Nme-Nhq-Npm (**Nonhelix HQT i+3**)	1088.2 : 1087.8	S61
Nterpy-Nhq **(DI HQT)**	563.6 : 564.1	S62
Nspe- Nspe-Nspe- Nterpy-Nspe -Nhq (**Helix HQT i+2**)	1208.4 : 1209.0	S63
Nspe-Nterpy-Nspe-Nspe- Nspe -Nhq **(Helix HQT i+4)**	1208.4 : 1209.0	S64

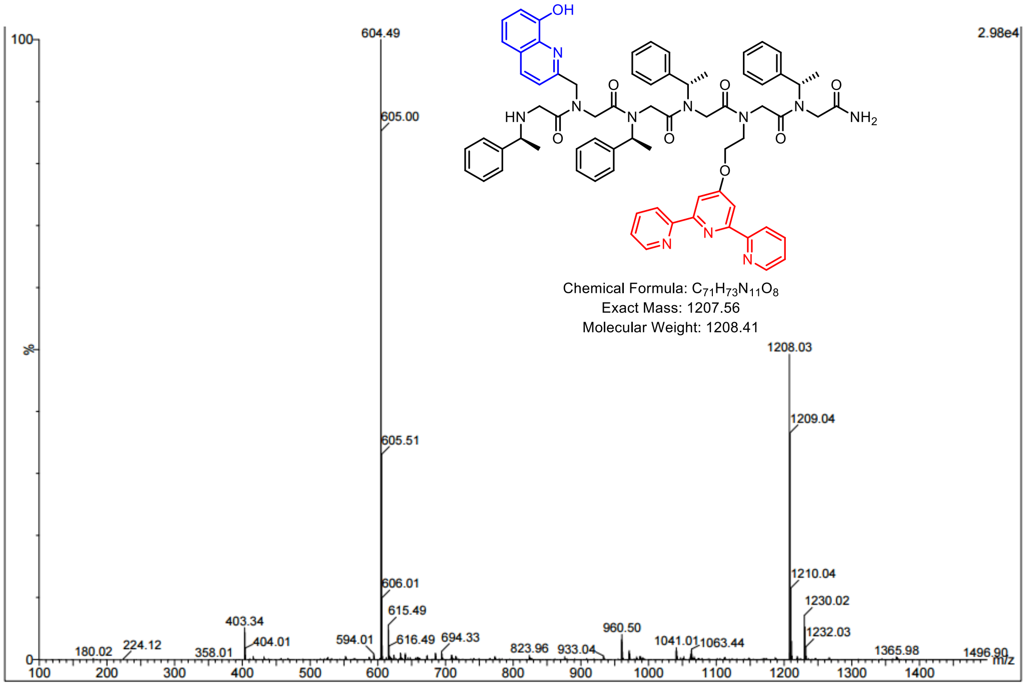

Figure S60. ESI-MS traces of peptoid oligomer **Helix HQT i+3**.

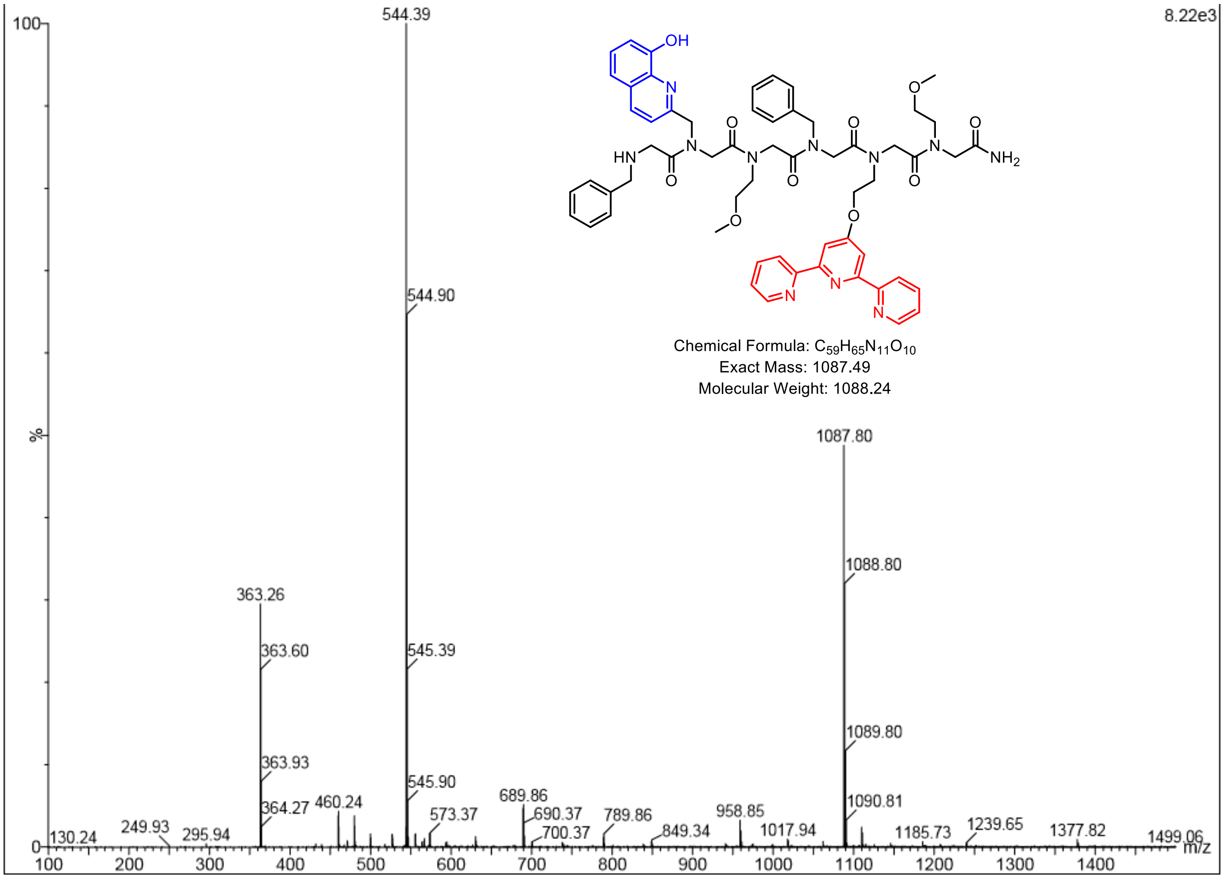

Figure S61. ESI-MS traces of peptoid oligomer **Nonhelix HQT i+3**.

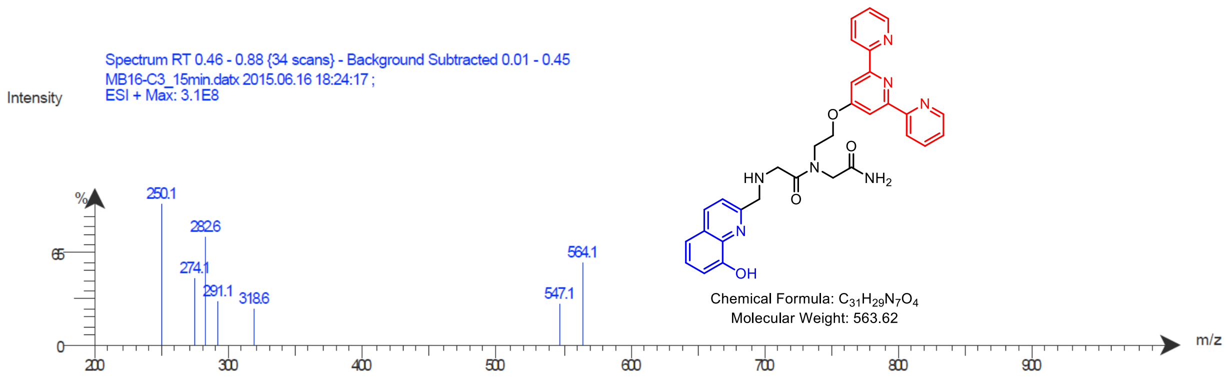

Figure S62. ESI-MS traces of peptoid oligomer **DI HQT**.

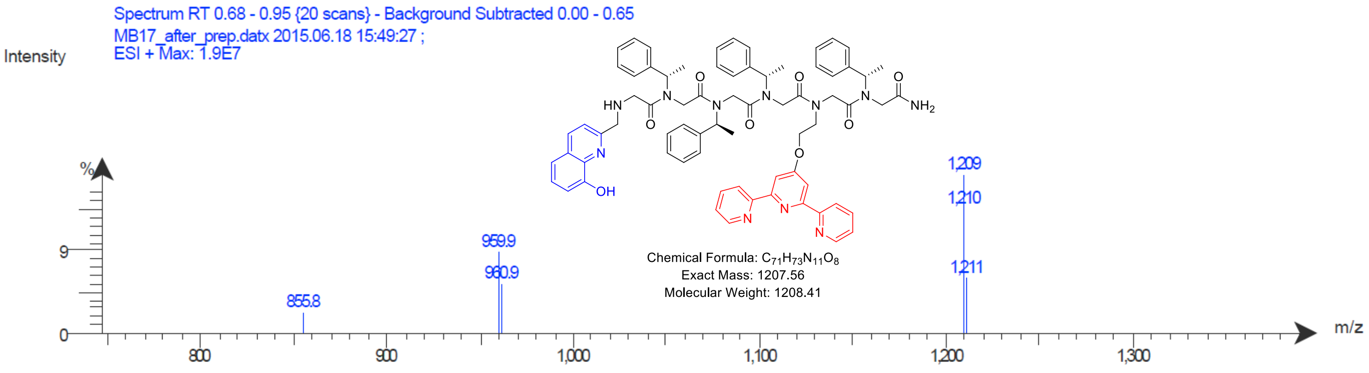

Figure S63. ESI-MS traces of peptoid oligomer **Helix HQT i+2**.

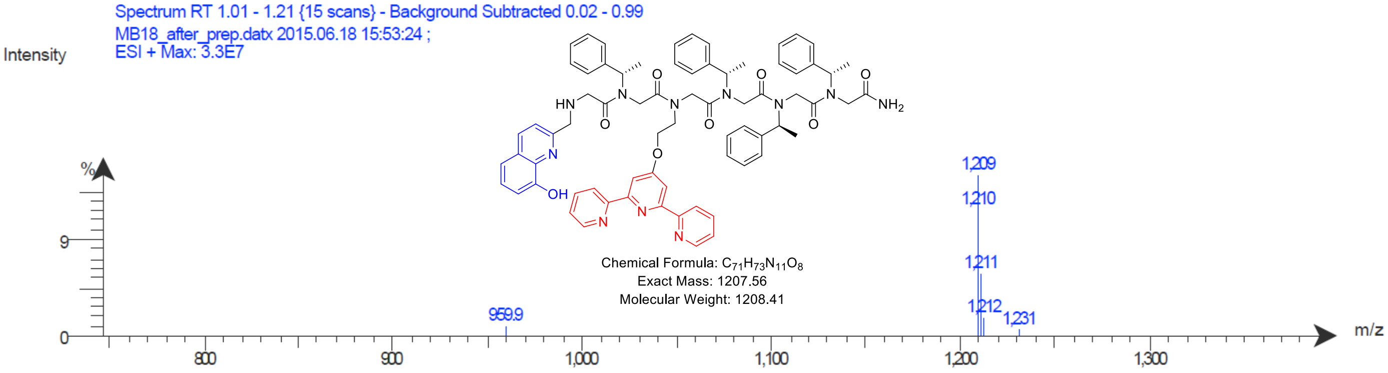

Figure S64. ESI-MS traces of peptoid oligomer **Helix HQT i+4**.

Table S6. ESI-MS analysis of **Helix HQT i and i+3** metal complexes

Metallopeptoid	Molecular weight	# Figure
	Calc.: Found (gr/mol)	
(**Helix HQT i+3**)Cu	1269.49 : 1269.56	S65-66
(**Helix HQT i**+3)Co	1265.49 : 1265.40	S67-68
(**Helix HQT i**+3)Zn	1270.49 : 1270.41	S69-70
(**Helix HQT i+*3***)Fe	1362.39 (m/2z) : 1362.44 (m/2z)	S71-77
(**Helix HQT i+3**)Ni	1264.49 : 1264.41	S73-74
(**Helix HQT i**+3)Mn	1261.49: 1261.40	S75-S76
(**Helix HQT i+3**)$_2$CuCo	846.67(m/3z) : 846.58 (m/3z)	S85-86
(**Helix HQ i+3**)$_2$CuZn	848.33(m/3z) : 848.24 (m/3z)	S87-89
(**Helix HQT i+3**)$_2$CuFe	844.66(m/3z) : 844.61 (m/3z)	S90-91

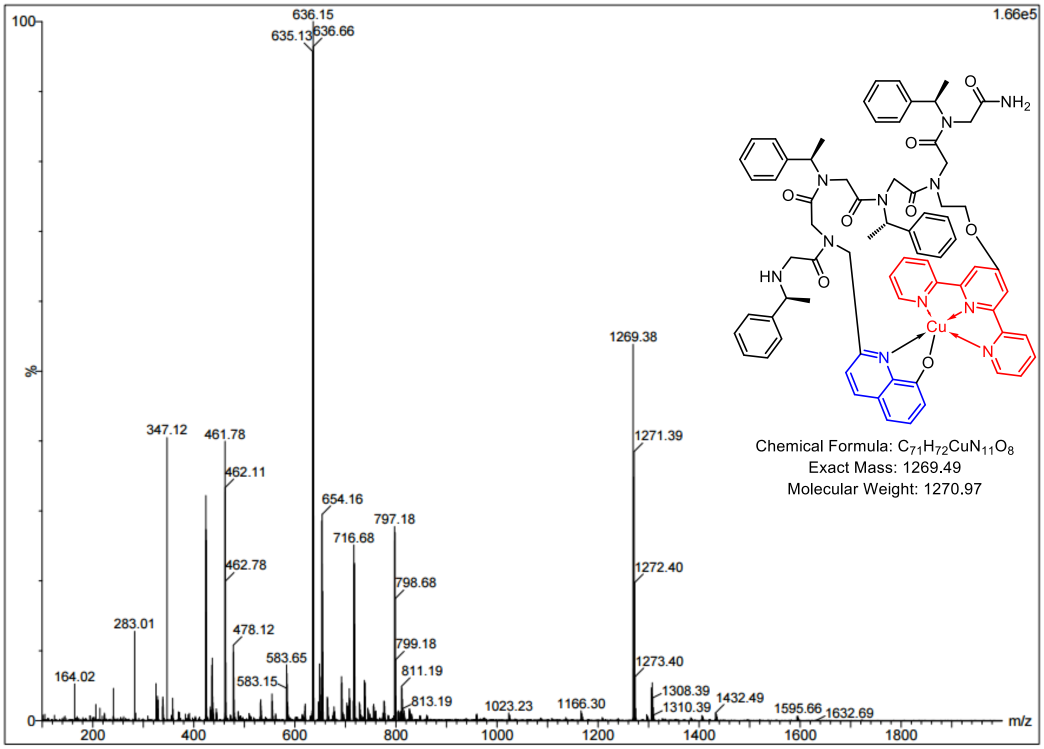

Figure S65. ESI-MS traces of (**Helix HQT i+3**)Cu complex.

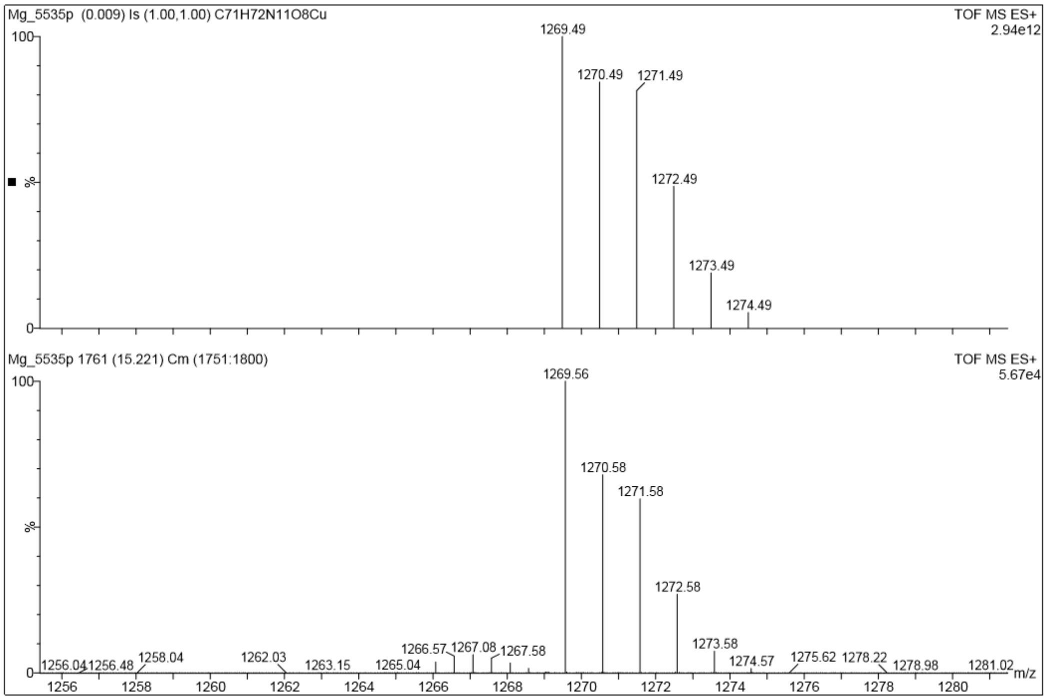

Figure S66. Experimental isotopic analysis by ESI-MS of (**Helix HQT i+3**)Cu complex (bottom) and calculated ESI-MS spectrum (top).

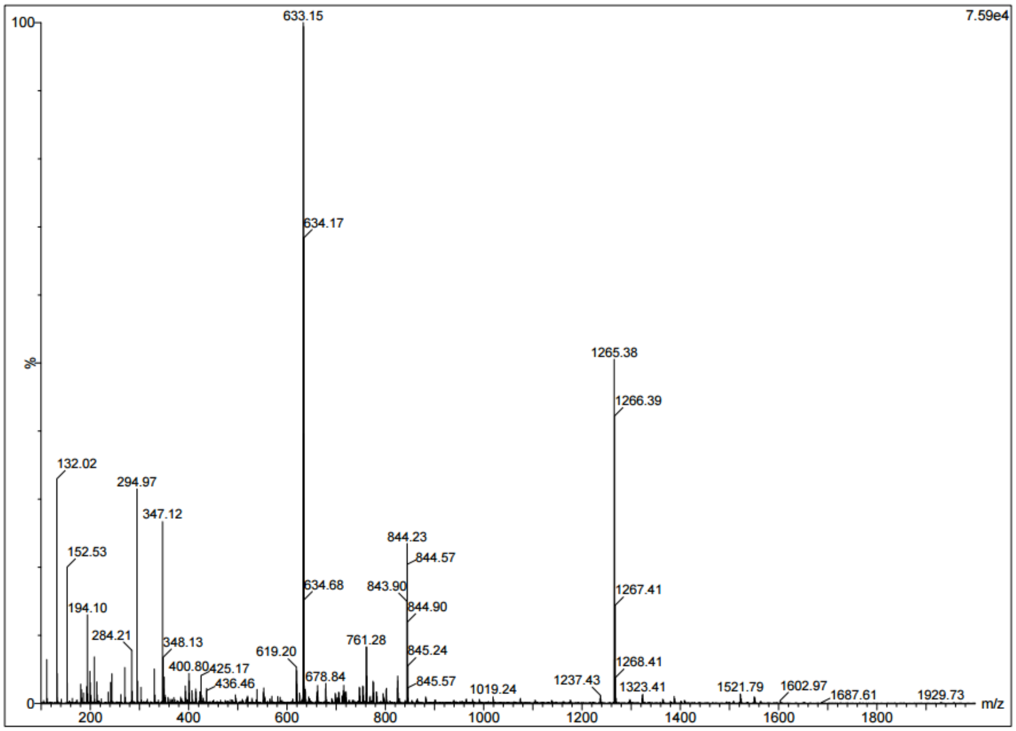

Figure S67. ESI-MS traces of (**Helix HQT i+3**)Co complex.

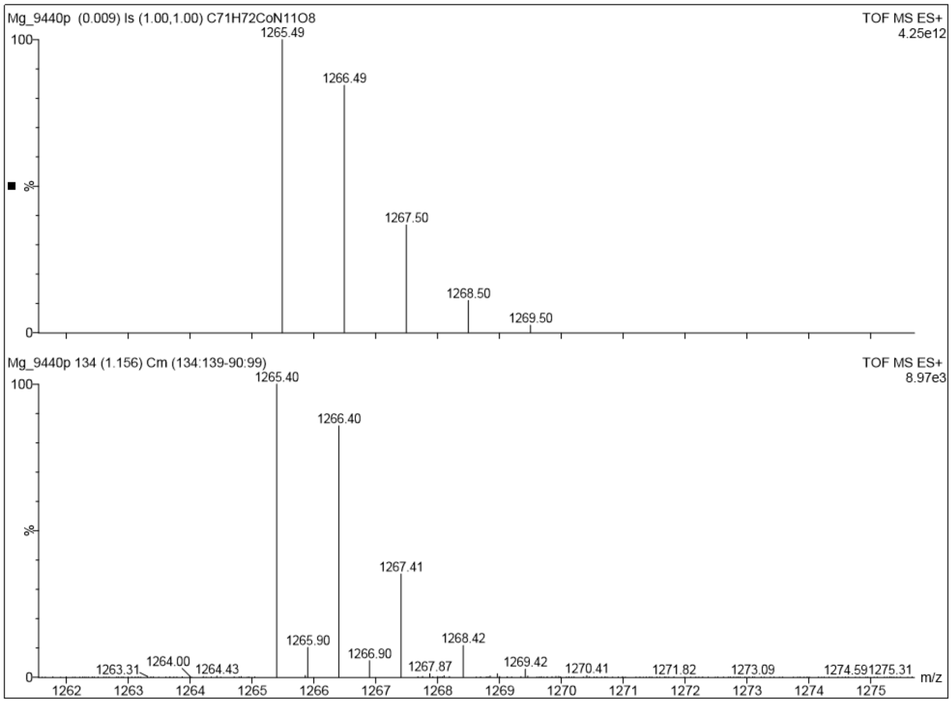

Figure S68. Experimental isotopic analysis by ESI-MS of (**Helix HQT i+3**)Co complex (bottom) and calculated ESI-MS spectrum (top).

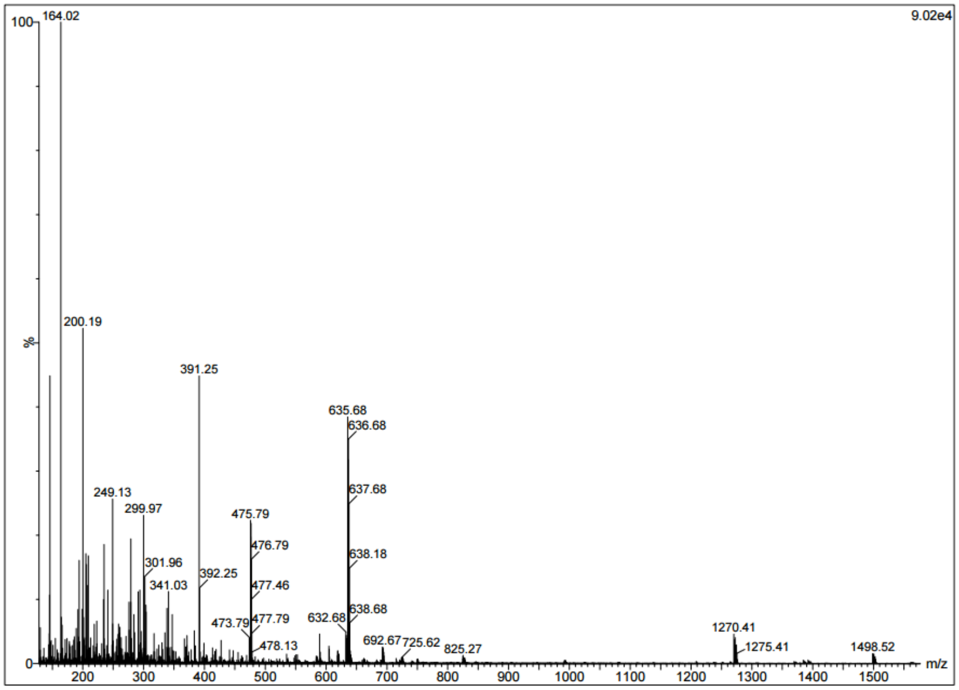

Figure S69. ESI-MS traces of (**Helix HQT i+3**)Zn complex.

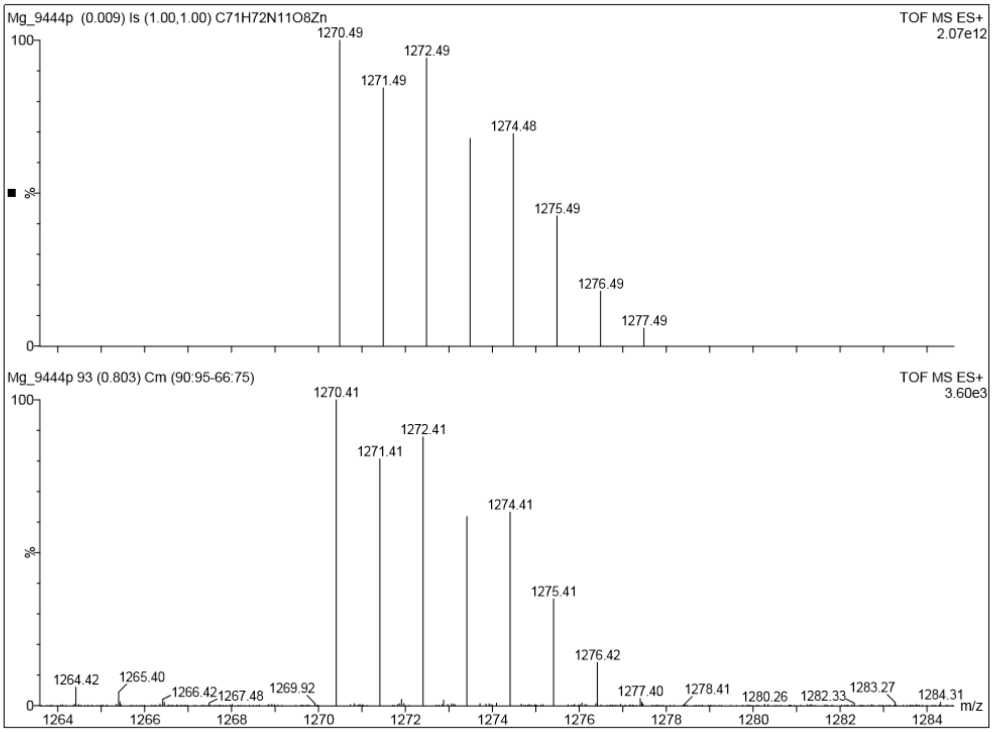

Figure S70. Experimental isotopic analysis by ESI-MS of (**Helix HQT i+3**)Zn complex (bottom) and calculated ESI-MS spectrum (top).

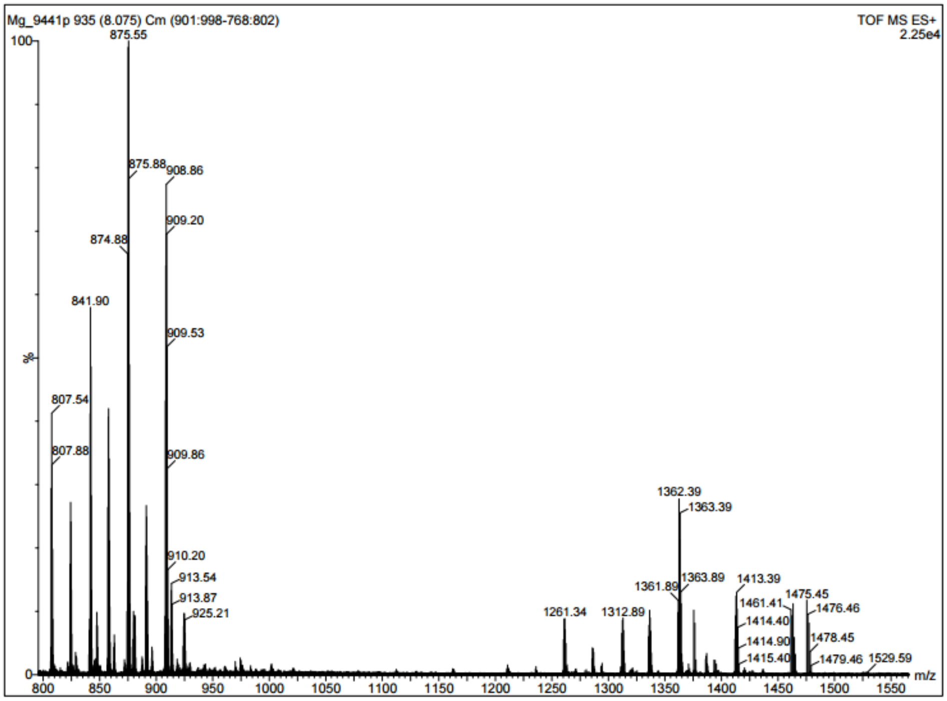

Figure S71. ESI-MS traces of (**Helix HQT i+3**)Fe complex.

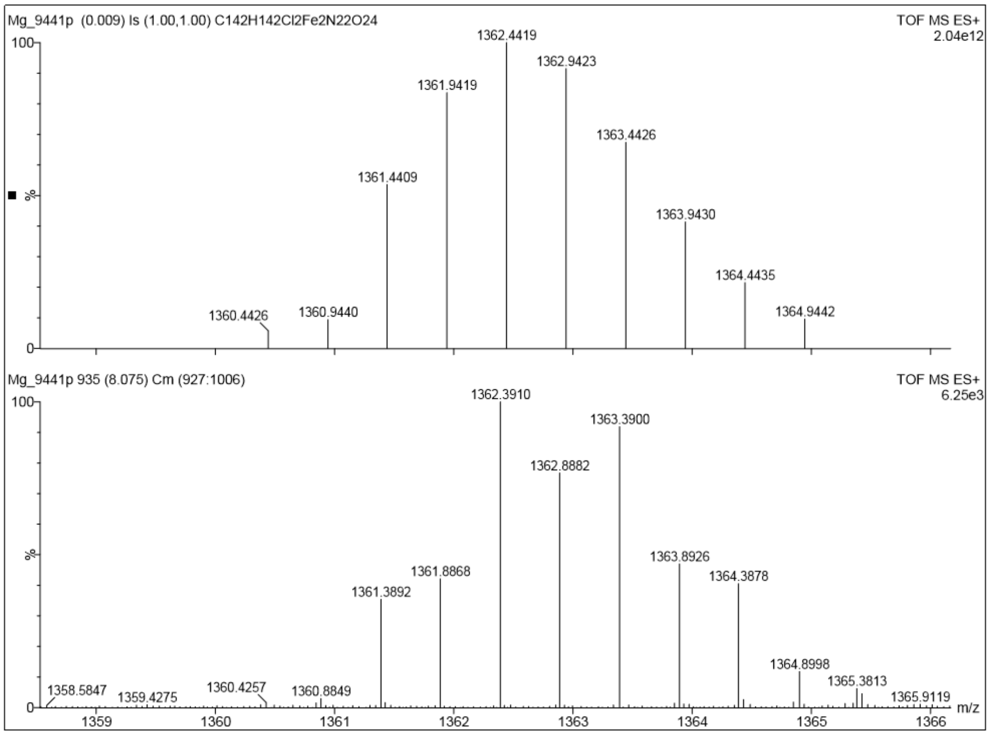

Figure S72. Experimental isotopic analysis by ESI-MS of (**Helix HQT i+3**)Fe complex (bottom) and calculated ESI-MS spectrum (top).

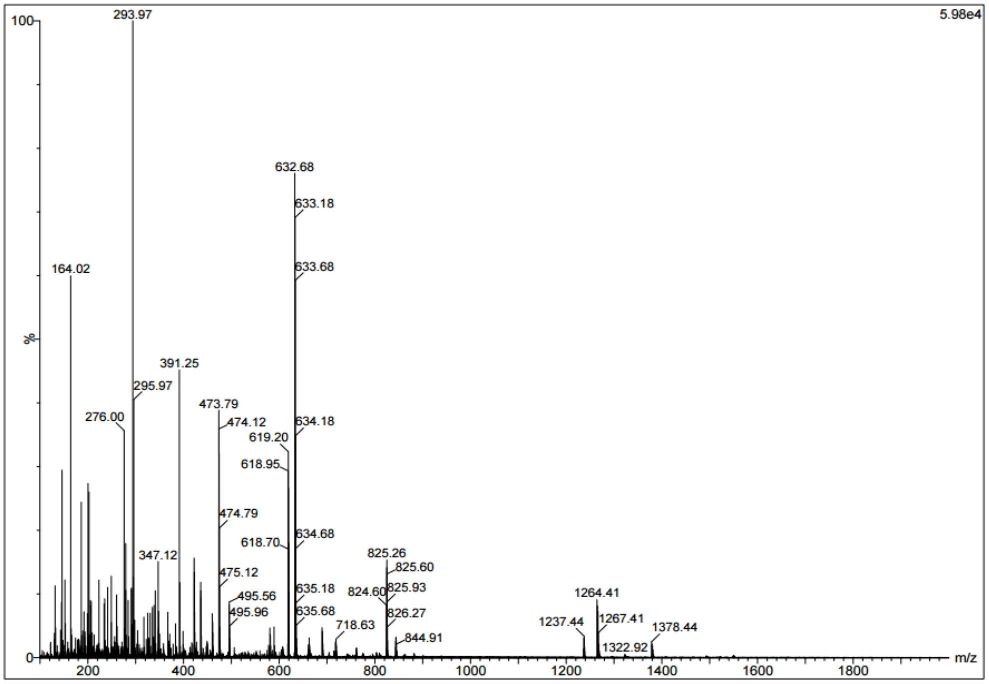

Figure S73. ESI-MS traces of (**Helix HQT i+3**)Ni complex.

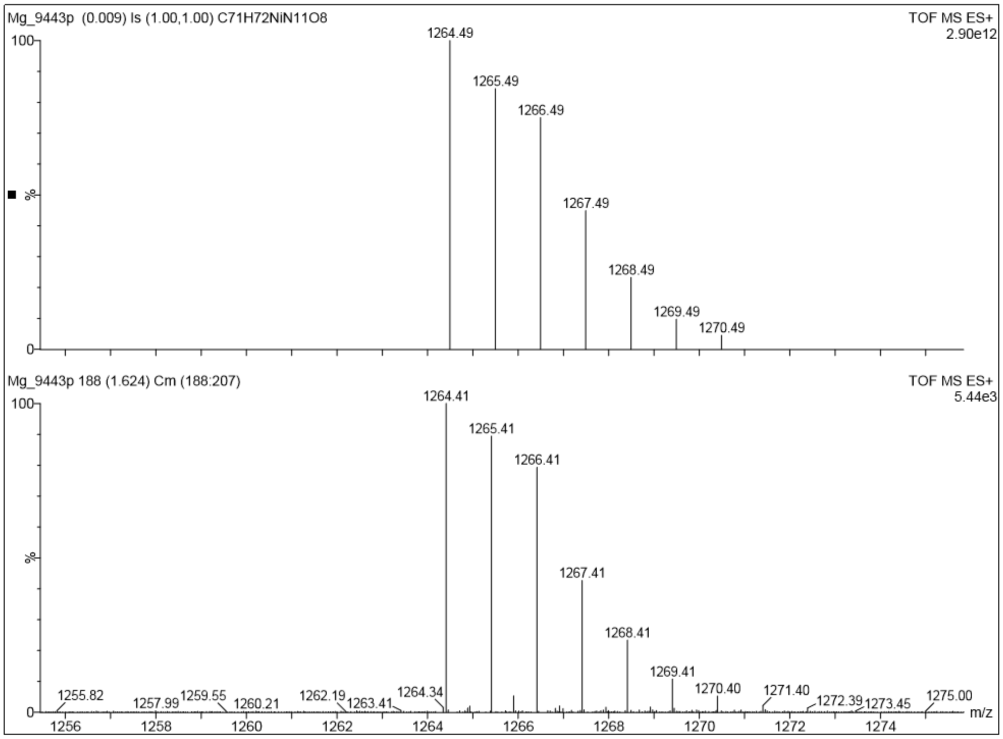

Figure S74. Experimental isotopic analysis by ESI-MS of (**Helix HQT i+3**)Ni complex (bottom) and calculated ESI-MS spectrum (top).

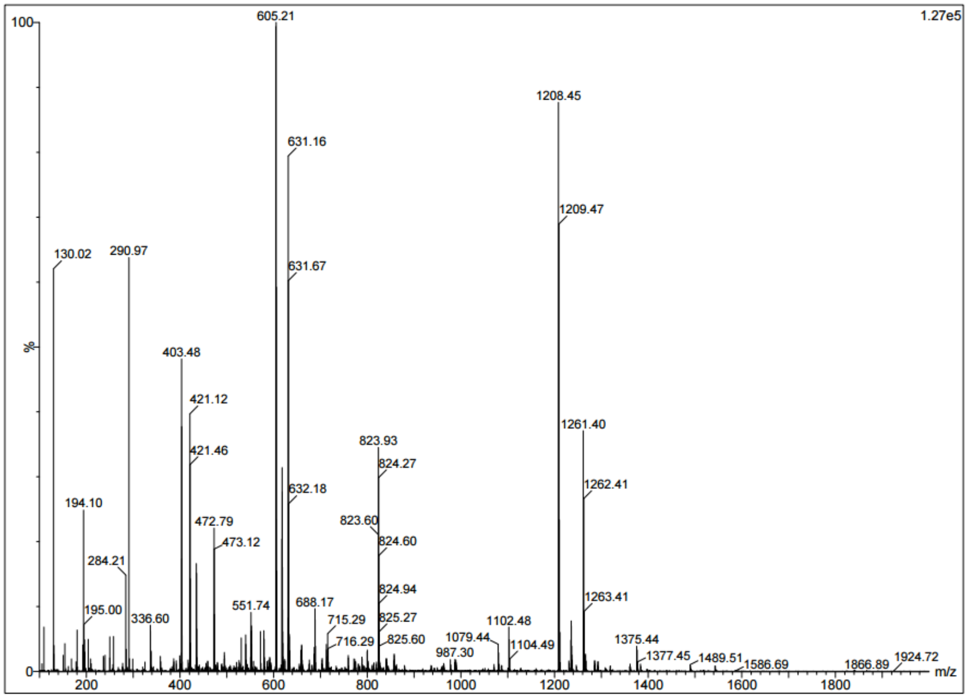

Figure S75. ESI-MS traces of (**Helix HQT i+3)**Mn complex.

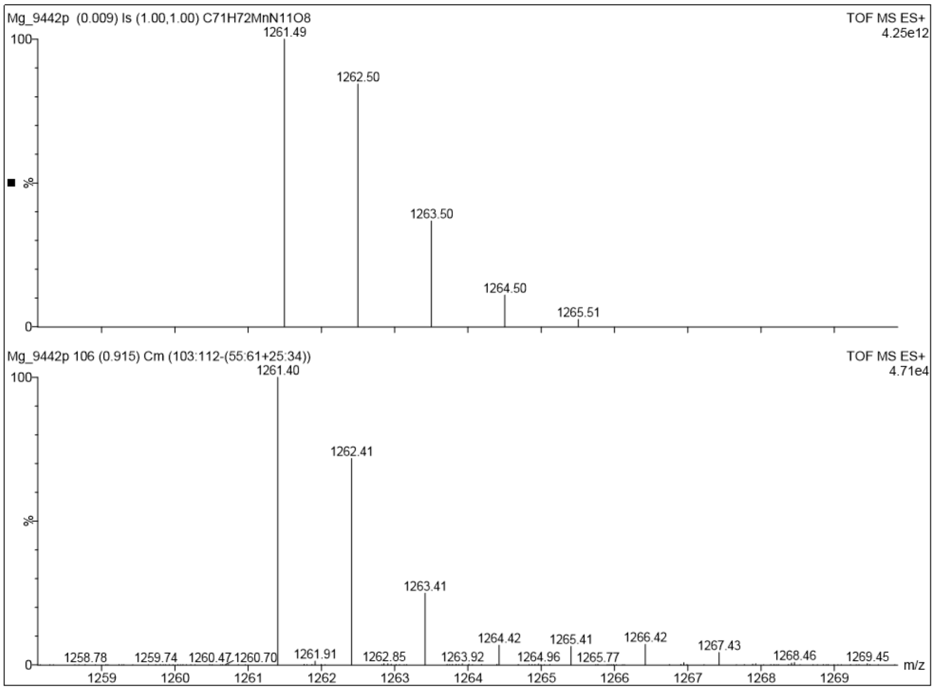

Figure S76. Experimental isotopic analysis by ESI-MS of (**Helix HQT i+3**)Mn complex (bottom) and calculated ESI-MS spectrum (top).

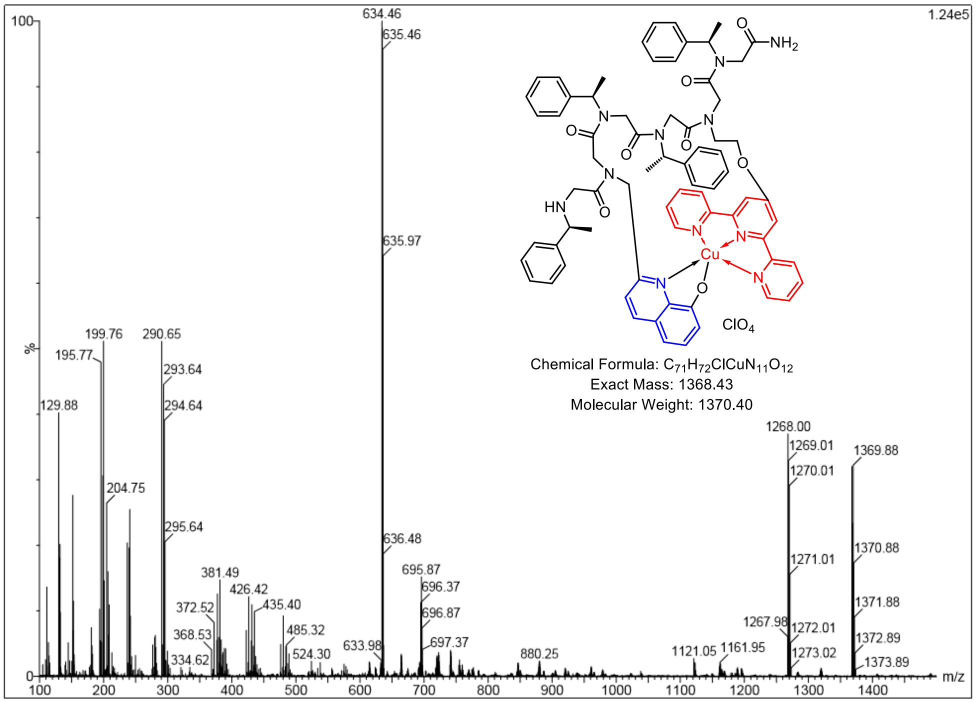

Figure S77. ESI-MS traces of peptoid oligomer **Helix HQT i+3** with 1 equiv. of Cu^{2+}, Co^{2+}, Ni^{2+}, Mn^{2+}, Fe^{3+}, Zn^{2+}.

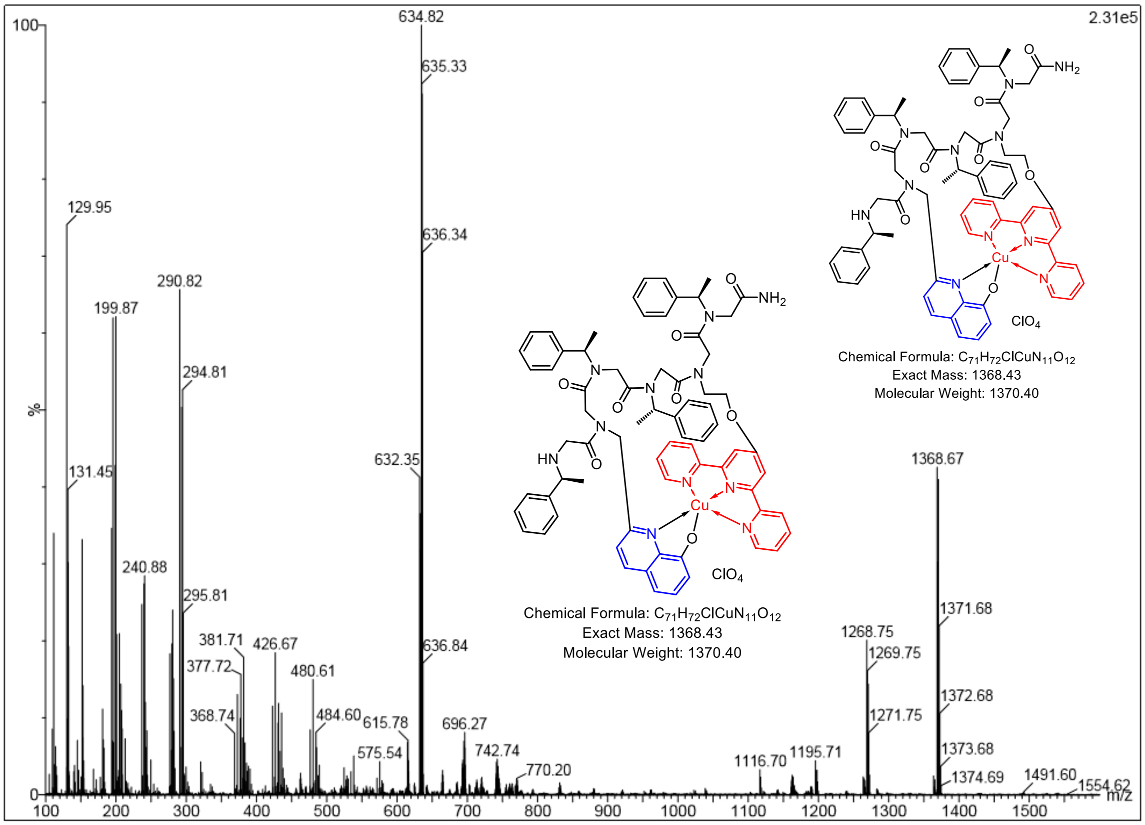

Figure S78. ESI-MS traces of peptoid oligomer **Helix HQT i+3** with 1 equiv. of Cu^{2+} and 10 equiv. Co^{2+}, Ni^{2+}, Mn^{2+}, Fe^{3+} and Zn^{2+}.

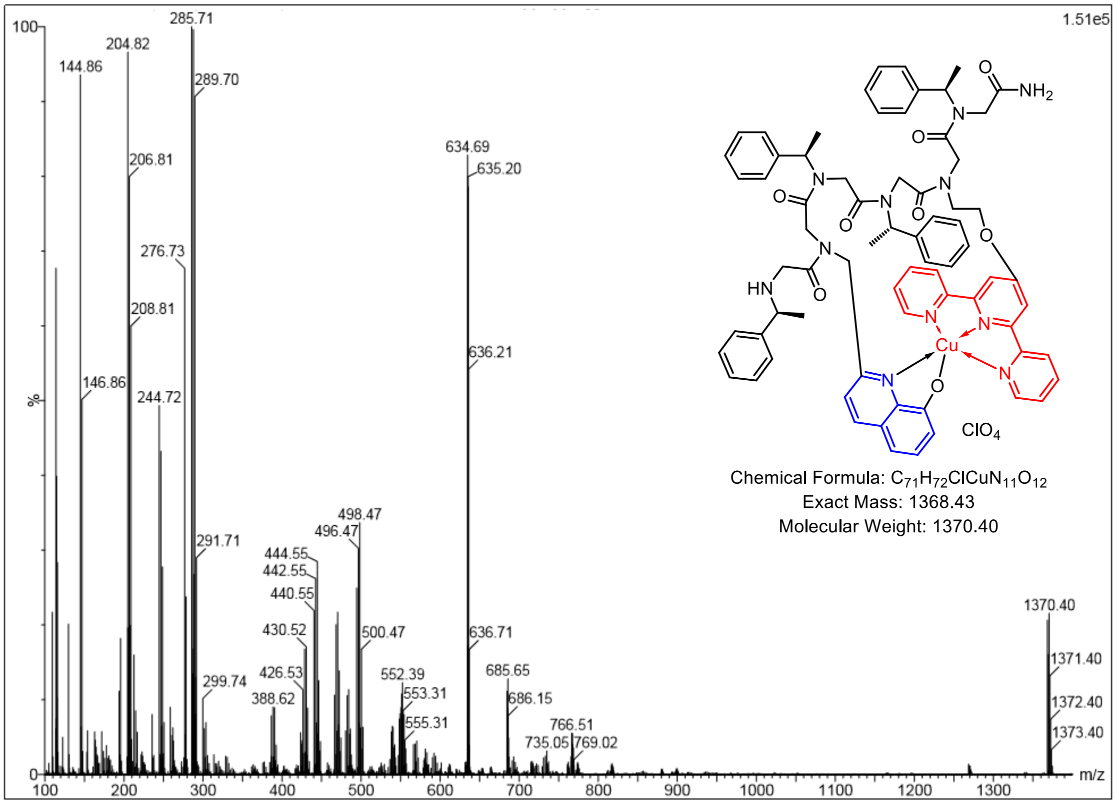

Figure S79. ESI-MS traces of peptoid oligomer **Helix HQT i+3** with 1 equiv. of Cu^{2+} and 20 equiv. Co^{2+}, Ni^{2+}, Mn^{2+}, Fe^{3+} and Zn^{2+}.

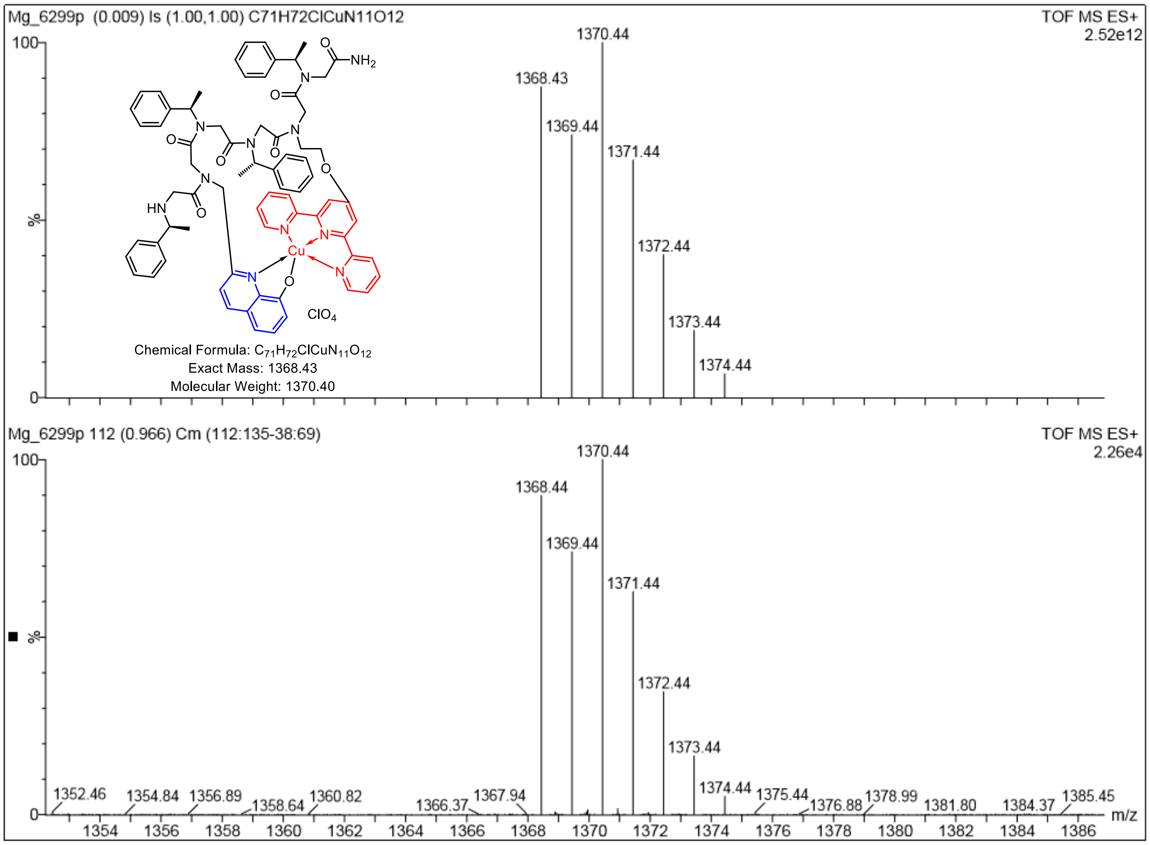

Figure S80. Experimental isotopic analysis by ESI-MS of (**Helix HQT i**+3)Cu-perchlorate complex (bottom) and calculated ESI-MS spectrum (top).

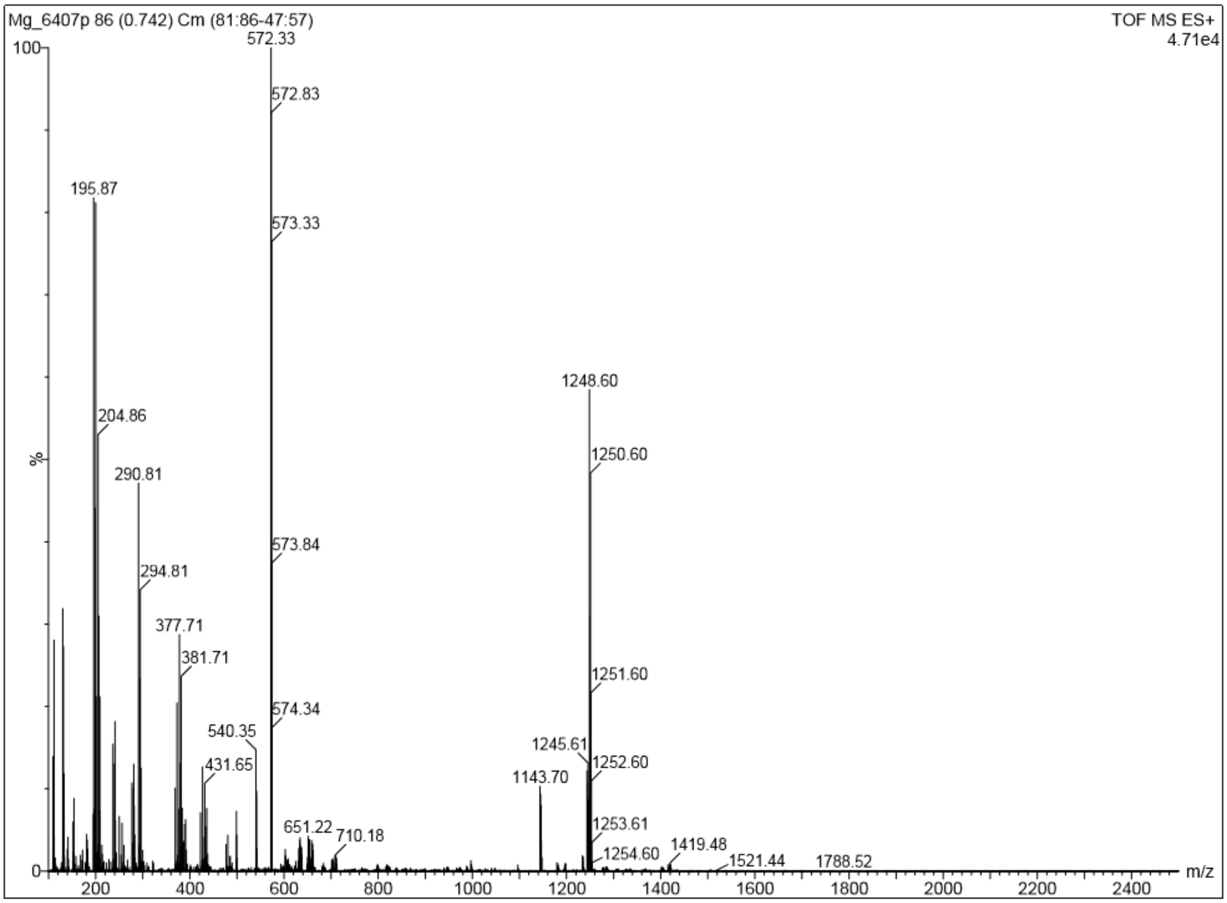

Figure S81. ESI-MS traces of peptoid oligomer **Nonhelix HQT i+3** with 1 equiv. of Cu^{2+}, Co^{2+}, Ni, Mn^{2+}, Fe^{3+} and Zn^{2+}.

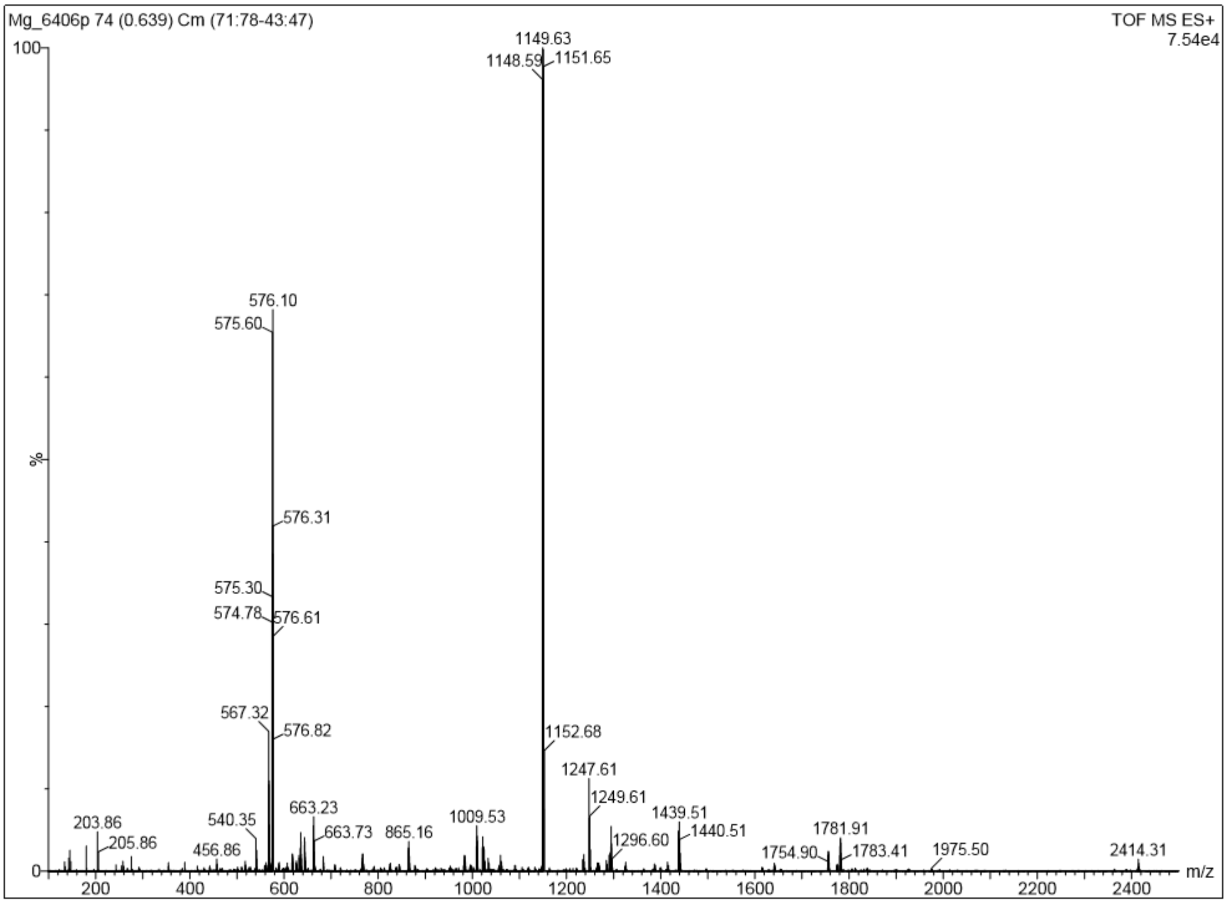

Figure S82. ESI-MS traces of peptoid oligomer **Nonhelix HQT i+3** with 1 equiv. of Cu^{2+}.

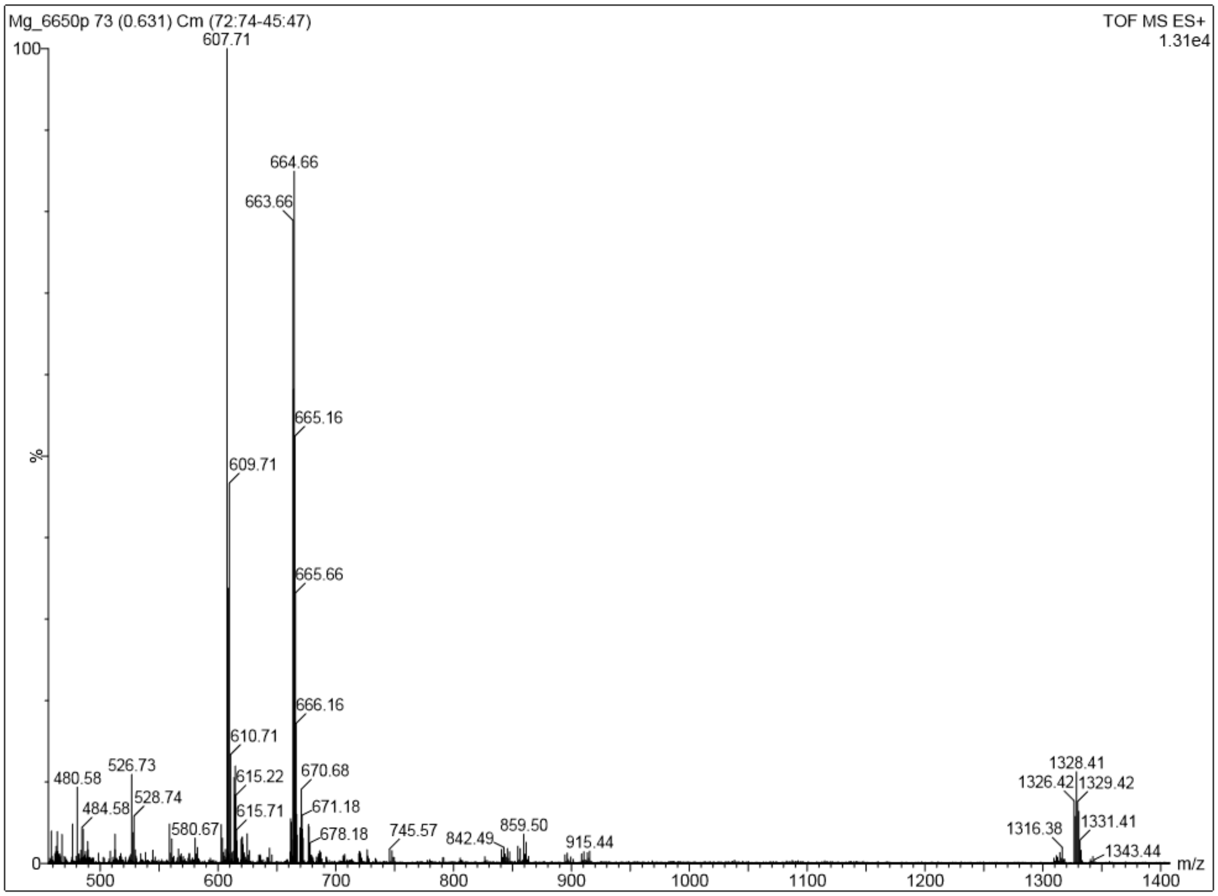

Figure S83. ESI-MS traces of peptoid oligomer **DI HQT** with 1 equiv. of Cu^{2+}, Co^{2+}, Ni^{2+}, Mn^{2+}, Fe^{3+} and Zn^{2+}.

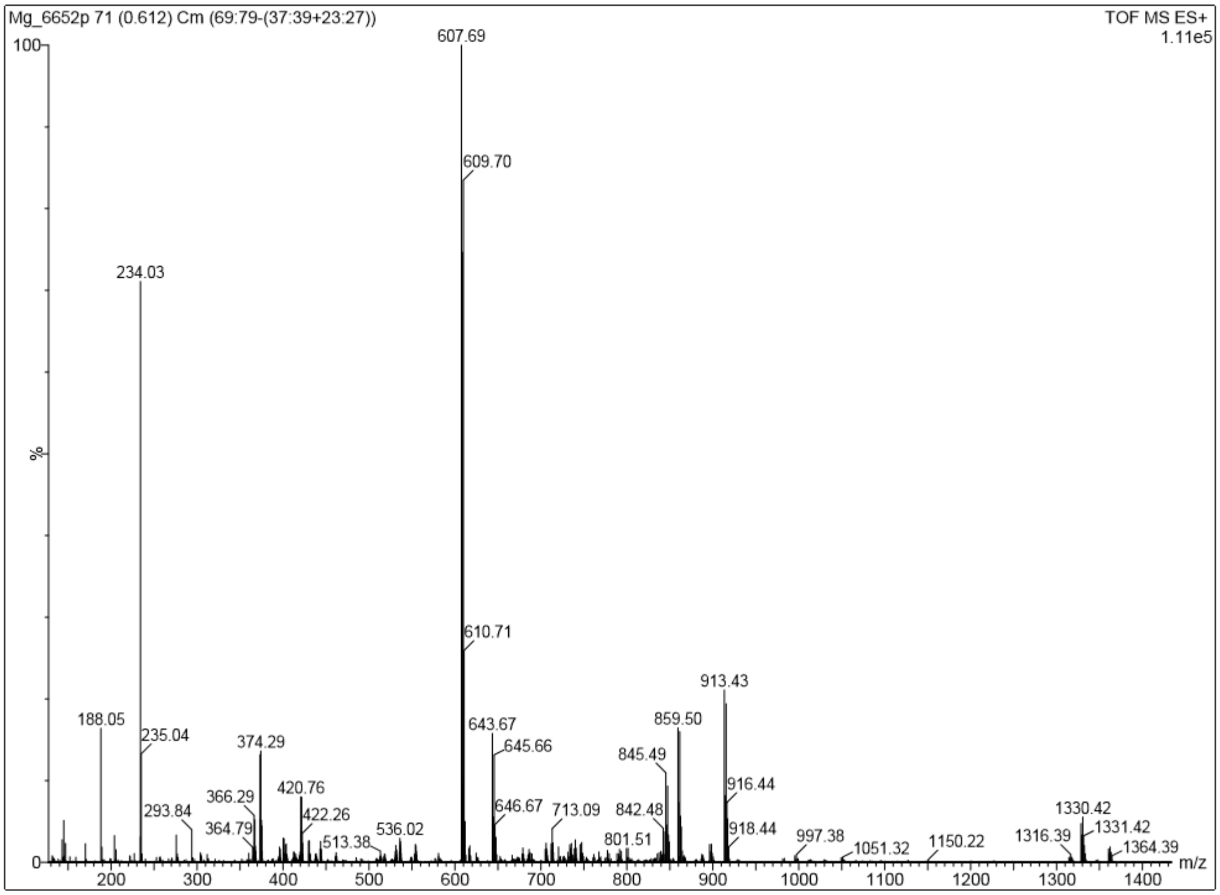

Figure S84. ESI-MS traces of peptoid oligomer **DI HQT** with 1 equiv. Cu^{2+}.

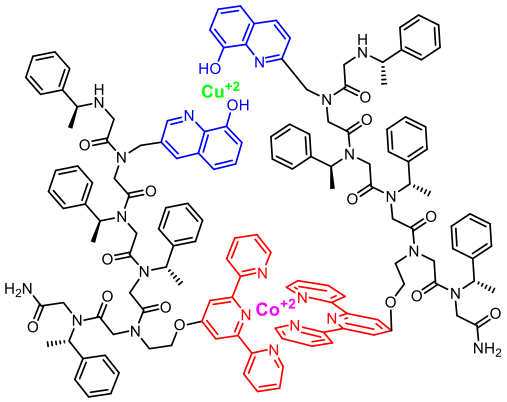

Chemical Formula: $C_{142}H_{146}CoCuN_{22}O_{16}{}^{4+}$
Exact Mass: 2536.99
Molecular Weight: 2539.29

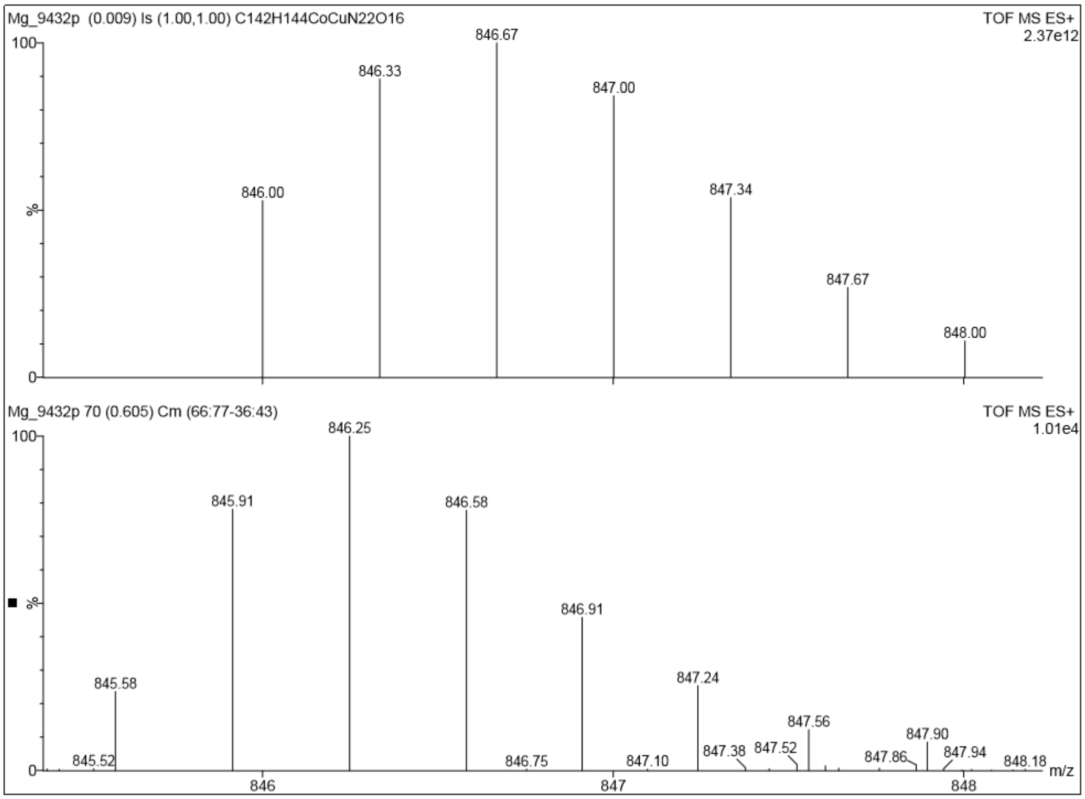

Figure S85. Experimental isotopic analysis by ESI-MS m/3z of (**Helix HQT i+3**)$_2$CoCu hetero bi-metallic peptoid complex synthesized by step approach (bottom) and calculated ESI-MS spectrum (top).

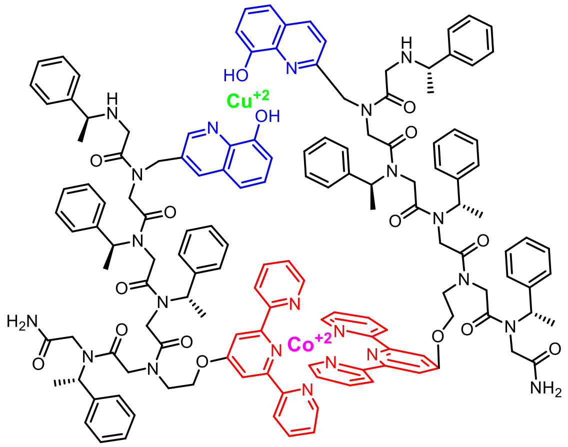

Chemical Formula: $C_{142}H_{146}CoCuN_{22}O_{16}^{4+}$
Exact Mass: 2536.99
Molecular Weight: 2539.29

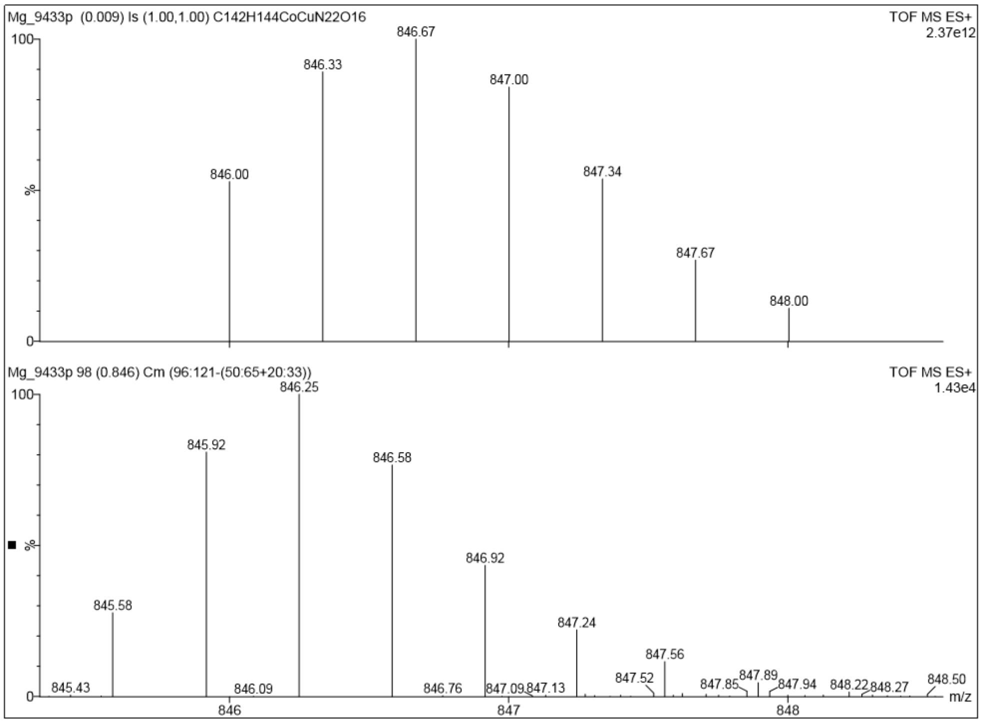

Figure S86. Experimental isotopic analysis by ESI-MS m/3z traces of (**Helix HQT i and i+3**)$_2$CoCu hetero bi-metallic peptoid duplex synthesized by mix approach (bottom) and calculated ESI-MS spectrum (top).

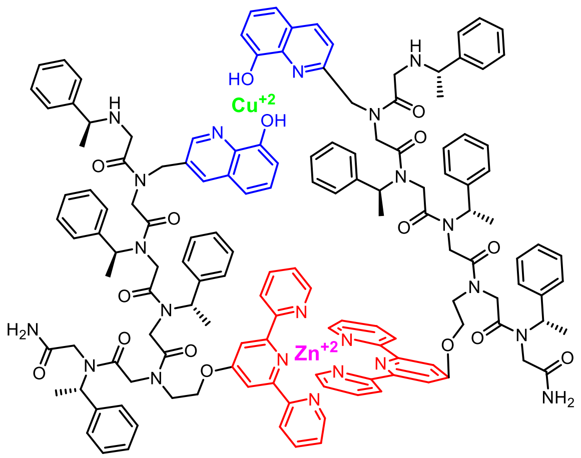

Chemical Formula: $C_{142}H_{146}CuN_{22}O_{16}Zn^{4+}$
Exact Mass: 2541.99
Molecular Weight: 2545.74

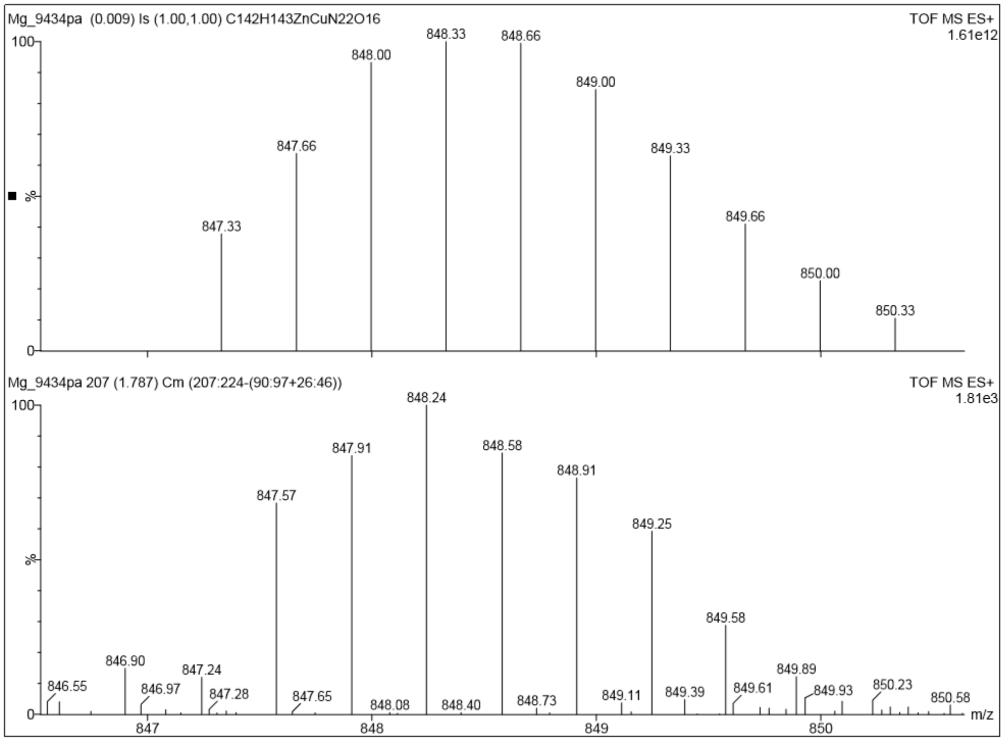

Figure S87. Experimental isotopic analysis by ESI-MS m/3z traces of (**Helix HQT i+3**)$_2$ZnCu hetero bi-metallic peptoid duplex synthesized by step approach (bottom) and calculated ESI-MS spectrum (top).

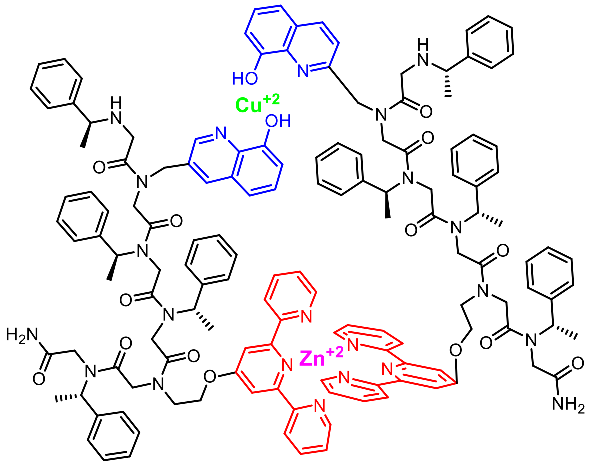

Chemical Formula: $C_{142}H_{146}CuN_{22}O_{16}Zn^{4+}$
Exact Mass: 2541.99
Molecular Weight: 2545.74

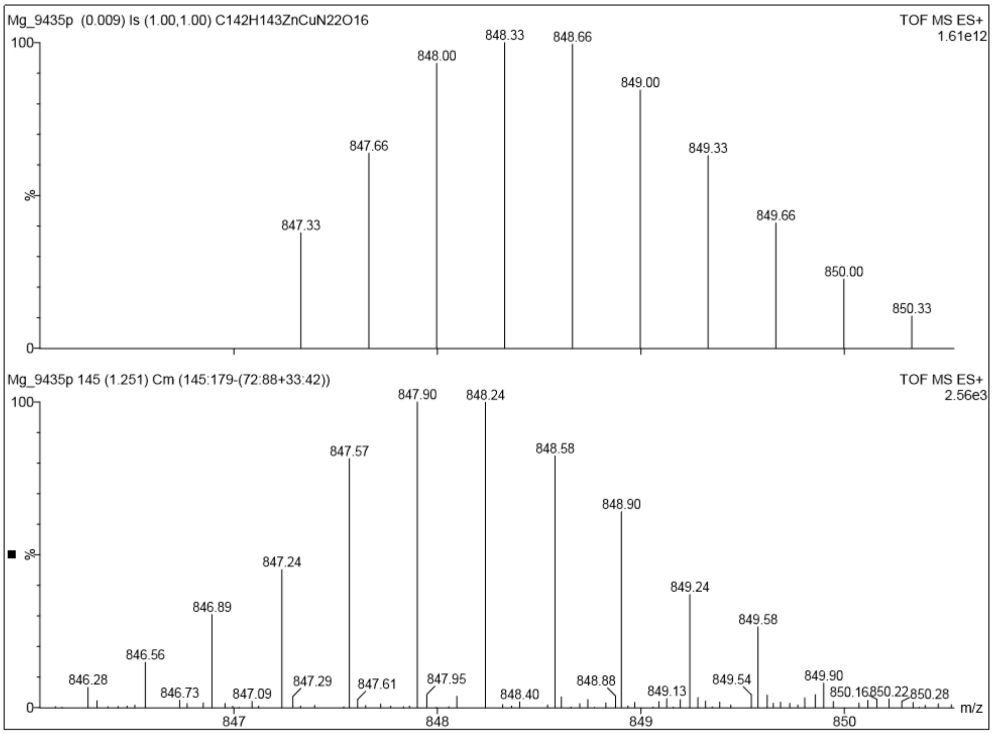

Figure S88. ESI-MS m/3z traces of (**Helix HQT i+3**)$_2$ZnCu hetero bi-metallic peptoid duplex synthesized by mix approach (bottom) and calculated ESI-MS spectrum (top).

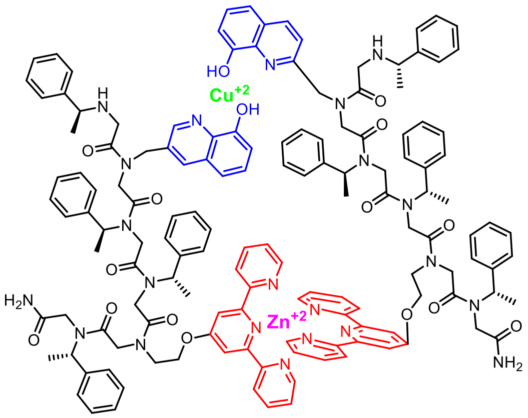

Chemical Formula: $C_{142}H_{146}CuN_{22}O_{16}Zn^{4+}$
Exact Mass: 2541.99
Molecular Weight: 2545.74

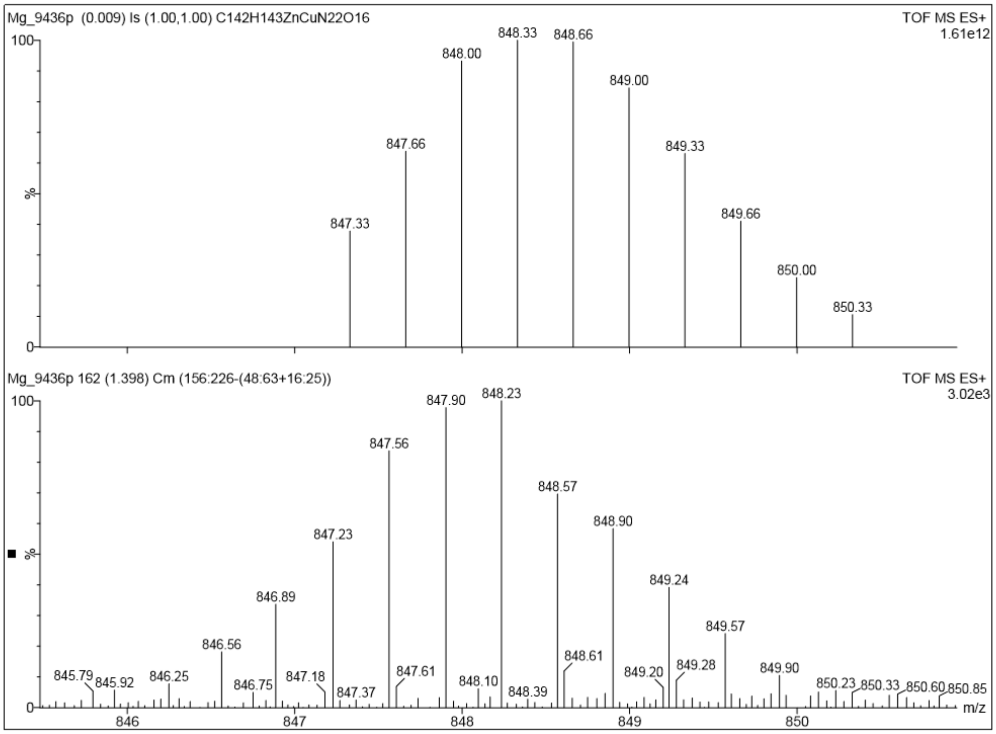

Figure S89. Experimental isotopic analysis by ESI-MS m/3z traces of (**Helix HQT i+3**)$_2$ZnCu hetero bi-metallic peptoid duplex synthesized by step approach for EPR analysis (bottom) and calculated ESI-MS spectrum (top).

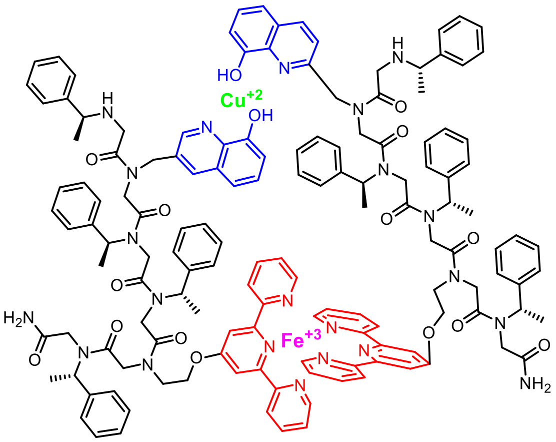

Chemical Formula: $C_{142}H_{146}CuFeN_{22}O_{16}^{5+}$
Exact Mass: 2533.99
Molecular Weight: 2536.20

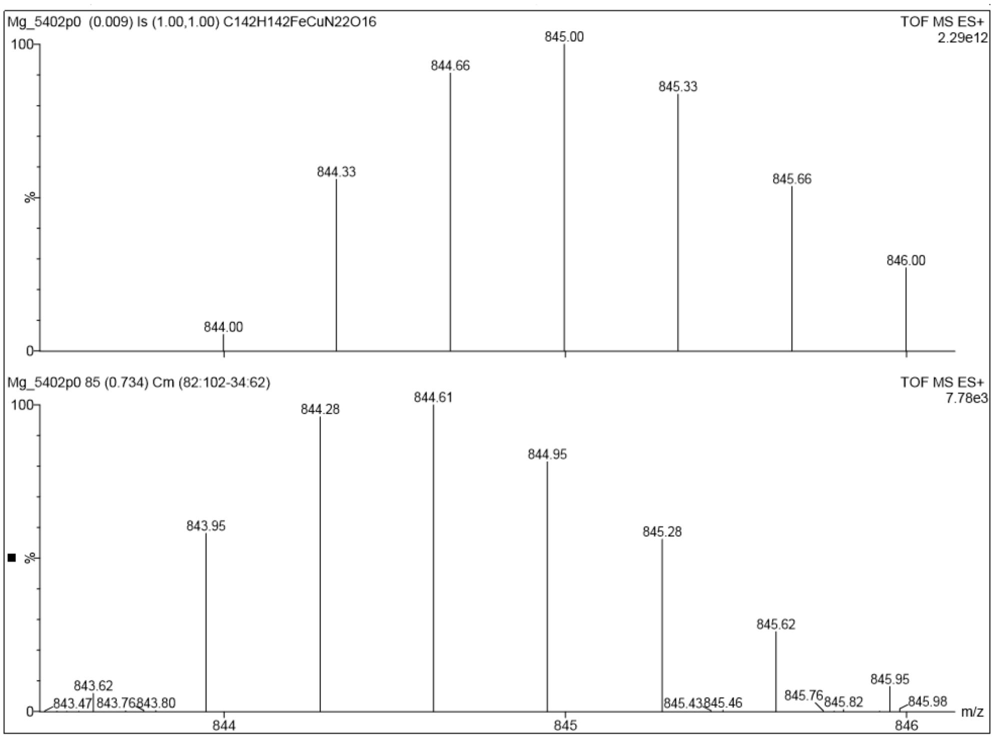

Figure S90. Experimental isotopic analysis by ESI-MS m/3z traces of (**Helix HQT i**+3)$_2$FeCu hetero bi-metallic peptoid duplex synthesized by mix approach at 50 ^{0}C for 2 hours (bottom) and calculated ESI-MS spectrum (top).

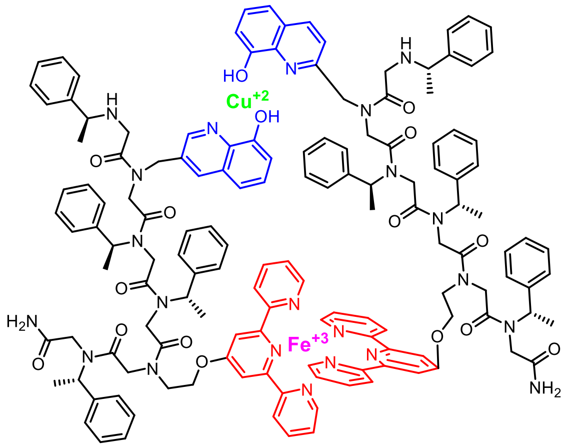

Chemical Formula: $C_{142}H_{146}CuFeN_{22}O_{16}^{5+}$
Exact Mass: 2533.99
Molecular Weight: 2536.20

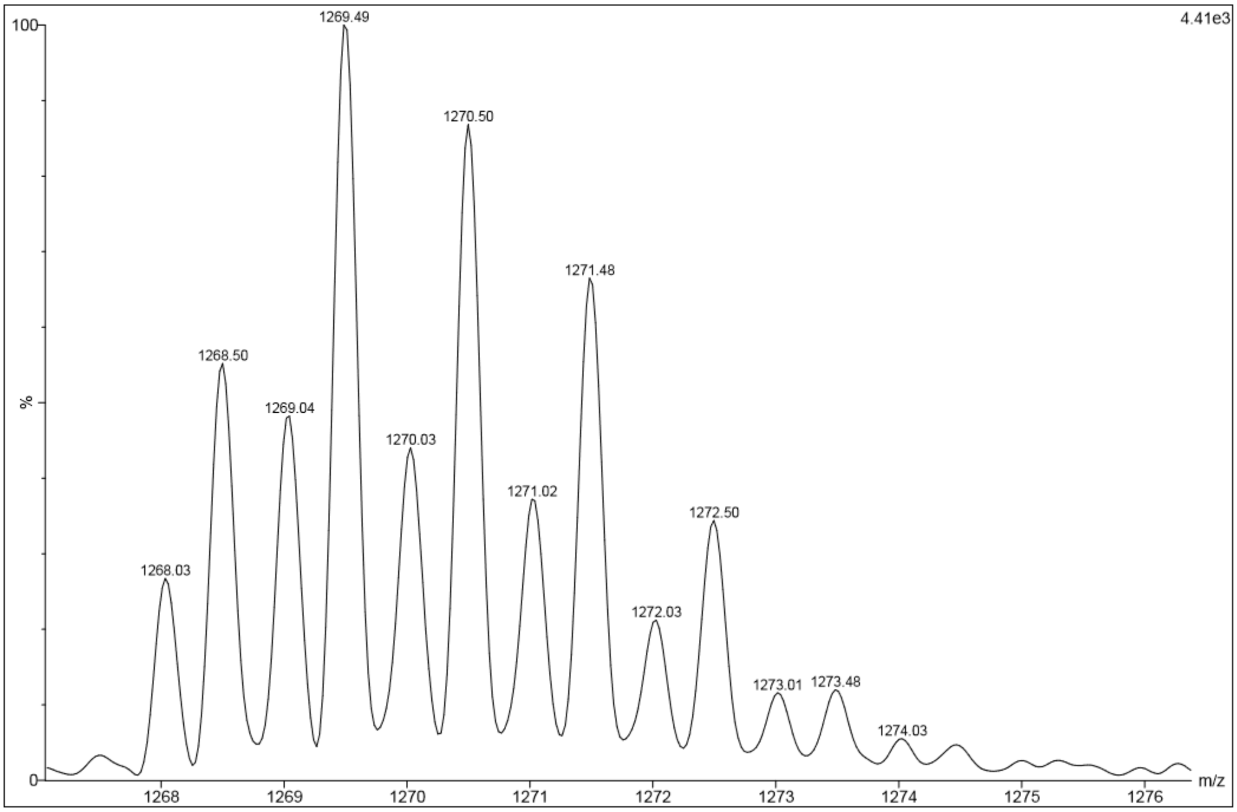

Figure S91. MALDI m/2z spectrum of (**Helix HQT i+3**)$_2$FeCu hetero bi-metallic peptoid duplex synthesized by step approach.
